# Therapeutic Gases in Biomedicine: Updates on Nitric Oxide and Beyond

**DOI:** 10.1002/advs.202521942

**Published:** 2026-03-20

**Authors:** Syed Muntazir Andrabi, Navatha Shree Sharma, Imran Ibn Gani Rather, S. M. Shatil Shahriar, Jingwei Xie

**Affiliations:** ^1^ Department of Surgery‐Transplant and Mary and Dick Holland Regenerative Medicine Program University of Nebraska Medical Center Omaha Nebraska USA; ^2^ Department of Mechanical and Materials Engineering University of Nebraska Lincoln Lincoln Nebraska USA

**Keywords:** biomedicine, delivery, donors, gaseous mediators, nitric oxide, physiological functions

## Abstract

Gaseous mediators are increasingly recognized as critical regulators of human physiology and pathology, offering unique therapeutic opportunities that are fundamentally constrained by challenges in controlled delivery, spatial targeting, and biological specificity when harnessed through advanced delivery technologies. Among them, nitric oxide (NO) has emerged as a central player. Recent advancements have transitioned from conventional donor‐based systems with limited control over dose and localization to wearable gas‐delivery platforms capable of real‐time, patient‐specific dosing and frequently coupling with embedded diagnostic functionalities for dynamic feedback and precision control. Importantly, the therapeutic scope of gas biology is expanding beyond NO to encompass other clinically relevant and emerging gases, including O_2_, CO, H_2_S, H_2_, CO_2_, SO_2_, and Xe. These gases exhibit multifaceted biological effects ranging from cytoprotection and vasoregulation to antimicrobial and anti‐inflammatory actions, thereby necessitating innovative material‐based and bioresponsive delivery strategies that can interface dynamically with biological systems. By critically assessing progress in material platforms, responsive release mechanisms, and multimodal delivery approaches, this review bridges fundamental gas biology with clinically translatable therapeutic design. We further highlight persistent translational bottlenecks, such as dose control, off‐target effects, and long‐term safety, while outlining emerging research pathways poised to define the next era of gas‐based medicine.

## Graphical abstract

Therapeutic gases, including NO, CO, H_2_S, H_2_, CO_2_, O_2_, and Xe, play vital roles in cellular signaling and repair. This review highlights the emerging carriers and delivery systems that enable controlled, localized gas release for diagnostic and therapeutic applications.

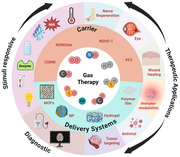

## 1 Introduction

Gaseous signaling molecules, once dismissed as incidental metabolic by‐products or environmental irritants, are now firmly established as pivotal regulators of physiological and pathological processes [1]. Among them, nitric oxide (NO), carbon monoxide (CO), hydrogen sulfide (H_2_S), and oxygen (O_2_) have emerged as prototypical gasotransmitters as small and endogenously produced molecules that modulate vascular tone, neurotransmission, immune function, and metabolic homeostasis [1, 2, 3, 4, 5]. In parallel, sulfur dioxide (SO_2_), hydrogen (H_2_), and carbon dioxide (CO_2_), though historically overlooked in the biomedical context, have gained increasing attention for their capacity to control inflammation, promoting tissue regeneration, and modulating tumors [6, 7]. The therapeutic exploitation of such gases now represents an expanding interdisciplinary frontier that integrates chemical biology, materials science, bioengineering, and translational medicine.

Despite their compelling biological potential, the clinical translation of gas‐based therapeutics remains constrained by intrinsic physicochemical challenges [5, 8, 9]. Their volatility, high reactivity, and rapid degradation hinder precise dosing, long‐term stability, and sustained site‐specific delivery. Conventional administration methods often lack spatial and temporal control, risking systemic toxicity or insufficient local concentration [5, 10]. In our earlier review, we comprehensively discussed and focused primarily on NO's physiological roles, various types of donors and carrier platforms, and biomedical applications [11]. The field, however, has since undergone transformative progress. In this updated work, we revisit these delivery modalities, highlighting newly emergent strategies ranging from small‐molecule prodrugs and photocaged donors to next‐generation metal‐organic frameworks (MOFs) and other evolving applications. These platforms are increasingly engineered for programmable, stimuli‐triggered release, multi‐gas co‐delivery, integration with wearable diagnostics, and synergy with combinatorial therapeutic regimens [1, 12, 13, 14, 15, 16, 17, 18].

Across therapeutic contexts, gaseous mediators are governed by shared physicochemical constraints that fundamentally shape their clinical utility. Small molecular size, high diffusivity, and chemical reactivity enable rapid signaling but simultaneously impose challenges in dose precision, spatial confinement, and temporal control. For example, highly reactive gases such as NO and H_2_S exhibit narrow therapeutic windows, where subtle changes in concentration or exposure time can shift effects from cytoprotective to cytotoxic [5, 8, 9, 10]. Conversely, relatively inert gases such as xenon (Xe) or molecular hydrogen require strategies to overcome poor solubility and rapid clearance to achieve sustained bioactivity [6]. These intrinsic properties necessitate engineering‐driven delivery solutions, including donor chemistry to stabilize reactive species, encapsulation and confinement to modulate diffusion, catalytic and prodrug‐based systems for in situ generation, and stimulus‐responsive materials to synchronize release with disease microenvironments [1, 11, 12, 13, 14, 15, 16, 17, 18]. Viewed through this lens, modern gas therapeutics are no longer defined solely by biological activity, but by the rational integration of gas physicochemistry with material design, device architecture, and feedback‐controlled delivery. This structure‐property‐function paradigm provides a unifying framework for understanding recent advances in gas‐based medicine and underpins the organization of this review.

Here, we expand the discussion to encompass therapeutic areas previously unexplored, highlighting NO's roles in the management of viral infections, ocular disorders, nerve regeneration, and immunomodulation. Furthermore, we address the advent of wearable gas‐delivery devices capable of real‐time, patient‐specific dosing with embedded diagnostic functionality. Beyond NO, we provide an integrative perspective on other clinically relevant and emerging therapeutic gases, including O_2_, CO, H_2_S, H_2_, SO_2_, CO_2_, and Xe, outlining their biological roles, delivery strategies, and therapeutic outcomes. Through a critical synthesis of recent advances in material platforms, bioresponsive release systems, and multimodal delivery approaches, this review aims to bridge mechanistic insights in gas biology with the design of clinically translatable therapeutics, identifying both the persistent translational bottlenecks and the emerging research pathways likely to define the next era of gas‐based medicine.

**SCHEME 1:**
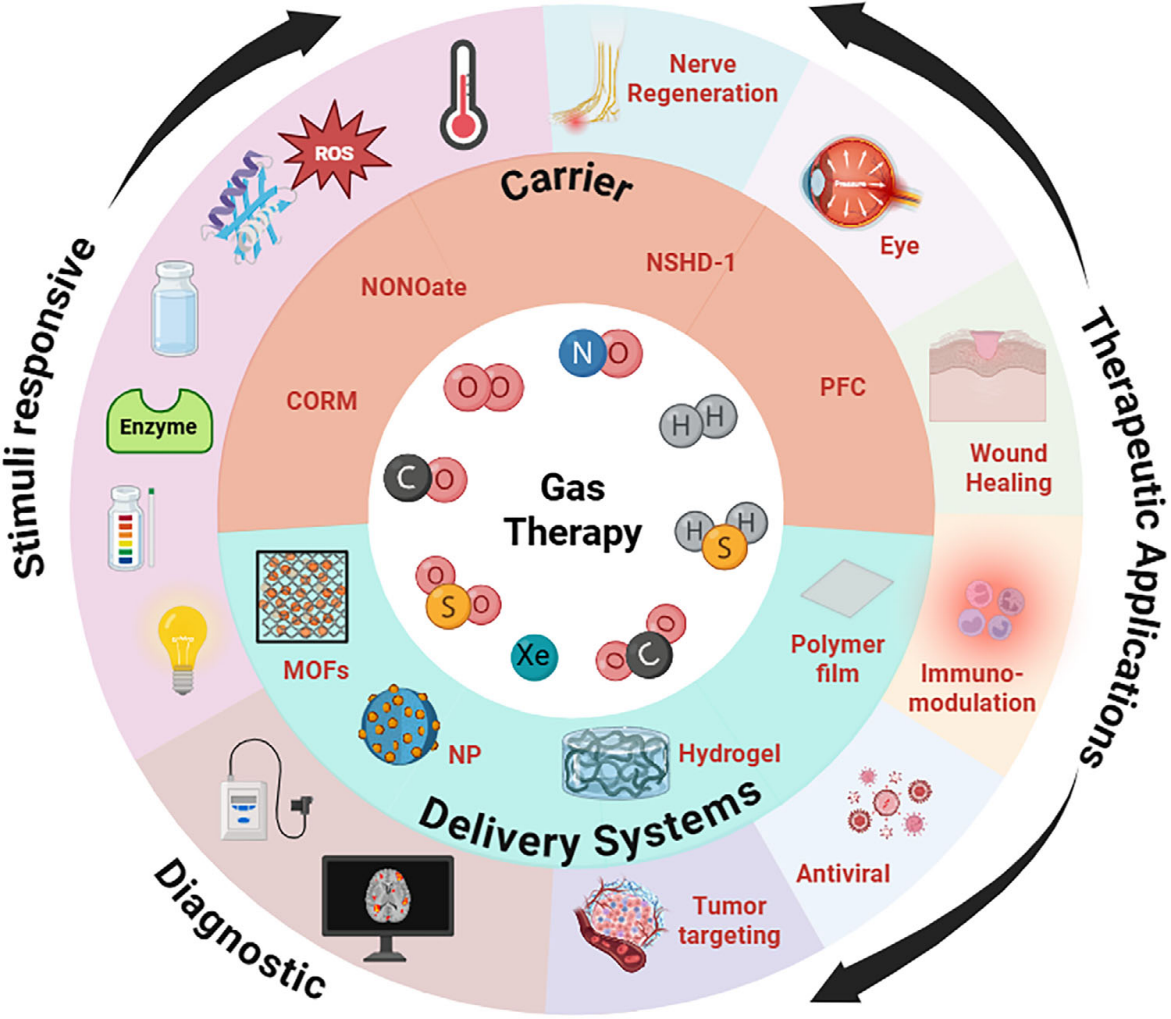
The schematic highlights the emerging carriers and delivery systems that enable controlled, localized gas release for diagnostic and therapeutic applications.

While several prior reviews have summarized the biological roles and donor chemistries of individual gasotransmitters, most notably nitric oxide, these works have largely focused on isolated gases, single delivery modalities, or phenomenological biological effects [8, 10, 11, 13]. In contrast, the present review adopts an engineering‐centered and systems‐level perspective, emphasizing how physicochemical constraints of therapeutic gases dictate material design, device architecture, and delivery strategy. Beyond nitric oxide, we provide an integrated discussion of emerging therapeutic gases, including O_2_, CO, H_2_S, H_2_, SO_2_, CO_2_, and Xe, which have been underrepresented or treated separately in earlier reviews. Importantly, this work highlights recent advances in programmable delivery platforms, catalytic and bioinspired systems, wearable and feedback‐controlled devices, and theranostic approaches, positioning gas‐based therapies within a unified translational framework rather than as isolated chemical interventions (Scheme 1).

## 2 Delivery and Carrier (Update)

**FIGURE 1:**
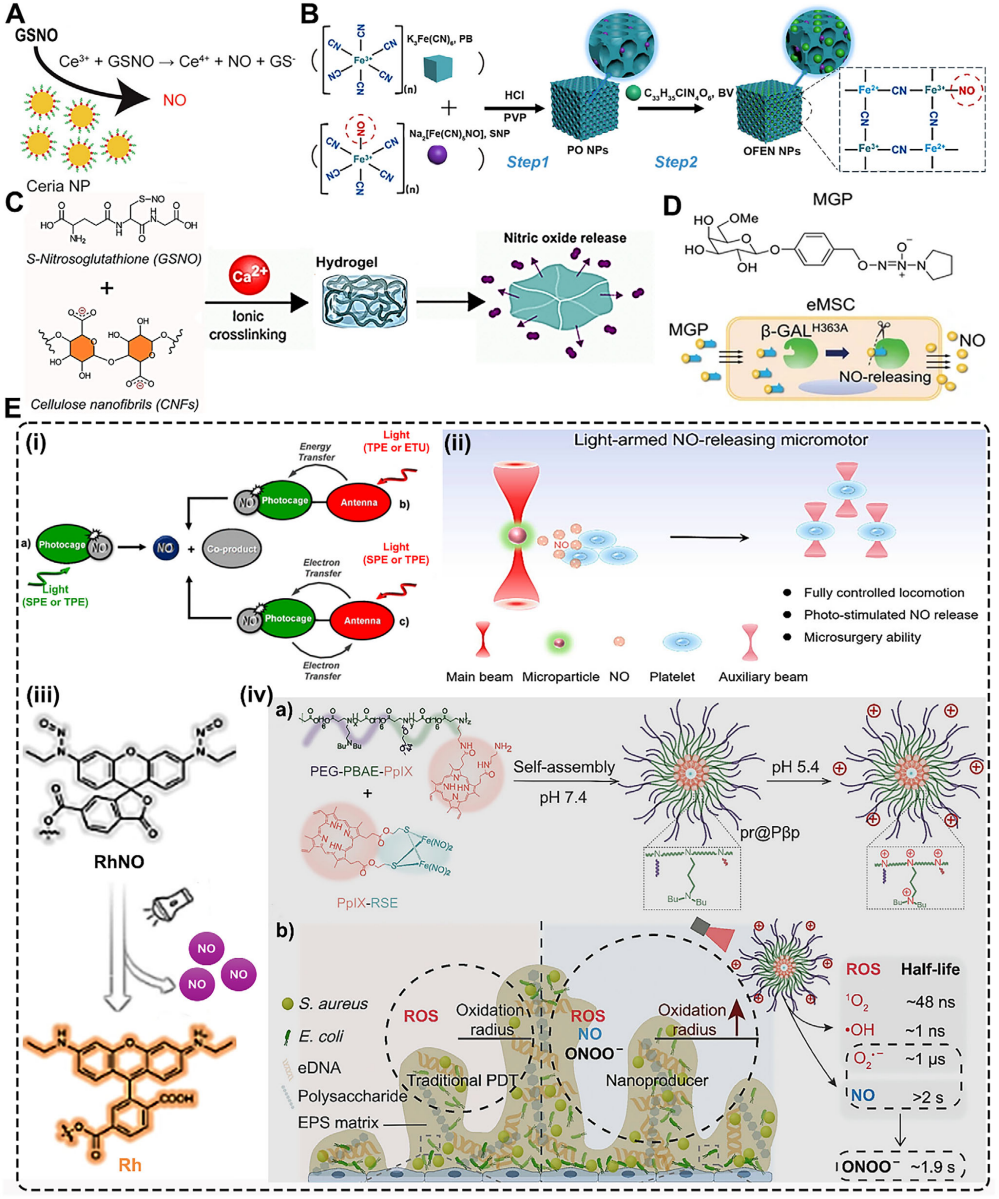
NO donors and carriers. (A) Schematic illustration of the mechanism of NO generation from GSNO by the catalytic activity of ceria NP. Reproduced with permission [20]. Copyright 2022, Wiley VCH. (B) Synthesis of ONOO^–^‐free NO‐embedded nanomedicine (OFENs). Reproduced with permision [21]. Copyright 2024, American Chemical Society. (C) Overview of the preparation of Ca^2+^ cross‐linked CNF hydrogel and release of NO Reproduced with permission [30]. Copyright 2024, American Chemical Society. (D) Structure of the NO prodrug MGP, 6‐methyl‐galactose‐benzyl‐oxy NONOate, and construction of the NO‐eMSC system and the mechanism of NO release from this system. Adapted under the Creactive Commons Attribution License [39]. Copyright 2023 (E) (i) Schematic diagram of the SPE, TPE, and ETU processes, which can be exploited to trigger the NO release. Reproduced with permission [16]. Copyright 2024, American Chemical Society. (ii) light‐armed NO‐releasing micromotor (LaNorM). Reproduced with permission [55]. Copyright 2024, American Chemical Society. (iii) Schematic diagram of RhNO turn‐on to a fluorescent state (Rh) and releasing NO upon irradiation with UV or Vis light. Reproduced with permission [59]. Copyright 2025, John Wiley and Sons. (iv) A pH‐sensitive NO‐releasing nano producer (a), in combination with photodynamic therapy, to effectively eradicate bacterial biofilms and reduce associated health risks (b). Reproduced with permission [60]. Copyright 2024, John Wiley and sons.

The long‐recognized challenge in harnessing NO for therapeutic benefit lies in its fleeting nature and powerful, often dose‐dependent, biological activities. Despite its immense therapeutic potential, the clinical application of NO is hampered by its short half‐life and its gaseous nature, making its targeted delivery a significant challenge. A primary strategy involves the use of NO donors and carriers (discussed in detail elsewhere) [11]. These range from established clinical drugs such as organic nitrates (e.g., nitroglycerin for angina) and sodium nitroprusside (for hypertensive crises) to several classes of compounds, including S‐nitrosothiols (RSNOs) and diazeniumdiolates (NONOates). NO carriers such as nanoparticles (NPs), scaffolds, short nanofibers, and microspheres can protect the NO donor from premature degradation, improve its solubility, and, most importantly, be engineered to target specific tissues or cells [11]. The principal approaches for delivering NO in biomedical applications include: (i) gaseous NO inhalation, (ii) low molecular weight donors, (iii) polymeric and macromolecular carriers, (iv) cell‐ and tissue‐based delivery, (v) catalytic generation, (vi) prodrug‐based approaches, and (vii) inorganic and hybrid carriers (Table 1) [19]. The past several years have witnessed a surge in the development of sophisticated NO donors and carriers, moving beyond simple, spontaneous release to precisely controlled and targeted delivery systems. A study developed ceria NPs capable of decomposing S‐nitrosoglutathione (GSNO) to release NO while retaining their crystalline structure for at least four weeks (Figure 1A) [20]. A subsequent study was designed to create peroxynitrite (ONOO–) free NO‐embedded NPs (OFENs) that enable controlled NO release without generating ONOO^–^ [21]. In this system, NO was first sequestered within the pores of a Prussian blue lattice, after which the framework was cloaked with the antioxidant biliverdin to form the final OFENs construct (Figure 1B). The OFENs released NO at a concentration of approximately 1.8 µm, with sustained release lasting up to 7 days. Such advances highlight how next‐generation platforms may unlock the full therapeutic potential of NO. For instance, catalytic approaches leverage inorganic or organometallic catalysts to convert abundant endogenous substrates (e.g., nitrite, RSNOs) or prodrugs into NO in situ, offering potentially unlimited NO payloads, precise spatiotemporal control, and tunable activation triggers. Catalytic strategies primarily fall into two main categories: biomimetic systems that mimic natural enzyme function and advanced catalytic nanomaterials, or “nanozymes,” that produce NO in response to specific triggers. The biomimetic approach aims to replicate the function of the body's NO synthase (NOS) enzymes, which naturally produce NO from the amino acid _L_‐arginine (l‐Arg). In synthetic systems, a common strategy is to use metal‐based catalysts (e.g., Cu^2+^, Fe^3+^, Au, Ce) or nanozymes to decompose endogenous NO carriers, such as RSNOs, which are present in biological fluids like blood [4, 22, 23]. A well‐established example is copper (II) ion‐mediated NO generation, where the thiolate anion reduces cupric ions (Cu^2+^) via redox‐shuttle mechanism to cuprous ions (Cu^+^), which subsequently catalyze the decomposition of RSNOs to release NO [24]. Copper‐containing NPs can be incorporated into materials and medical device coatings, where they catalyze NO release from RSNOs in the bloodstream directly at the material surface [22, 24]. Copper (II)‐ligand complexes typically exhibit K_cat_ values in the 10^1^–10^3^ s^−1^ range and can generate NO fluxes, reaching approximately ∼ 6–12 × 10^−10^ mol.cm^−2^min^−1^ [25, 26, 27]. Fe_3_O_4_ and Cu_2_O NPs mimic nitrite reductase, producing steady NO fluxes in mildly acidic (pH ∼ 6.5–7.0) microenvironments such as inflamed tissues [22, 28]. One study showed that Fe‐Cu complexes can facilitate copper redox cycling to generate NO (∼125 nm in 12.5 min), which can act as a non‐toxic nitrifying biofilm inhibitor [29]. At 1 mg loading, the Fe complex generated ∼25 nm NO via acid‐driven nitrous acid decomposition, while the Cu complex produced higher NO levels (∼50 nm) due to more efficient copper‐mediated catalysis. In contrast, the mixed Fe‐Cu system (0.5 + 0.5 mg) showed a pronounced synergistic effect, yielding a rapid NO burst of ∼125 nm within ∼750 s, followed by a gradual decline. This NO generation is dependent on the presence of nitrite and is effectively eliminated by NO scavengers, confirming the role of NO in the observed antibiofilm effect [29]. Another study developed cellulose nanofibril (CNF) hydrogels cross‐linked with Ca^2+^ to control in situ NO release from GSNO. Increasing CNF content (0.3–1.0 wt.%) reduced NO release from 1.61 to 0.40 mmol·L^−1^·h^−1^ due to a smaller mesh size, allowing sustained NO release over 16 h (Figure 1C) [30]. In nanozyme‐based in situ generation systems, nanomaterials with intrinsic enzyme‐like activity are used [13]. These nanozymes can catalyze the conversion of simple, stable precursors, most notably inorganic nitrite (NO_2_^−^) or RSNO, into NO [31]. This strategy is particularly powerful because nitrite is an abundant anion in the body, and its conversion to NO is highly favored under the specific acidic and hypoxic (low oxygen) conditions [28, 31]. Zinc oxide (ZnO) nanozymes catalyze NO release from endogenous RSNO via surface Lewis acidic Zn^2+^ sites that promote S‐N bond homolysis. This non‐redox mechanism yields lower but more predictable NO generation rates with minimal catalytic turnover [32, 33]. ZnO nanozymes harness their intrinsic glutathione peroxidase‐like and glycosidase‐like activities to catalytically cleave both endogenous GSNO and exogenous β‐galactoside‐masked NONOates, enabling sustained, enzyme‐free NO generation under physiological conditions. Yang et al. prepared ZnO particles that exhibited efficient NO release, generating 2.8 × 10^−5^ m NO from 1.0 × 10^−4^ m β‐gal‐NONOate, while preserving their catalytic property for 6 months [22]. Likewise, ceria NPs (CeO_2_ NPs) can efficiently scavenge NO radicals through redox cycling between surface Ce^3+^ and Ce^4+^ states. Several reports have shown that ceria NPs modulate NO in a dose‐dependent manner [20]. In vivo studies, most notably those conducted in embryonic zebrafish, have shown that at low NP concentrations, these materials act as inorganic antioxidants and reduce physiological NO levels, whereas at higher concentrations, they paradoxically elevate NO within the gut lumen, leading to increased intestinal NO [34]. Later, a study found that the l‐Arg‐loaded mesoporous hollow cerium oxide (AhCeO)‐Gel hydrogel protected neural stem cells (NSCs) from oxidative damage and continuously delivered NO, leading to promising results in spinal cord injury (SCI) repair [14].

**TABLE 1:** Summary of major NO delivery/generation platforms with quantitative NO flux and performance characteristics.

NO delivery/generation approach	Representative NO donors/materials	Trigger/ activation modality	Representative NO flux or release ranges	Controllability	Stability considerations	Key safety considerations	Ref.
Spontaneous decomposition donors (NONOates)	DEA/NO, DETA/NO, DPTA/NO, PEI‐NONOate	Spontaneous hydrolysis (pH, temperature dependent)	≥ 4 × 10^−10^ mol cm^−2^ min^−1^ from diazeniumdiolated silicone coatings Enzyme‐prodrug NONOate systems: 0.05–0.4 nmol cm^−2^ min^−1^	Moderate – tunable via donor structure, amine precursor, and environment	Moderate stability; release rate depends strongly on temperature, pH, and donor chemistry	Risk of burst release leading to hypotension, oxidative stress if uncontrolled	[71, 211, 436, 437]
S‐nitrosothiol (RSNO) donors (SNAP, GSNO)	SNAP‐PDMS, GSNO hydrogels, RSNO‐polymers	Thermal decomposition, light, transition metals (Cu^+^, Fe^2+^), thiols	0.5–4 × 10^−10^ mol cm^−2^ min^−1^ (physiological endothelial range) SNAP‐MWCNT graft: 0.5–4 × 10^−10^ mol cm^−2^ min^−1^ for 18 days GSNO films: ∼0.64 × 10^−10^ mol cm^−2^ min^−1^ sustained release	Moderate; external stimuli (light, metal ions) enable partial on‐demand release	Moderate stability but sensitive to heat, light, and metal ions; improved stability when immobilized in polymers	Excess NO release may induce cytotoxicity; metal catalysts may cause oxidative damage	[438, 439, 440]
Catalytic NO generation from endogenous RSNOs	Cu‐ or Se‐modified polymers, organoselenium catalysts	Catalytic decomposition of endogenous GSNO/RSNO	GSNO polymer + Cu catalyst: 1.1–10.3 × 10^−10^ mol cm^−2^ min^−1^ Cu‐SNAP polymer films: up to 12.4 × 10^−10^ mol cm^−2^ min^−1^ Ti–Cu interfaces: 7.3 × 10^−10^ mol cm^−2^ min^−1^	High, dependent on catalyst density and endogenous donor availability	High durability since NO is generated catalytically without donor depletion	Metal ion leaching toxicity (Cu, Se); ROS generation risk	[25, 26]
Enzyme‐prodrug NO generation systems	β‐Gal‐NONOate, glycosidase‐activated prodrugs, Cel2‐NO	Enzyme‐specific activation (localized enzyme presence)	Physiological flux: 0.05–0.4 nmol cm^−2^ min^−1^ (matches endothelial levels)	Very high, excellent spatial control (enzyme localization)	High stability of inactive prodrug until enzymatic activation	Risk of immune reaction to the enzyme; off‐target activation is possible	[71, 73]
Nanoparticle‐based NO delivery systems	GSNO‐PLGA nanoparticles, liposomes, MOFs	Passive degradation, diffusion, or triggered release (pH, enzymes)	Sustained release over 11.27 h from GSNO‐PLGA nanoparticles Microparticle systems: steady release 24–68 nM mg^−1^ over 7 days (Cu)‐MOF (HKUST‐1: ∼1.74 nmol L^−1^ h^−1^ for >14 days	High tunable by material composition, degradation kinetics	High stability due to encapsulation protection from degradation	Nanotoxicity risk, accumulation, and clearance concerns	[11, 80, 438]
Polymeric NO‐releasing biomaterials (covalent donor incorporation)	Diazeniumdiolate‐modified silicone, SNAP‐PDMS, sol–gel xerogels	Temperature‐driven decomposition or controlled diffusion	≥ 4 × 10^−10^ mol cm^−2^ min^−1^ sustained release for ≥20 days SNAP‐PDMS catheters maintain physiological flux >0.7 × 10^−10^ mol cm^−2^ min^−1^ for >30 days	Moderate–high via polymer structure engineering	Excellent stability when covalently linked or embedded in hydrophobic polymers	Minimal donor leaching; generally safe if a stable polymer matrix is used	[11, 211, 436]
Stimuli‐responsive NO release (light, NIR, heat, redox)	Photolabile RSNO, metal‐catalyzed NO donors, plasmonic nanoparticles	External triggers: light (UV, visible, NIR), heat, redox environment	Flux depends on stimulus intensity; rapid burst release is possible via photolysis or catalytic activation	Very high–precise spatial and temporal control	Lower stability; highly sensitive to environment and stimulus exposure	Phototoxicity, overheating, and excessive localized NO levels	[44, 47, 55, 438]
Endogenous enzymatic NO generation (NOS‐mimicking materials)	Organoselenium catalysts, NOS‐mimetic coatings	Catalytic conversion of endogenous substrates (RSNO, nitrite)	Physiological‐level sustained flux depending on catalytic efficiency (often endothelial‐like flux ranges)	High – long‐term continuous NO generation	Excellent long‐term stability due to catalytic mechanism	ROS formation, catalytic metal toxicity risk	[70, 441]

Another approach using bioinspired materials represents a paradigm shift in drug delivery, moving away from simple synthetic carriers to sophisticated, naturally derived systems that leverage the body's own biology to achieve more effective therapeutic outcomes [35, 36]. Several types of bioinspired delivery vehicles have been explored for diverse biomedical applications; however, in the context of NO generation or delivery, cells and related materials, such as stem cells, bacteria, and exosomes, have been the primary focus [35, 37]. Advances in rational gene editing and synthetic biology have enabled the development of programmed cells/bacteria as versatile living delivery vehicles for therapeutic agents, including NO. By engineering living cells or their secreted vesicles to produce NO on demand, these systems harness the inherent biological targeting, biocompatibility, and stimulus responsiveness of living cells. From prodrug‐activated stem cells and NO‐primed extracellular vehicles (EVs) to hybrid microneedle patches, such bioinspired platforms are advancing NO therapeutics toward greater precision and translational potential [35, 38]. Huang et al. reported an interesting approach to generate NO from genetically engineered human mesenchymal stem cells (MSCs) [39]. These MSCs were lentivirally transduced to express mutant galactosidase (β‐GAL^H363A^). When the tailor‐made NO prodrug (6‐methyl‐galactose‐benzyl‐oxy NONOate) (MGP) was administered, β‐GAL^H363A^ cleaved the galactose trigger, liberating 1 nmol NO in a spatiotemporally controlled manner (Figure 1D). Similarly, in another fascinating approach, researchers developed a biohybrid living system by combining bacteria such as *Escherichia Coli* (*E. Coli*) with NO functionalized materials [40, 41]. Chen et al. designed a biohybrid bacterial living system by conjugating tumor necrosis factor‐related apoptosis‐inducing ligand (TRAIL)‐expressing genetically programmed *E. Coli* strain MG1655 and black phosphate (BP) NPs to induce photo‐controllable NO production [41]. When irradiated with laser light, the BP NPs generate photoelectrons that are transferred to the bacteria, triggering the reductive metabolism of nitrate to produce NO in situ. Although these strategies have paved the way to generate NO on demand, directly at the disease site but challenges of safety, more controllable living delivery systems, and immunogenic response need to be further explored. In recent years, the use of cell‐based vehicles (such as exosomes) engineered for NO production has garnered substantial attention. Exosomes can be isolated from cells cultured in NO‐rich conditions, “priming” them with S‐nitrosylated cargo and endowing them with NO‐releasing capacity [42]. These NO‐loaded EVs retain targeting ligands from the parent cells and can deliver NO alongside endogenous miRNAs or proteins. Liu et al. developed a detachable microneedle array for the delivery of exosomes engineered with an NO nanomotor to promote the healing of Achilles tendinopathy [38]. In this study, exosomes collected from Achilles tendon stem cells were coated with a layer of 2‐methacryloyloxyethyl phosphorylcholine (MPC) and N, N′‐bis(acryloyl) cystamine (BAC) (MBA). These polymers form a high‐molecular‐weight network around the exosomes via electrostatic interactions, creating a stable core for the exosomes/MBA particle, which was then surface‐functionalized with _L_‐Arg (a NO precursor). Upon release from the microneedles, endogenous reactive oxygen species (ROS) and NOS converted the _L_‐Arg into NO. NO acts as a driving force, propelling the exosomes further into the damaged tendon tissue [38].

### 2.1 NO Photodonors

NO photodonor (NOPD) compounds are specialized compounds designed to release NO in a controlled manner upon exposure to light. This offers significant advantages over traditional NO donors, particularly in biological and therapeutic applications where precise spatial and temporal control over NO release is critical [16, 43]. NOPDs are typically composed of a molecule that binds to or contains a photosensitive group (a “photocage”) that, upon light irradiation at a specific wavelength, undergoes a chemical reaction leading to the release of NO through photoinduced rearrangement and photoinduced electron transfer [44, 45]. For medical NOPDs, the excitation wavelength must be carefully selected based on safety and penetration. UV light (200–400 nm) should be avoided due to carcinogenic risk and poor tissue penetration. Instead, excitation within the therapeutic optical window (≈650–1100 nm) is preferred, as this range minimizes absorption by hemoglobin and water while maximizing penetration depth. Visible (Vis) light (400–750 nm) is suitable for superficial and cutaneous applications, whereas near‐infrared (NIR) wavelengths (≥750 nm) are required for activation in deeper tissues [16, 46]. Transition metal complexes and organic chromophoric motifs have both been explored for the development of NOPDs over the past two decades. While both classes of NOPDs have advantages and drawbacks, transition metal complexes generally exhibit superior absorption properties, particularly in the Vis region, and better photochemical performances compared to their organic counterparts [47, 48]. Single‐photon excitation of NOPDs typically works only with Vis light, as NIR photons lack sufficient energy for NO release. This confines Vis‐light‐activated NOPDs mainly to shallow tissue applications. The quantum yield (*φNO*) values range from 0.0008 to 2.02, with N‐nitrosoamines showing high yields (1.6–2.02) and electron transfer systems typically lower (0.001–0.01) [49]. One way to bypass this is to engineer NOPDs with large two‐photon absorption cross‐sections, enabling femtosecond laser‐driven two‐photon excitation (TPE) in the NIR to excite the donor and trigger NO release deep within tissues. For NOPDs lacking intrinsic NIR two‐photon absorption (TPA), an alternative is to attach antenna chromophores either covalently via a spacer or positioned nearby noncovalently that have strong TPE cross‐sections. Upon NIR excitation, these chromophores transfer energy efficiently to the NOPD, triggering NO release (Figure 1E(i)) [16]. The TPA cross section of Fluor‐RSE at 800 nm was as δ = 63 ± 7 GM (Goeppert‐Mayer units), confirming its capacity for nonlinear excitation under NIR femtosecond irradiation. This value exceeds the typical TPA cross sections (<10–30 GM) of many small organic chromophores at similar wavelengths and is comparable to engineered two‐photon chromophores used in deep‐tissue imaging (e.g., Fluor‐RSE), as well as to conventional fluorescein (∼32–36 GM) under similar conditions [50]. The comprehensive details of the photophysical properties of NOPDs are beyond the scope of this work and have been discussed in other reviews [49, 51].

In recent years, research has advanced toward using nano‐ or micromotors for NO release, offering innovative dual functionality by combining biological effects with locomotion and propulsion [41, 52, 53]. The propulsion mechanism primarily relies on the generation of bubbles from the released NO, leading to a recoil force that propels the micromotor [52, 54]. Zhang et al. fabricated light‐armed NO‐releasing micromotors (LaNOrMs) using a programmable optical manipulation system for in vivo applications [21]. These micromotors, typically microparticles containing NO donors, are maneuvered and activated using light beams for precise control over their movement and NO release in the body. The microparticles were built around energy‐looping NPs (ELNPs) composed of lanthanide‐doped rare‐earth nanocrystals as the core, coupled with the NO precursor S‐nitroso‐N‐acetylpenicillamine (SNAP) using the amphiphilic polymer 1,2‐distearoyl‐sn‐glycero‐3‐phosphoethanolamine (DSPE)‐polyethylene glycol (PEG)‐NH_2_ (Figure 1E(ii)) [55]. Significant progress in the targeted NO release and real‐time monitoring of NO or drugs using light irradiation has opened new avenues for therapeutic and diagnostic applications [56, 57, 58]. For instance, Liu et al. introduced rhodamine‐NO precursor‐antibody (RhNO‐Ab), a dual‐function platform designed to improve primary open‐angle glaucoma (POAG) management through precise, tissue‐specific NO release and real‐time monitoring [59]. Upon Vis‐light irradiation, RhNO‐Ab liberates NO from its N‐nitroso group while undergoing a spirolactam‐to‐Rhodamine conversion, switching from non‐fluorescent to fluorescent for direct visualization (Figure 1E(iii)) [59]. Conjugation with ABCA1 antibodies enables targeted ocular delivery and tracking of its biodistribution. In another interesting study, Wang et al. fabricated a nanoproducer consisting of a Roussin's red salt ester (RSE) (NO donor) covalently conjugated to the photosensitizer protoporphyrin (PpIX) to obtain PpIX‐RSE [60]. This design enables the simultaneous generation of ROS and NO upon exposure to NIR light. The delivery vehicle is based on micelles formed from a PEG‐poly(β‐amino ester) (PBAE)‐PpIX copolymer, where PBAE provides tertiary amine groups capable of protonation, imparting charge reversal in the mildly acidic biofilm microenvironment (Figure 1E(iv‐a)). The PpIX‐RSE is incorporated into these micelles via π–π stacking interactions between PpIX moieties, ensuring efficient loading and co‐delivery of ROS and NO for synergistic antibacterial effects (Figure 1E(iv‐b)) [60]. Unlike conventional NO‐photodynamic therapy (PDT) platforms that typically co‐load separate NO donors and photosensitizers, often leading to premature release, low donor stability, and uncontrolled NO/ROS ratios, this covalent integration ensures synchronized photo‐triggered release, improved donor stability, and predictable therapeutic dosing. NOPDs represent an exciting area of research, offering a powerful tool for localized and controlled NO delivery with a wide range of potential therapeutic applications. Further advancements in this field hold the promise of revolutionizing treatment strategies for various diseases.

### 2.2 NO Prodrug

**FIGURE 2:**
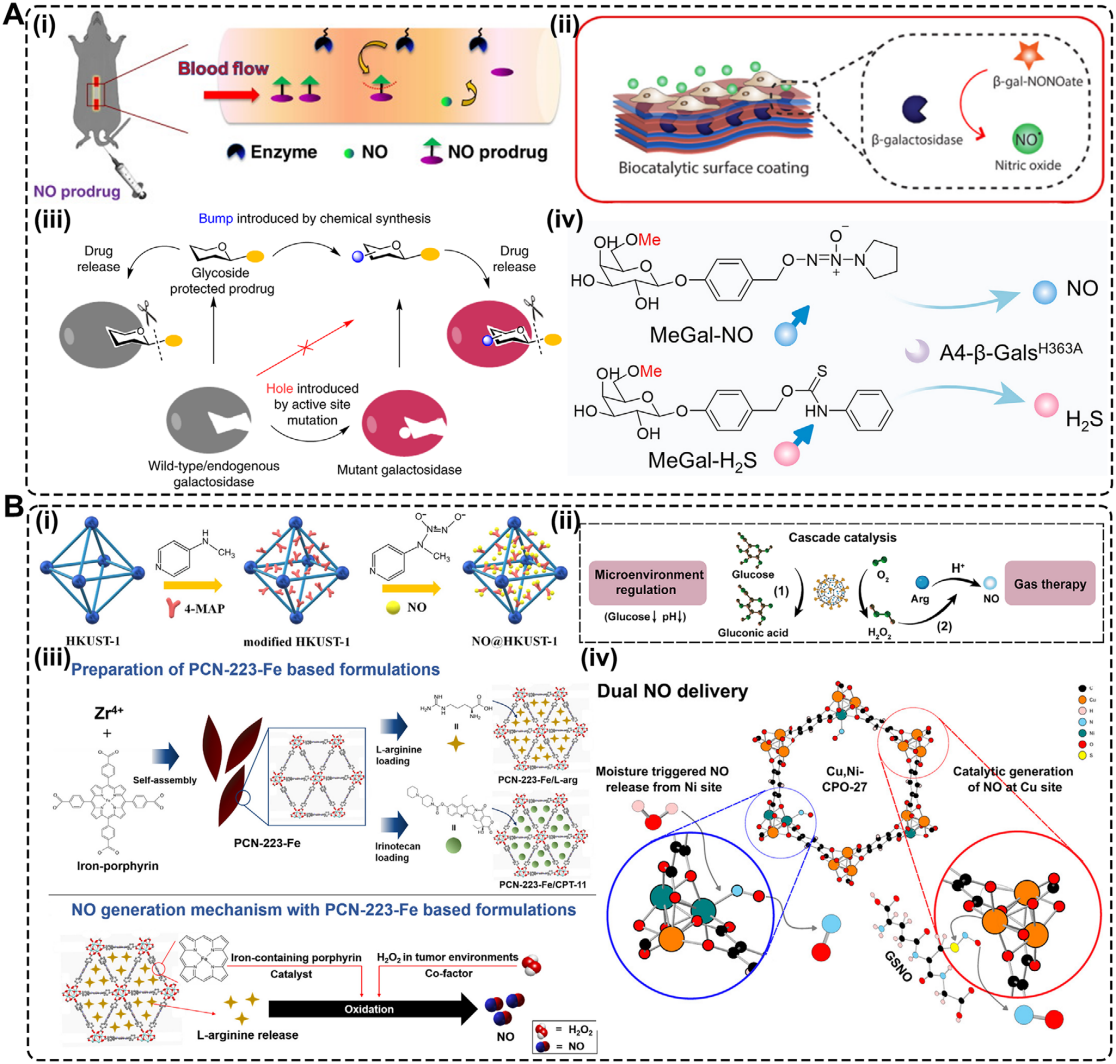
NO release from prodrugs and metal–organic frameworks (MOFs). (A) (i) Illustration for the enzyme immobilized on the vascular grafts to catalyze the decomposition of exogenously administered NO‐prodrug to release NO. Reproduced with permission [69]. Copyright 2015, Elsevier. (ii) Enzymatic synthesis of NO is engineered in this work into multilayered polyelectrolyte coatings. Adapted under the Creative Commons License CC‐BY [71]. Copyright 2018, American Chemical Society. (iii) Schematic illustration of the “bump‐and‐hole”‐based enzyme (β‐Gal) ‐ prodrug (galactosyl drug) system. Reproduced with permission [72]. Copyright 2025, Springer Nature. (iv) Schematics show NO/H_2_S codelivery system with the aid of engineered A4‐β‐Gals^H363A^ and MeGal‐protected prodrugs (MeGal‐NO and MeGal‐H_2_S). Adapted under Creative Commons License [74]. Copyright 2025, Elsevier. (B) (i) Schematic Illustration of the preparation of NO@HKUST‐1. Reproduced with permission [80]. Copyright 2020, American Chemical Society. (ii) Cascade reaction principle of AZG‐Gel: (1) Glucose was catalyzed by GOx to produce gluconic acid and H_2_O_2_; and (2) H_2_O_2_ catalyzed Arg to produce NO. Reproduced with permission [81]. Copyright 2024, Elsevier. (iii) Fabrication of PCN‐223‐Fe/L‐arg and PCN‐223‐Fe/CPT‐11 and mechanism of NO generation from PCN‐223‐Fe/L‐arg. Reproduced with permission[82]. Copyright 2023, Elsevier. (iv) Schematic showing the dual NO delivery mechanisms that can be performed by Cu/Ni‐MOF‐74. Adapted under the Creative Commons License CC‐BY 4.0 [84]. Copyright 2025, American Chemical Society.

NO prodrugs are inactive or low‐activity molecules that release NO in a controlled manner upon enzymatic, chemical, and photoactivation [17, 61, 62, 63]. They are designed to improve stability, bioavailability, and site‐specific NO delivery, overcoming limitations of direct NO donors like instability, rapid diffusion, and systemic side effects [64, 65, 66]. Performance of enzyme prodrug therapy (EPT) necessitates that the enzyme remains active throughout the time frame of the envisioned therapeutic application [61, 67]. Further, by controlling the concentration of the prodrug, researchers can tailor the amount of NO produced, leading to a personalized physiological response [68]. One such approach involved enzyme‐functionalized vascular grafts, designed to catalytically generate NO in situ by converting exogenously administered NO prodrugs into active NO [69]. In this study, immobilization of β‐Gal was carried out on poly(ε‐caprolactone) (PCL) based vascular grafts to which glycosylated NO prodrug contacted once administered via the tail vein. The immobilized enzyme facilitates the release of NO (∼2 µm) directly at the graft site with effective catalytic properties and preserves enzyme activity for 30 days (Figure 2A(i)) [69]. Yang et al. engineered an NO‐catalytic bioactive implant by covalently immobilizing organoselenium compounds onto the surface of a 316L stainless‐steel stent [70]. In the presence of endogenous NO donors, the modified stent generated a sustained NO flux of approximately 0.3 nmol·min^−1^·cm^−2^ over an extended period of up to 60 days, demonstrating long‐term catalytic stability and therapeutic relevance. The catalytic activity showed two‐phase kinetics (burst followed by sustained release) with a steady‐state NO release rate around 3 × 10^−10^ mol·cm^−2^·min^−1^. In another study, Winther et al. developed biocatalytic surface coatings for site‐specific NO release using substrate‐mediated enzyme prodrug therapy [71]. The approach involved forming a thin film by multilayered surface coatings of β‐Gal on metal wires. The optimized formulation enabled localized NO generation (∼100 pmol·cm^−2^·min^−1^) by adding the prodrug β‐Gal‐NONOate (20 µm), which releases NO upon enzymatic conversion (Figure 2A(ii)). The resulting coatings produced a physiologically relevant NO flux comparable to that of healthy human endothelium, supporting endothelial function and vascular homeostasis. Later, to further improve site specificity and precise NO delivery, Hou et al. used a bump‐and‐hole approach that leverages enzyme engineering and prodrug design to achieve highly specific and localized therapeutic effects [72]. The bump‐and‐hole approach was employed to modify the β‐Gal‐galactosyl‐NONOate, an enzyme‐prodrug pair, for efficient transport and delivery of NO at the targeted tissue. The design strategy centered on introducing a “bump” by methylating β‐Gal‐NONOate at the O6 position, yielding MeGal‐NO. This steric modification prevents recognition and activation by native, non‐engineered enzymes in the body, thereby reducing systemic side effects. Complementing this, a “hole” was created by engineering a subtle artificial cavity within the active site of β‐Gal (A4‐β‐Gal^H363A^) (Figure 2A(iii)). This cavity precisely accommodates the 6‐methyl “bump,” enhancing substrate–enzyme selectivity, improving prodrug stability in circulation, and enabling highly targeted, site‐specific NO release. Recently, the authors demonstrated another prodrug design, cellobioside‐NONOate (Cel2‐NO), and a unique enzyme, endocellulase (Cel5A‐h38), sourced from the uncultured bacterium found in the rumen of Hu sheep [73]. The key innovation of this system lies in its nearly complete orthogonality, meaning the Cel2‐NO prodrug remains stable and inactivated in the presence of the body's natural enzymes. This eliminates concerns about off‐target NO release and the associated side effects. In another study, Deng et al. developed a codelivery system utilizing an enzyme‐prodrug system for the targeted release of NO and H_2_S [74]. In this work, fabrication involved designing two methyl‐galactoside–protected prodrugs: MeGal‐NO and MeGal‐H_2_S (H_2_S donor). Both prodrugs were synthesized by chemically linking their respective gas‐releasing moieties to a 6‐O‐methyl‐β‐galactoside group, creating a “bump” structure that prevents activation by native β‐Gal. The engineered enzyme A4‐β‐Gals^H363A^, containing a complementary “hole” mutation in its active site, was immobilized on the desired substrate or surface (Figure 2A(iv)). Upon localization, the enzyme selectively cleaves the methyl‐galactoside moiety from the respective prodrug, triggering simultaneous and site‐specific release of NO (39.02 µm after 150 min) and H_2_S (23.18 µm).

Overall, enzyme‐prodrug therapy facilitates spatially and temporally controlled generation of NO at the target site, enabling precise modulation of local physiological responses. By restricting NO production to the intended tissue, this strategy offers personalized therapeutic outcomes while minimizing systemic exposure and off‐target effects.

### 2.3 Metal‐Organic Framework‐Based NO Carriers

A metal‐organic framework (MOF) is a class of porous, crystalline materials constructed from metal ions or clusters and organic linker molecules [8, 14, 75]. They can be thought of as a type of coordination polymer with a repeating network structure that can extend in one, two, or three dimensions. This unique structure leads to several key features that make MOFs highly versatile and attractive for a wide range of applications [75, 76, 77]. MOFs are widely explored as heterogeneous catalysts to generate NO by decomposing RSNO, using either endogenous blood RSNOs (e.g., GSNO) or supplemented donor pools [18, 75]. The latest research demonstrates a significant paradigm shift: MOFs are no longer being designed as simple, passive storage vessels for NO. Instead, they are being fabricated as sophisticated, stimuli‐responsive, and multifunctional nano‐architectures that can generate and release NO with unprecedented control. They are unusually tunable hosts for NO therapeutics: their pore size, metal nodes, and linkers can be engineered to store and release preloaded NO, catalyze in situ NO generation from precursors (e.g., RSNOs or l‐Arg), or co‐deliver NO with other bioactive materials [1, 78, 79]. For example, Zhang et al. reframed the MOF as an active biomimetic catalyst rather than a passive carrier [80]. This approach used a robust NO‐loaded copper (Cu)‐MOF (HKUST‐1) as a solid NO reservoir, then confined MOF particles within the cores of PCL‐based electrospun core–shell fibers to throttle water access and reduce premature NO loss (Figure 2B(i)). The composite exhibited controlled and stable NO release (∼1.74 nmol L^−1^ h^−1^ for >14 days).

Moving beyond simple catalysis, another interesting strategy involved fabricating the MOF as an intelligent, multi‐step cascade nanoreactor that can sense its environment and initiate a series of therapeutic actions. One emerging approach explored the design of a glucose‐responsive material that converts the diabetic wound microenvironment itself into a trigger [81]. A Zn‐MOF loaded with _l_‐Arg (Arg@Zn‐MOF) is co‐assembled with glucose oxidase (GOx) inside a chondroitin‐sulfate/pluronic F127 hydrogel. _L_‐Arg is confined in the Zn‐MOF, and GOx is immobilized/entrapped in the same gel network. Elevated glucose drives GOx to consume O_2_ and generate H_2_O_2_, acidifying the milieu. This cascade both regulates microenvironment (↓pH, ↓glucose) and promotes _L_‐Arg to NO conversion within the composite, yielding on‐demand NO (Figure 2B(ii)) [81]. Synergistic therapies using multifunctional platforms have emerged as a potent strategy for various therapeutic applications. Leveraging the MOF's porous nature for drug loading and its framework for catalysis, distinct therapeutic functions for a synergistic effect can be created. Ji et al. incorporated a hard‐wire heme‐mimicking catalyst into the MOF backbone to replicate enzymatic NO generation [82]. The porous coordination network (PCN)‐223‐Fe (comprising Zr_6_ nodes and Fe‐porphyrin linkers) is loaded with _L_‐Arg, enabling the pores to supply the substrate while the Fe‐porphyrin sites catalyze its conversion to NO using endogenous H_2_O_2_ abundant in tumors (Figure 2B(iii)).

Fabricating a homogenous crystalline structure with a precise, repeating ratio of two or more different metals in the nodes is an advanced synthetic challenge that pushes the boundaries of MOF chemistry [83]. Most current NO delivery platforms rely on a single mechanism, such as GSNO degradation, to release or generate NO [84]. A more advanced strategy involves the presence of multifunctional groups or multiple metal centers in MOFs, which can enable two distinct NO release mechanisms from a single material, enabling a “programmed” release profile over time. MOFs offer the unique ability to incorporate a wide range of functional groups directly onto their organic linkers, enabling a uniform distribution of chemical functionalities throughout the framework. Unlike systems that form distinct domains of separate linkers, MOFs allow for the mixing of multiple functional groups within the same lattice [85].

For instance, the properties of MOF‐74 can be precisely tailored through reticular chemistry, offering remarkable compositional versatility. Wang et al. reported the incorporation of up to ten different metal ions into a single MOF‐74 framework [86]. This mixed‐metal strategy not only diversifies the coordination environment but also enables multiple catalytic pathways to operate simultaneously within a single structure. Such multifunctional behavior endows MOFs with enzyme‐like characteristics, enabling complex, synergistic reactions, including simultaneous redox activity, RSNO decomposition, and cofactor regeneration within a single material. This flexibility in MOF design was exploited in a recent work by Main et al., who developed a mixed‐metal MOF‐74 that combines two NO mechanisms within a single lattice [84]. Cu/Ni mixed‐metal MOF‐74 analogues are synthesized so that Ni sites bind and release pre‐adsorbed NO (fast “burst” release upon hydration), while Cu sites catalyze RSNOs (e.g., GSNO) decomposition to NO for a prolonged duration (Figure 2B(iv)). Ni^2+^ exhibits higher affinity for gaseous NO but low catalytic activity toward thiol cleavage, whereas Cu^2+^ efficiently mediates redox cycling with thiols to generate NO catalytically. This dual‐action behavior is intrinsic to the mixed‐node crystal rather than a blend of two materials, simplifying device integration and enabling sequential therapy. Together, these strategies shift MOF‐NO systems from simple gas storage to programmed, pathology‐responsive, and device‐ready therapeutics, with release profiles written directly into crystal chemistry and material architecture.

## 3 Biomedical Applications of NO

Over the past two decades, advancements in chemical engineering, materials science, and nanotechnology have facilitated the transformation of NO research from systemic pharmacology to precision biomedical engineering [87, 88]. Our earlier publication provided a comprehensive overview of NO in established biomedical applications, including wound healing, cardiovascular homeostasis, antibacterial, and tumor targeting functions [11]. Breakthroughs in next‐generation platforms can now enable the localized, sustained, and stimuli‐responsive release of NO to enhance biological effects in a spatiotemporal manner. These advances have propelled a new wave of biomedical applications that extend far beyond the traditional ones. In this article, we focus exclusively on these emerging avenues, not discussed in our previous review. These include NO applications in antiviral, ophthalmic, nerve regeneration, and immunomodulation. In the previous section of this article, we introduced stimulus‐responsive and theranostic systems that enable real‐time monitoring of NO alongside treatment, which illustrate a paradigm shift from conventional NO administration toward precision, on‐demand, and multifunctional biomedical applications with promising clinical translational potential.

### 3.1 Viral Infections

Viral infections are primarily classified into respiratory and non‐respiratory types. Respiratory viral infections are among the leading causes of illness and death worldwide, affecting people of all ages [89, 90]. These viruses mainly target the respiratory system, causing a range of diseases from common colds to severe conditions [90]. such as pneumonia and acute respiratory distress syndrome (ARDS). The most common respiratory viruses include influenza, respiratory syncytial virus (RSV), rhinovirus, and coronavirus [83]. Globally, these viral infections impose a significant disease burden, lead to economic losses, and strain healthcare systems. Besides the respiratory system, viral infections affect other organs, including the skin, gastrointestinal tract, liver, and nervous system, and leading to systemic and hemorrhagic conditions. These include hepatitis, human papillomavirus (HPV), herpes, rabies, human immunodeficiency virus (HIV), dengue, etc. Such non‐respiratory viral infections pose a major global challenge, resulting in both acute and chronic illnesses, long‐term complications, and high mortality rates [91, 92].

NO functions as a double‐edged sword in viral infections, exhibiting both antiviral and the potential to contribute to host tissue injury when dysregulated. In viral infection, inducible NOS (iNOS) is upregulated in immune cells such as macrophages, neutrophils, and dendritic cells, leading to localized production of high NO levels that can potentially restrict pathogen replication. It may also damage host tissues if prolonged [79, 93]. Hence, its activity highly depends on the site, concentration, and time of production, as well as the host‐virus interactions. Mechanistically, NO acts through multiple pathways: (i) direct chemical inhibition of viral proteins via S‐nitrosylation and metal center disruption (e.g., cysteine proteases, polymerases); (ii) genome damage through reactive nitrogen species such as peroxynitrite; and (iii) entry interference, with emerging evidence that NO donors can weaken spike‐angiotensin‐converting enzyme 2 (ACE2) interactions in coronaviruses [94, 95, 96]. Simultaneously, NO regulates innate and adaptive immune responses by modulating cytokine production, dendritic/T‐cell function, and vascular toning to either facilitate clearance or exacerbate immunopathology depending on dosage, location, and duration [97, 98, 99, 100, 101].

Translational medicine has utilized NO actively in three application streams. First, inhalation of NO has been used as a selective vasodilator with antiviral and anti‐inflammatory benefits in patients with severe coronavirus conditions [102]. Inhaled NO improves ventilation‐perfusion matching and oxygenation primarily by reversible vasodilation and modulation of pulmonary vascular tone, rather than by permanently reversing vasoconstriction [103]. Furthermore, clinical studies have reported reduced need for ventilatory support and improved oxygenation in selected patient populations, although outcomes vary across dosing regimens and study designs [96]. Cigarette smoke has been reported to contain intermittent high concentrations of NO (250–1350 ppm); however, its biological effects are multifactorial, and NO should be regarded as one of several reactive components rather than the dominant antiviral agent [104]. While NO can diffuse across membranes and interact with viral particles, attributing antiviral effects of cigarette smoke primarily to NO oversimplifies its complex chemical composition. Encouraged by this study, Yu et al. developed a NO‐generating device capable of producing NO at concentrations exceeding 100 ppm while maintaining NO_2_ below 3 ppm [105]. With the onset of coronavirus disease (COVID‐19), numerous studies have emerged since the initial development of NO‐generating devices that could be used as therapies for COVID‐19. Reports from a randomized trial showed that NO inhalation at 160 ppm for 6 h in COVID patients increased the duration of oxygen‐free days, reduced ventilatory support, led to shorter hospital stays, and improved clinical outcomes [106]. These observational and early interventional studies reported improvements in oxygenation at low to intermittent high doses, while randomized trials continue to evaluate progression and safety endpoints [93, 107, 108, 109, 110, 111, 112, 113]. Importantly, optimal dosing strategies, frequency, and duration of NO administration remain unresolved, and clinical implementation is limited by logistical requirements, cost, and adverse effects such as methemoglobinemia [104, 114, 115].

Second, NO‐releasing/ NO‐amplifying therapeutics have been engineered for targeted antiviral delivery, ranging from small‐molecule donors and enzyme/hypoxia‐activated prodrugs to biomaterial carriers that localize NO to the infected mucosa. NO‐amplifying strategies exploited endogenous pathways to enhance NO bioavailability in the airways. Synthetic NO donors exhibit distinct release kinetics and therefore differing antiviral efficacy. Comparative studies demonstrated that S‐nitroso‐N‐acetylpenicillamine (SNAP), a direct NO donor, inhibited SARS‐CoV‐1 replication at non‐toxic concentrations, whereas sodium nitroprusside (SNP), which requires reductive activation, showed limited antiviral activity [116, 117]. SNAP showed that NO generated by the action of iNOS inhibits SARS‐CoV replication and reduces viral protein and RNA synthesis [118]. A similar study on SNAP in SARS‐CoV‐2 infection revealed that it dysregulated viral replication in a dose‐dependent manner and reduced viral proteases by covalent binding of NO released from SNAP in vitro [119]. A study in a lung injury model of acute respiratory distress syndrome (ARDS) demonstrated that intratracheal administration of SNAP significantly improved respiration and reduced edema, unlocking the potential of NO donor administration in ARDS [120]. Another NO donor, GSNO, a stable airway reservoir of NO, was evaluated for therapeutic intervention in viral infections. GSNO demonstrated protective effects in lung injury models. In a study on porcine circovirus 2 (PCV2), GSNO released high levels of NO to inhibit viral replication in vitro and reduced the rate of infection in a mouse model [121]. Another study showed that inhibition of GSNO reductase (GSNOR) by N6022 prolongs GSNO half‐life, sustaining antiviral and anti‐inflammatory effects in autoimmune encephalomyelitis [122]. In contrast, recent reports suggest that GSNOR elicited an antiviral innate immune response by increasing interferon release, thereby inhibiting the replication of herpes simplex virus‐1 (HSV‐1) and vesicular stomatitis virus [123]. NVN1000, a NO donor belonging to NONOate class, has antiviral effects in HPV raft cultures [124]. Similarly, dietary nitrate/nitrite supplementation augments NO production via the nitrate‐nitrite‐NO pathway, an approach now under investigation for host‐directed therapy in respiratory viral infections [125]. A recent study examined the efficacy of CR‐0305 as an NO donor in SARS‐CoV‐2 [126]. It revealed that CR‐0305 binds to the catalytic site of the SARS‐CoV‐2 papain‐like protease, inhibiting viral replication and promoting the host innate immune response. In respiratory and non‐respiratory viral infections, reviews on NO donors converge on multi‐pronged benefits: partial suppression of replication, improved perfusion/ventilation matching, and tempering of thrombo‐inflammatory cascades that drive the disease progression [79, 127, 128]. Apart from NO donors, other compounds are documented to modulate iNOS activity in antiviral function, such as bilirubin upregulates NO release against HSV‐1 [129]. However, contrasting results were observed in the action of dihydropyrazole derivatives against vaccinia virus, as well as ribavirin and acetylsalicylic acid against hepatitis C virus, where NO production contributes to the viral pathophysiology [130, 131, 132].

**FIGURE 3:**
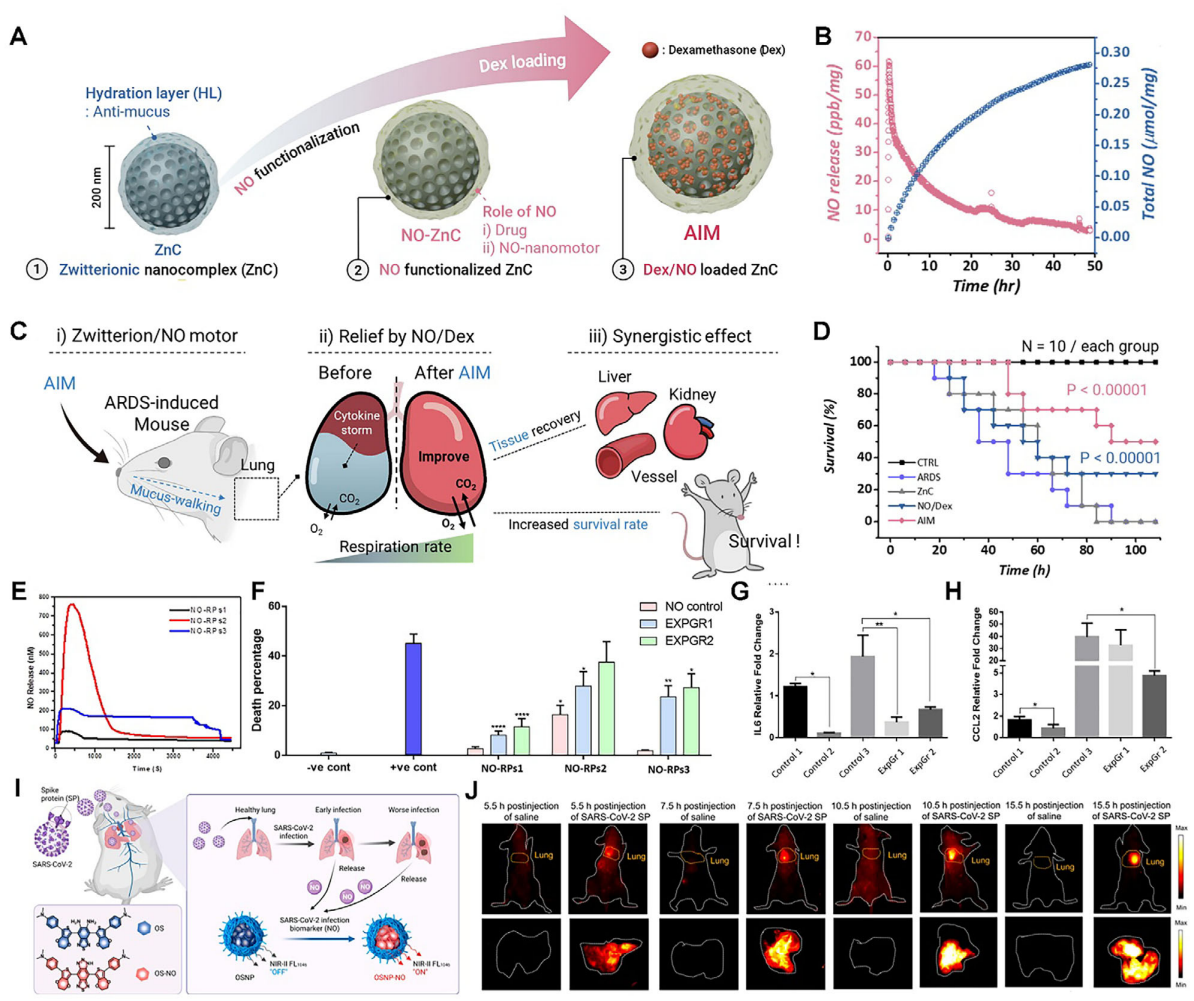
Antiviral function of NO. (A) Schematic illustration of an anti‐inflammatory mucus permeator (AIM). (B) The graph represents the NO released from AIM. (C) Schematic illustration of the action of AIM in comprehensive tissue recovery in ARDS‐induced mice. (D) Comparison of the survival rate between ARDS and AIM‐treated mice. Reproduced with permission.[142]. Copyright 2024, Elsevier. (E) NO release pharmacokinetics from the developed NO‐RPs nanoformulations using the NO electrode. (F) Cell death assessment in NO‐RPs under shear stress. Gene expression of inflammatory markers in NO‐RPs (G) IL‐6. (H). CCL2. Adapted under the Creative Commons Attribution (CC‐BY).[144] Copyright 2021, Frontiers. (I) Schematic representation of the mechanism of OSNP for in situ noninvasive observation of the changes in NO in SARS‐CoV‐2 infection in vivo. (J) NIR‐II fluorescence images at different timepoints after intratracheal injection of 1 mg kg^−1^ SARS‐CoV‐2 SP and 5 mg kg^−1^ OSNP (saline) into living mice. Reproduced with permission.[146] Copyright 2023, American Chemical Society.

Third, nanotechnology is opening precision avenues: NPs that generate or ferry NO (or synergize with NO biology) can concentrate antiviral activity at barrier surfaces, penetrate biofilm‐like mucus/bio interfaces, and co‐deliver antivirals or immunomodulators. Use of NPs has been shown to provide greater stability and controlled, sustained, and targeted release of NO, with fewer or no side effects [79, 115, 133]. Different NP formulations have been explored in numerous studies to combat various viral infections in the last decade. Incorporation of NO donors into medical‐grade polymers, polysaccharides, and macromolecular scaffolds enables controlled delivery of NO to targeted tissues or organs [33, 79, 133, 134, 135, 136, 137, 138, 139, 140]. A study on early‐stage COVID utilized inhalation of silver NPs to suppress infection in lower airways at a minimal inhibitory concentration, which further inhibited progression of the disease to the upper airways [141]. Newer nanocomplexes have been introduced to overcome the obstruction by mucus present in the respiratory tract. For this, a zwitterion‐functionalized multi‐drug nanocomplex (ZnC) loaded with dexamethasone was synthesized, referred to as the anti‐inflammatory mucus permeator (AIM) (Figure 3A) [142]. This nanomotor complex retains a hydration layer that facilitates ZnC delivery through the mucus layer of the respiratory tract and delivers it to alveoli, where it releases NO gas in ARDS treatment. Nearly 249.3 mmol/mg of NO was released in the alveoli in 48.76 h (Figure 3B). In an in vivo model of ARDS, inhalation of AIM increased anti‐inflammation, enhanced the functioning of organs such as the liver, kidney, and blood vessels, and promoted survival in mice (Figure 3C,D). Another study compared NO inhalation at 70 and 140 ppm and NO‐NPs (10 mg/mL) in a rodent model of ARDS [143]. NO‐NPs improved arterial oxygen levels (PO_2_) at a high fraction of inspired oxygen (FiO_2_)_,_ which is higher than that of NO inhalation at different concentrations. Furthermore, the circulatory and pulmonary interstitial neutrophil count was reduced in NO‐NPs than in their counterparts. On another NO delivery platform, NO‐releasing hydrogel nanocomposite‐based NPs (NO‐RPs) were developed with different compositions and tested in vitro under mechanical ventilation for NO release (Figure 3E) [144]. All three NO‐RPs showed varying cell viabilities (Figure 3F). Exposure of NO‐RPs to epithelial cells, followed by mechanical ventilation, reduced inflammation and cellular damage (Figure 3F–H). Apart from the traditional NP formulations for treatment, studies have ventured into vaccine adjuvant formulations, such as aluminum hydroxide [145]. Studies mainly focused on unlocking NO's therapeutic potential in antiviral infections. However, there is a lack of understanding of the onset of NO pathogenesis in viral infections. A recent study opened a new avenue of monitoring NO fluctuations in SARS‐CoV‐2 infections [146]. They developed an activatable NIR‐II fluorescent molecular nanoprobe (OSNP) that can penetrate deeper tissues with high resolution (Figure 3I). OSNP monitored NO levels in vivo during SARS‐CoV‐2 infection and detected elevated NO as early as 5.5 h post‐infection, concluding that NO levels correlate directly with the viral progression (Figure 3J). Such a study has the potential to unveil the underlying pathogenesis of viral infections and is beneficial in the optimization of therapeutic interventions. The field of medicine is shifting toward precisely controlled, mechanism‐based NO therapies that harness its antiviral and immune‐modulating effects while limiting tissue damage. Future efforts should prioritize targeted delivery, combination with existing antivirals, and rigorous clinical testing in both chronic and emerging infections.

### 3.2 Ophthalmic Treatment

NO has pivotal roles in ocular physiology and pathology. It regulates intraocular pressure (IOP), retinal blood flow, neurotransmission, and immune responses within the eye [147, 148, 149, 150, 151]. In ocular homeostasis, all three types of NOSs are expressed. nNOS is expressed in the inner and outer plexiform layer, inner retina, bipolar cells, nuclear layer (inner and outer), amacrine cells, limbus, cornea (endo‐ and epithelial cells), epithelial cells of the retinal pigment, lens epithelium, and ganglion cells [152, 153]. eNOS is present in photoreceptors, bipolar cells, ciliary bodies, trabecular network, Schlemm's canal, uveal vascular endothelium, Müller cells, amacrine cells, horizontal cells, and ganglion [154]. eNOS levels are elevated in retinal vessels and ganglion cells during ischemia/reperfusion injury (I/R) [148, 155]. iNOS is detected in the iris, ciliary bodies, and blood vessels after stimulation. Under inflammatory conditions, iNOS is expressed in the inner nuclear layer, macrophages, outer region of photoreceptors, and microglia [149, 150]. The biological action of NO is dose‐dependent in ocular function and disease. Elevated IOP, a hallmark of glaucoma, plays a critical role in disease progression [148, 156, 157, 158]. By regulating the trabecular framework, NO released by either eNOS or nNOS can lower IOP by regulating the aqueous humor outflow via changes in the ciliary muscle contraction [158, 159]. NO functions as a vasodilator as a result of the action of eNOS expressed in the vascular endothelium of retina and choroid to regulate the impaired blood flow to the optic nerve, which is most common in glaucoma [148, 158]. Conversely, NO dilates the intraocular and extraocular vessels to protect the optic nerve from ischemic damage during high IOP in glaucoma and reduces the outflow resistance of aqueous humor by dilating the episcleral vessels, thus decreasing IOP [160, 161, 162, 163]. Along with vascular dysfunction, neuroprotection is critical in glaucoma, as it can lead to visual field defects due to severe damage to the retinal nerve fibers and ganglion cells [164]. NO exhibits dose‐dependent neuroprotection effects, promoting neuronal cell survival at lower concentrations (<1 μμ) but inducing neuronal and ganglion cell death at higher levels (>50 μμ) [148, 157, 165, 166].

NO functions as a double‐edged sword, as it can aggravate or alleviate the pathology in retinal diseases. NO is reported to decrease the phagocytic function of retinal pigment epithelium (RPE), leading to accumulation of ROS debris between photoreceptors and RPE, thereby causing photoreceptor degradation [167]. Additionally, studies of ischemic injury suggested that iNOS expression promotes retinal apoptosis and damage to retinal ganglion and bipolar cells, which can be rescued by the addition of an iNOS inhibitor [168, 169, 170, 171]. Elevated NO was observed in the aqueous humor of patients with retinal vein occlusion and I/R injury [172, 173, 174]. High NO, along with superoxide, can form peroxynitrite, which can oxidize sulfhydryl groups to promote lipid peroxidation, further damaging the retina. However, peroxynitrite may contribute to vascular relaxation and inhibition of platelet aggregation [175, 176]. Experimental evidence from studies on diabetic retinopathy (DR) reveals that NO is highly synthesized by iNOS in DR, leading to oxidative/nitrosative stress, vascular leakage, and neovascularization [177, 178, 179]. The addition of NOS inhibitor L‐NAME can reverse DR in mice [180].

On the other hand, in the retinal microvasculature, NO produced by eNOS in retinal endothelial cells regulates the vasomotor function [152]. In an IOP model, retinal ischemia significantly increases vascular superoxide, thereby inhibiting NO‐mediated dilation of retinal arterioles [181]. NOS inhibitor,  N(gamma)‐nitro‐_L_‐Arg, administered before the onset of retinal ischemia, showed a delay in the reperfusion in rats [182]. In age‐related macular degeneration (AMD), NO acts as protector and perpetrator. Physiological NO levels from constitutive synthases (eNOS, nNOS) preserve choroidal perfusion and retinal cell function. Studies have revealed that eyes affected by AMD exhibit reduced expression of nNOS and eNOS in the retina, RPE, and choroidal vasculature [154]. Such a decline likely disrupts choroidal blood flow and homeostasis, contributing to retinal hypoxia and disease progression. Indeed, reduced foveolar choroidal blood flow is associated with an increased risk of choroidal neovascularization (CNV) in AMD, suggesting that decreased NO‐mediated vascular support may predispose to neovascular changes [183]. Under oxidative or inflammatory stress, RPE cells upregulate iNOS, leading to excessive NO production. NO then reacts with superoxide to form peroxynitrite (ONOO^−^), a potent oxidant that triggers protein nitration, mitochondrial dysfunction, nuclear factor kappa B (NF‐κB) activation, and apoptosis in RPE cells [184]. These events contribute to RPE degeneration, drusen accumulation, and progression of AMD [185].

NO has a crucial role in ocular wound healing. It can promote rapid antimicrobial action via nitrosative stress against biofilms on the cornea and perioperative surfaces [186, 187, 188, 189]. At physiological levels, NO promotes epithelial migration, organized collagen deposition, and reinnervation while modulating inflammation [190, 191]. Excessive NO drives keratocyte apoptosis and neovascularization [192, 193, 194]. In addition to these attributes, NO has antifibrotic activity, as its signaling can modulate fibrotic activity during optic wound healing [193]. Exogenously, it can decrease myofibroblast differentiation of human keratocytes and corneal opacity after injury in a murine model [191].

As a stem cell modulator, NO regulates limbal epithelial stem cells (LSCs) in a bidirectional manner [195]. At controlled levels, it enhances the proliferation, migration, and survival of LSCs, making it a potential therapeutic tool in corneal regeneration and LSCs deficiency [196]. However, dysregulated or excessive NO can impair stem cell function and promote disease progression [195]. Additionally, NO causes smooth muscle relaxation, which can be used to inhibit myopia progression in children [197]. Further, inhibition of NOS by N(gamma)‐monomethyl‐_L_‐Arg (L‐NMMA) alleviated choroidal fibrosis in guinea pigs [198].

**FIGURE 4:**
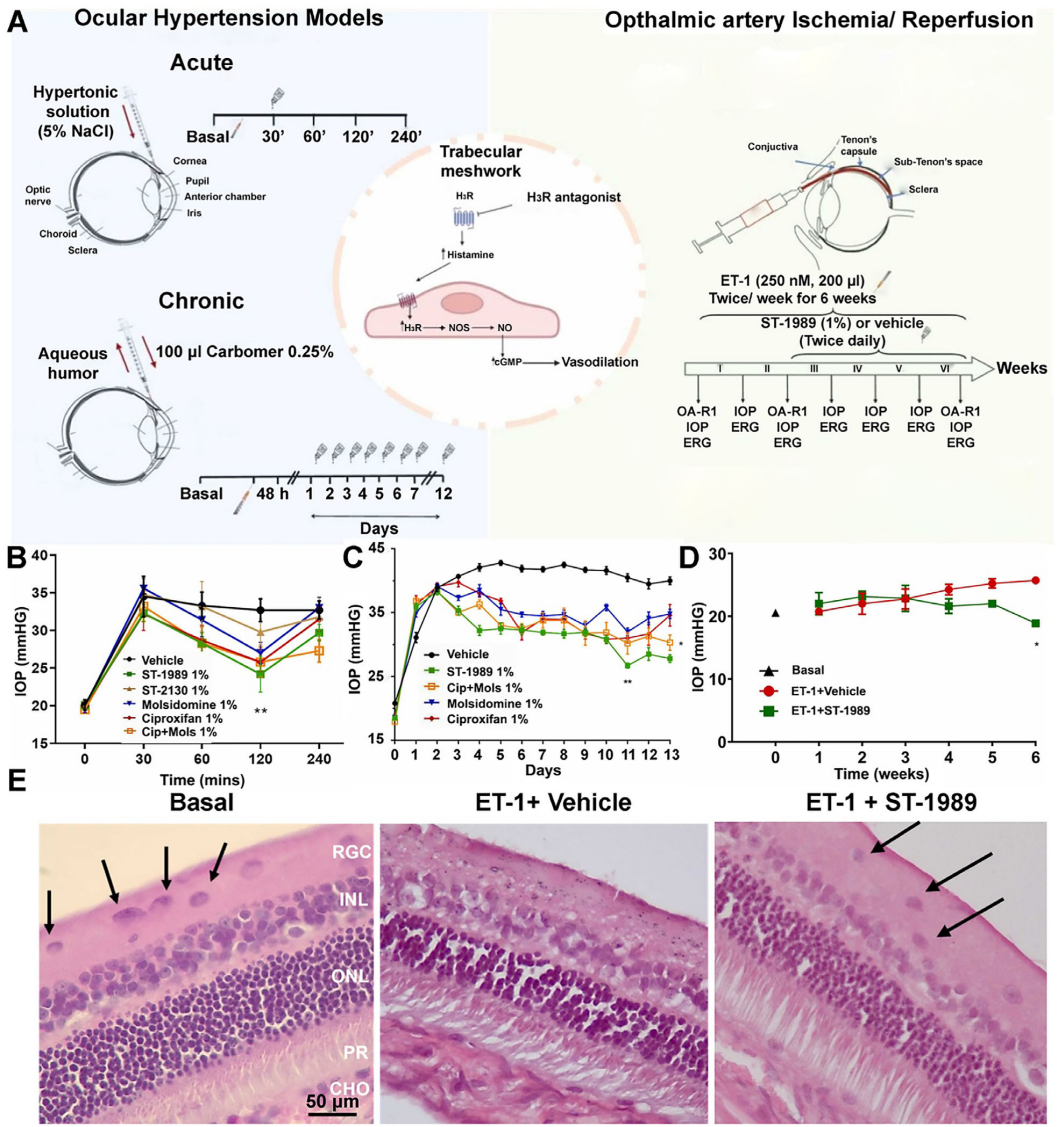
NO in ophthalmic treatment. (A) Schematic representation of the use of histamine H3 receptor antagonist/NO donor hybrid compounds in both acute and chronic in vivo models for treating glaucoma. (B, C) The graph illustrates the intraocular pressure (IOP) measured at various time points in (B) acute and (C) chronic ocular hypertension models. (D) IOP was measured under basal conditions and every week in a drug‐free state in a retinal artery I/R model, while eyes were treated daily with compounds from week three until the end of the experiment. (E) Representative H & E staining images of the retinal ganglion cell (RGC) layer after ET‐1‐induced I/R injury in eyes treated with ST‐1989 and vehicle. Black arrows indicate the RGCs. Adapted under the Creative Commons Attribution CC‐BY [209]. Copyright 2024, Elsevier.

Based on the therapeutic potential of NO, NO donors have made tremendous progress from experimental tools to clinical applications in ocular treatments. Topical administration of NO‐donating drugs such as nitroglycerin, isosorbide dinitrate, sodium nitroprusside, and sodium nitrite can lower IOP in glaucoma patients [199, 200, 201, 202, 203]. Conversely, prolonged use of these NO donors can increase ocular hypertension [202]. Hence, NO donors capable of releasing NO and other active compounds to synergistically lower IOP were explored. One such example is Latanoprostene bunod (LBN, Vyzulta), an NO‐donating prostaglandin analog approved by the U.S. Food and Drug Administration (FDA) for the treatment of glaucoma [204, 205]. LBN is hydrolyzed in the eye to yield latanoprost acid (LA) and butanediol mononitrate (BDMN). While LA binds to prostaglandin F receptor in the eye to upregulate matrix metalloproteinase (MMP) to degrade the extracellular matrix and promote uveoscleral outflow, BDMN breaks down into inactive 1,4‐butanediol, releasing NO [204]. Although LBN is widely tolerated, continuous administration is associated with risks, such as nitrate tolerance and increased formation of RSNOs resulting from the reaction between NO and protein dysregulation, which can lead to ocular hypertension and damage the conventional outflow [206]. Another widely used NO donor for glaucoma treatment is the topical administration of nipradilol, a non‐selective beta blocker that can reduce IOP in humans [207, 208]. In a recent study, researchers synthesized a histamine H3R antagonist‐NO donor hybrid compound, ST‐1989, and tested its efficacy in treating IOP (Figure 4A) [209]. In both acute and chronic models of ocular hypertension, ST‐1989 lowered IOP significantly compared to reference drugs, ciproxifan and molsidomine in short and long‐term treatments (Figure 4B,C). In an ophthalmic artery I/R model, ST‐1989 treatment mitigated endothelin‐1‐induced retinal degeneration, as evidenced by its IOP‐lowering profile (Figure 4D). Further, ST‐1989 preserved the retinal tissue as well as the ganglion cell layer from I/R damage (Figure 4E). As NO inhibits biofilm formation, NO donors such as sodium nitrite have been explored. Kim et al. demonstrated that sodium nitrite impeded biofilm formation by *Pseudomonas aeruginosa (P. aeruginosa)* and *Staphylococcus aureus* (*S. aureus*) on contact lens surfaces in a dose‐dependent manner [189]. Similar revelations were reported in the action of NO donors in *Acanthamoeba keratitis* [210].

**FIGURE 5:**
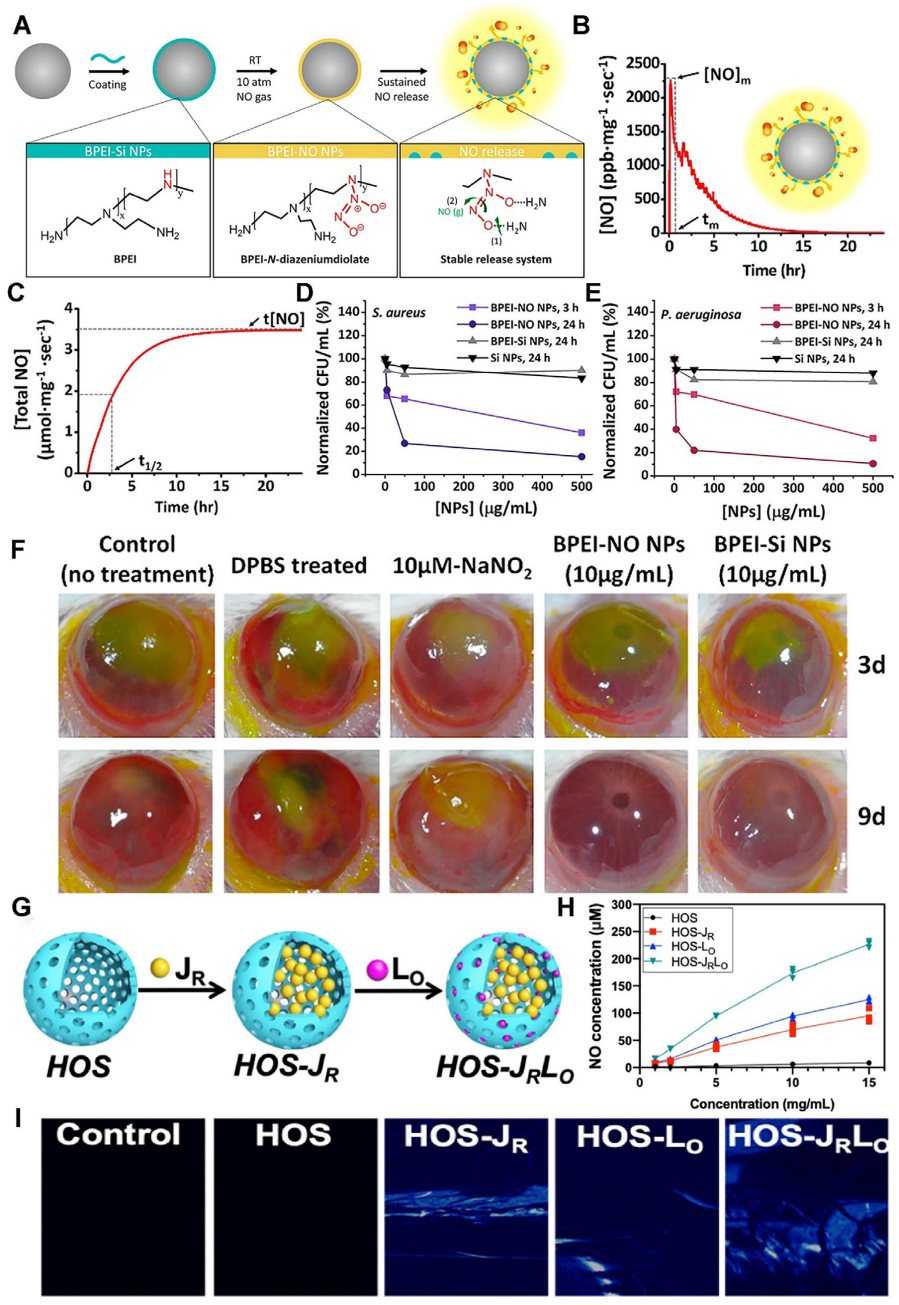
NO‐based interventions for ocular disorders. (A) Schematic illustration of the preparation of BPEI‐functionalized NO NPs and the mechanism of sustained NO release from NPs. (B, C) NO release profiles of BPEI‐NO NPs in PBS at 37°C: (B) real‐time concentration; and (C) accumulated amount of NO. (D, E) Bactericidal effects of BPEI‐NO NPs in comparison with Si NPs and BPEI‐Si NPs at different concentrations at 3 h and 24 h in a colony‐forming unit assay, inoculated with (D) *S. aureus* and (E) *P. aeruginosa*. (F) Images of mouse corneas with BPEI‐NO NP treatment vs. control at 3 days and 9 days after mechanical damage and infection with *P. aeruginosa*. Reproduced with permission [215]. Copyright 2018, American Chemical Society. (G) Schematic of co‐encapsulation of hydrophobic (JR) and hydrophilic (LO) into hybridized hollow‐structured MOS (HOS). (H) The graph shows NO production in different concentrations of HOS, HOS‐JR, HOS‐LO, and HOS‐JRLO in the presence of ascorbic acid and eNOS. (I) Fluorescence images of the NO released from HOS, HOS‐JR, HOS‐L0, and HOS‐JRLO in the anterior chamber of Cav1 knockout mice. Reproduced with permission.[216] Copyright 2021, Elsevier.

NO‐delivery platforms are crucial in eye treatment because they transform NO from a short‐lived, unstable molecule into a controllable therapeutic tool enabling precise targeting, sustained release, enhanced safety, and improved patient compliance. Recent advances in NO‐donating therapeutics and controlled delivery platforms highlight their emerging significance in ophthalmology. To stabilize and regulate NO availability in ocular tissues, NO donors encapsulated in NPs, micelles, dendrimers, hydrogels, and implantable devices are being explored [175, 211]. Polymersomes synthesized using light‐responsive N‐nitrosamine‐based monomer can release NO in response to UV or Vis light for enhanced corneal wound healing [212]. Another study designed a combined pH‐ and light‐responsive gatekeeper system composed of a pH‐jump reagent as an intermediate stimulus with calcium phosphate NPs acting as a shielding layer. The uncapping of the system is initiated by light irradiation, followed by lowering pH due to acid generation, which in turn degrades the calcium phosphate layers, causing NO to be released from its donor, NONOates [213]. Silica‐based nanoformulations have been explored in the past decade for continuous and controlled release of NO. Hu et al. reported SNP‐loaded mesoporous silica NPs (SNP@MSN) for targeted delivery [214]. SNP@MSN generates higher levels of exogenous NO and produces a sustained IOP‐lowering effect for up to 48 h, using only 1/40 of the SNP dose. To avoid cytotoxicity and enable sustained NO release, a recent study developed a system using silica NPs surface‐modified with branched polyethylene imine (BPEI), which in turn stabilizes NONOates via molecular interactions with nearby primary amine groups (Figure 5A) [215]. The total amount of NO released and the maximum NO flux from BPEI‐NO‐NPs were 3.5 µmol mg^−1^ and 2266 ppb mg^−1^, respectively, indicating favorable human cell viability (Figure 5B,C). BPEI‐NO‐NPs showed initial spontaneous degradation of NONOates, releasing a burst of NO at 9 min, but demonstrated a delayed release compared to other NO‐releasing Si‐NP systems, consistent with their bactericidal activity (Figure 5D,E). Further investigation of BPEI‐NO‐NPs in a mouse keratitis model showed significantly enhanced corneal wound healing compared to controls, highlighting their multifunctional nanotherapeutic potential for keratitis treatment (Figure 5F). Although these nanoformulations ensure NO release in a controlled and extended manner, they do not provide deeper tissue penetration. Hence, hollow mesoporous organosilica nanocapsules (HOS), which can efficiently co‐deliver hydrophobic JS‐K (J_R_) and hydrophilic _L_‐Arg (L_O_), were developed, resulting in HOS‐J_R_L_O_ (Figure 5G) [216]. Once in the trabecular meshwork (TM)/Schlemm's canal (SC) area, HOS‐J_R_L_O_ can be reduced and oxidized by ascorbic acid, subsequently catalyzing eNOS to generate NO, thereby lowering IOP. HOS‐J_R_L_O_ released significantly more NO into the TM/SC area than its counterparts (Figure 5H). In a mouse IOP model, Cav1 knockout activates eNOS, leading to increased IOP. HOS‐J_R_L_O_ treatment in these mice reduced IOP via increasing NO levels in the TM/SC region, as evidenced by the NO fluorescent signal observed (Figure 5I). All these findings confirm the efficient delivery of HOS‐J_R_L_O_ in the TM/SC microenvironment for enabling better tissue penetration and NO delivery in a non‐invasive manner, ideal for precision glaucoma therapy. Although conventional nanoporous systems are introduced that respond to changes in pH, temperature, and light, their non‐targeted delivery can cause cytotoxicity by the formation of peroxynitrite. To address this issue, magnetic mode of delivery strategies has opened new avenues in I/R treatment. Navati et al. introduced a biocompatible gadolinium oxide‐based paramagnetic NPs coated with RSNOs (SNO‐PMNPs) [217]. In a hamster I/R injury model, applying a magnetic field to SNO‐PMNPs promoted localized NO release during early reperfusion, improving therapeutic outcomes. Beyond magnetic systems, chitosan‐based composites have been studied as effective NO delivery platforms. A novel copper‐chitosan composite successfully delivered NO to human corneal and limbal epithelial injuries, accelerating wound healing [218]. Similarly, for microbial keratitis, a contact lens gel composed of poly‐_L_‐lysine functionalized with NONOate moieties maintained bactericidal NO concentrations at physiological pH for up to 15 h [219]. In conclusion, these advanced NO‐delivery platforms represent a paradigm shift in ocular pharmacotherapy, moving beyond conventional eye drops toward precision‐guided multifunctional strategies. With continued innovation in materials science and targeted delivery systems, NO‐based therapeutics are poised to drive next‐generation treatments for glaucoma, corneal injuries, retinal disorders, and ocular infections.

### 3.3 Nerve Regeneration

NO is crucial for the functioning of the nervous system. In the central nervous system (CNS), NO release is vital for cognitive function, the induction and maintenance of synaptic plasticity involved in sleep, body temperature regulation, hunger, neural secretion, and reproductive processes [220]. In the peripheral nervous system (PNS), NO modulates non‐adrenergic, non‐cholinergic relaxation of smooth muscle cells and acts as a neurotransmitter in the gastrointestinal and urogenital tracts [221, 222]. NO exhibits both neuroprotective and neurotoxic properties, depending on its concentration, source, and duration of exposure [220]. At physiological levels, NO generated via nNOS and eNOS enhances cytoskeletal reorganization, axon growth, synaptic plasticity, remyelination, and angiogenesis, all of which are foundational for neural repair [220, 223]. In contrast, sustained high levels of NO from iNOS, often triggered by inflammatory responses, can lead to oxidative and nitrosative neurotoxicity, impairing nerve regeneration [220]. Studies on brain I/R suggest that eNOS is upregulated during early stages, producing small amounts of NO that promote vasodilation and protect brain vasculature [224, 225]. However, a lack of blood and oxygen supply triggers a calcium signaling cascade to activate nNOS, resulting in high NO levels [226]. Furthermore, prolonged iNOS activity during reperfusion results in excessive NO accumulation in the brain [227]. Excessive NO is neurotoxic, contributing to neural cell death, secondary inflammation, and damage to the blood‐brain barrier, resulting in I/R injury in the brain [228, 229]. Studies indicate that the hyperfunction of nNOS and iNOS causes more NO release, leading to mitochondrial failure, axonal degeneration, and glial scarring in traumatic brain and spinal cord injury [230, 231].

Numerous studies have demonstrated NO's involvement in the occurrence of neurodegenerative disorders such as Alzheimer's disease (AD), Parkinson's disease (PD), and amyotrophic lateral sclerosis [232]. In AD, decreased endothelial NO results in increased amyloid β (Aβ) expression and regulation of amyloid precursor protein in the brain vasculature [233]. Expression of nNOS and iNOS is significantly implicated in neurodegeneration. The interplay between nNOS and iNOS in the expression of NO is crucial to the progression of neurodegenerative diseases. For instance, a study reported that ischemia reduced nNOS in hippocampal neurogenesis by upregulating iNOS [234]. Inhibition of iNOS in AD transgenic mice reduced Aβ‐induced neurotoxicity, suggesting that NO generated by iNOS induces nitrosative stress that drives AD pathogenesis [232]. Excess NO/ONOO^−^ damage oligodendrocytes and myelin, leading to demyelination, impaired axonal conduction, and contributing to neurodegenerative diseases such as multiple sclerosis [235]. Hence, its neuroprotective and neurotoxic abilities underscore the importance of tight spatiotemporal regulation of NO signaling following injury/disease.

**FIGURE 6:**
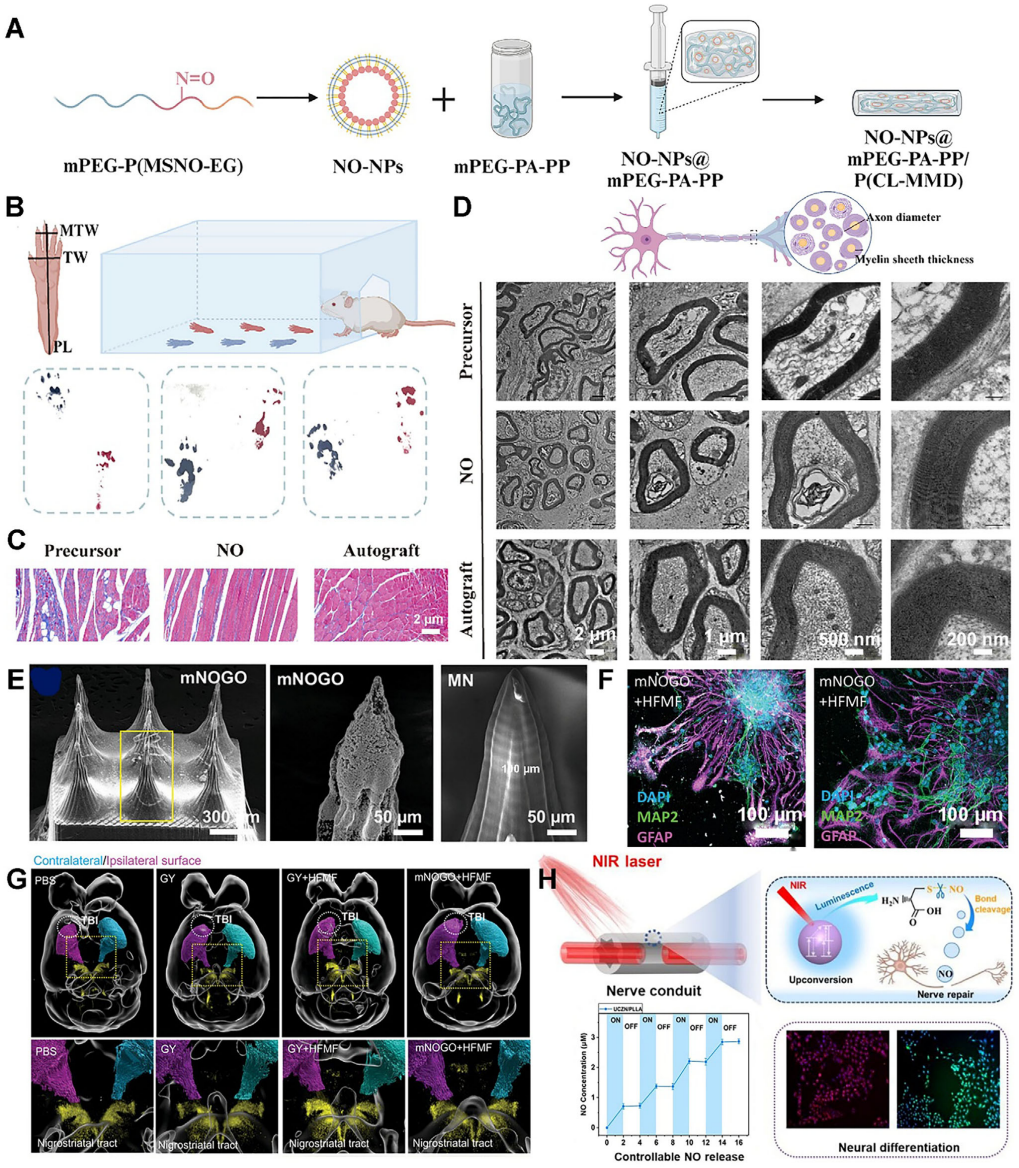
NO application in nerve regeneration. (A) Diagram depicting the steps for the fabrication of NO‐releasing nerve conduits. (B) Footprint images of rat gait analysis at the 12th week after sciatic nerve injury, with the injured side depicted in red and the uninjured side in blue. (C) Representative images of H&E staining of the cross‐sectional gastrocnemius muscle. (D) TEM images of regenerated axons in different groups at magnifications of 2 µm, 1 µm, 500 nm, and 200 nm. Reproduced with permission.[238] Copyright 2024, Elsevier. (E) SEM images of high‐frequency magnetic field (HFMF)‐responsive NO‐release gold yarn‐dynamos (mNOGO) at different magnifications. (F) Immunofluorescence images of neural differentiation of NSC sphere in the presence of mNOGO+HFMF. (G) Reconstructed 3D images of the whole brains of mice with TBI after treatment with various nanomaterials, with dopamine neurotransmission and tyrosine hydroxylase (TH) displayed in color. The injured side (right brain) is colored purple, and the normal side (left side) is colored blue. Adapted under Creative Commoms Liscence CC‐BY.[240] Copyright 2023, John Wiley and Sons. (H) Schematic diagrams of the fabrication of the nerve scaffold and NIR‐controlled NO release for nerve repair. Reproduced with permission.[241] Copyright 2025, Elsevier.

NO's therapeutic potential lies in precisely tuned delivery systems that exploit its regenerative properties while avoiding neurotoxicity. Bridging the gap between pathology and therapy, the emerging paradigm positions NO donors and their delivery platforms as precision tools to recalibrate NO bioactivity, thereby converting a mediator of neural injury into a driver of neuroprotection and functional recovery. NO donors such as SNP, GSNO, NONOates, and sydnonimines have been extensively studied for neuroprotection [236]. However, these NO donors are neurotoxic in vitro due to the generation of byproducts in excess of NO, leading to adverse effects in the neural microenvironment [237]. The multifactorial complexity of neurodegenerative disorders, combined with the narrow window of NO, off‐target effects, and the diverse mechanisms of NO release from different donors, continues to make their treatment challenging. NO‐delivery systems offer a means to control and regulate NO release. Huo et al. developed an nerve conduit composed of a rolled electrospun poly(ε‐caprolactone‐*co*‐*3S*‐(methyl)‐morpholine‐2,5‐dione) (P(CL‐MMD)) fiber tube filled with an injectable, temperature‐sensitive hydrogel ‐ (methoxy poly(ethylene glycol)‐polyalanine‐polyphenylalanine (mPEG‐PA‐PP)) ‐ embedded with macromolecular NO donor NPs ((poly(ethylene glycol methyl ether)‐poly(nitrosylation mercaptosuccinic acid‐*co*‐ethylene glycol) (mPEG‐P(MSNO‐EG)) [238]. Efficacy of the NO conduit was tested in a rat peripheral nerve injury (PNI) model. Rat footprints were collected to calculate sciatic nerve function index values (Figure 6B), indicating a significant difference among the three treatment groups. Animals treated with NO nerve conduits showed enhanced motor function recovery and axonal regeneration when compared to the precursor group (Figure 6C,D). Another study demonstrated the delivery of NO‐releasing silica NPs blended with fibrin gel to peripheral nerves in rats following acute crush injury to the sciatic nerve [239]. NO delivered to the damaged sciatic nerve led to revascularization and the growth of myelinated axons.

Recent advances in nanomedicine have opened new avenues for the development of NP‐loaded NO donors that allow for stimulus‐responsive release triggered by pH, light, or enzymatic activity, aligning NO delivery with pathological microenvironments. One such novel approach is the development of an electromagnetic messenger system in traumatic brain injury (TBI) that employs high‐frequency magnetic fields to trigger NO release in the brain [240]. The electromagnetic messenger system NO‐release gold yarn‐dynamos, poly(S‐nitrosoglutathione) (pGSNO), and embedded in an implantable silk microneedle (Figure 6E). Upon implantation and exposure to a magnetic field, the gold yarn‐dynamos stimulated NO release from PGSNO, reducing glial scar formation by regulating microglia infiltration and astrocyte activation during early stages of TBI (Figure 6F). Sustained release of NO induces angiogenesis and neurogenesis, promoting functional recovery of neurons in vivo (Figure 6G). A recent work constructed a nerve conduit by utilizing NIR‐triggered upconversion NP core with a ZIF‐8 shell loaded with S‐nitrosocysteine (CysNO) and blended with poly‐_L_‐lactic acid (PLLA) powder (UCZN) [241]. Exposure of UCZN to NIR triggered the release of NO from CysNO in a controlled manner in vitro, thereby achieving deep penetration and promoting nerve repair (Figure 6H). NO‐delivery platforms hold significant promise for advancing nerve regeneration; however, overcoming challenges in dose control, targeting, stability, and clinical translation is essential. The next generation of intelligent, multimodal, and patient‐tailored systems may transform NO from a double‐edged mediator of injury into a precision therapeutic for nerve repair.

### 3.4 Immunomodulation

Immunomodulation is a process of normalizing or restoring the immune system to its optimal state by means of either strengthening or suppressing overstimulated immune reactions. The immune system can be modulated for prevention as well as therapy. Both vaccination and immunotherapy rely on the body's immune machinery to recognize, target, and eliminate harmful agents (pathogens or cancer cells) [242]. Instead of directly killing pathogens or tumors with drugs, both approaches prime or modulate immune responses for long‐term protection or disease control [242, 243]. On the contrary, immunosuppressants are utilized to control an overstimulated immune system observed in autoimmune disorders and inflammation [244]. NO functions as both an effector molecule against pathogens and a regulator of immune responses. Its impact is highly dependent on concentration and context‐specific, ranging from antimicrobial cytotoxicity to suppression of excessive inflammation and immune tolerance [245]. As immunotherapy in the context of pathogens and cancer has been extensively discussed in our previous review, this work will provide insight into the role of NO in immunomodulation in autoimmune disease and inflammation, as well as the recent advancements in the field of NO donors and delivery platforms [11].

NO has pleiotropic roles in immunity, acting as both a pro‐inflammatory effector and an anti‐inflammatory regulator depending on its concentration, timing, and cellular source [246]. During inflammation, high levels of NO expressed by activated M1 macrophages due to the action of iNOS can kill pathogens while damaging the host tissues [247]. It can also promote oxidative amplification by producing peroxynitrite, driving protein nitration, lipid oxidation, and DNA damage, all of which are involved in inflammation [247, 248]. As a proinflammatory influencer, NO can enhance cytokine production (e.g., tumor necrosis factor‐α (TNF‐α), interlukin‐1β (IL‐1β), interleukin‐6 (IL‐6)), increase vascular permeability, causing edema and leukocyte recruitment at the site of inflammation [249, 250]. NO functions as an anti‐inflammatory agent by inducing T‐cell apoptosis, improving vasodilation, perfusion, and clearance of inflammatory remnants, along with M2 polarization, to promote tissue repair [251, 252]. In autoimmune diseases, it contributes to both pathogenesis and resolution. Its paradoxical nature makes NO a central immunomodulator, with therapeutic implications in chronic inflammatory and autoimmune disorders. Excess NO contributes to synovial inflammation, joint destruction, and pain sensitization in rheumatoid arthritis (RA) [253, 254]. Overproduction of NO and peroxynitrite damages oligodendrocytes and myelin, exacerbating neuroinflammation, causing multiple sclerosis [255]. iNOS‐derived NO in pancreatic islets promotes β‐cell apoptosis, accelerating autoimmune destruction, and promoting type 1 diabetes [256]. Systemic lupus erythematosus is induced by NO‐driven nitrosative stress that can modify self‐antigens, increasing their immunogenicity and amplifying autoantibody production [253]. On the other side, NO has regulatory roles in autoimmune diseases. NO can suppress antigen presentation by dendritic cells, restraining autoreactive T cell responses [257]. Controlled NO delivery has been shown to reduce disease severity in models of RA and inflammatory bowel disease by restoring immune balance [258, 259].

**FIGURE 7:**
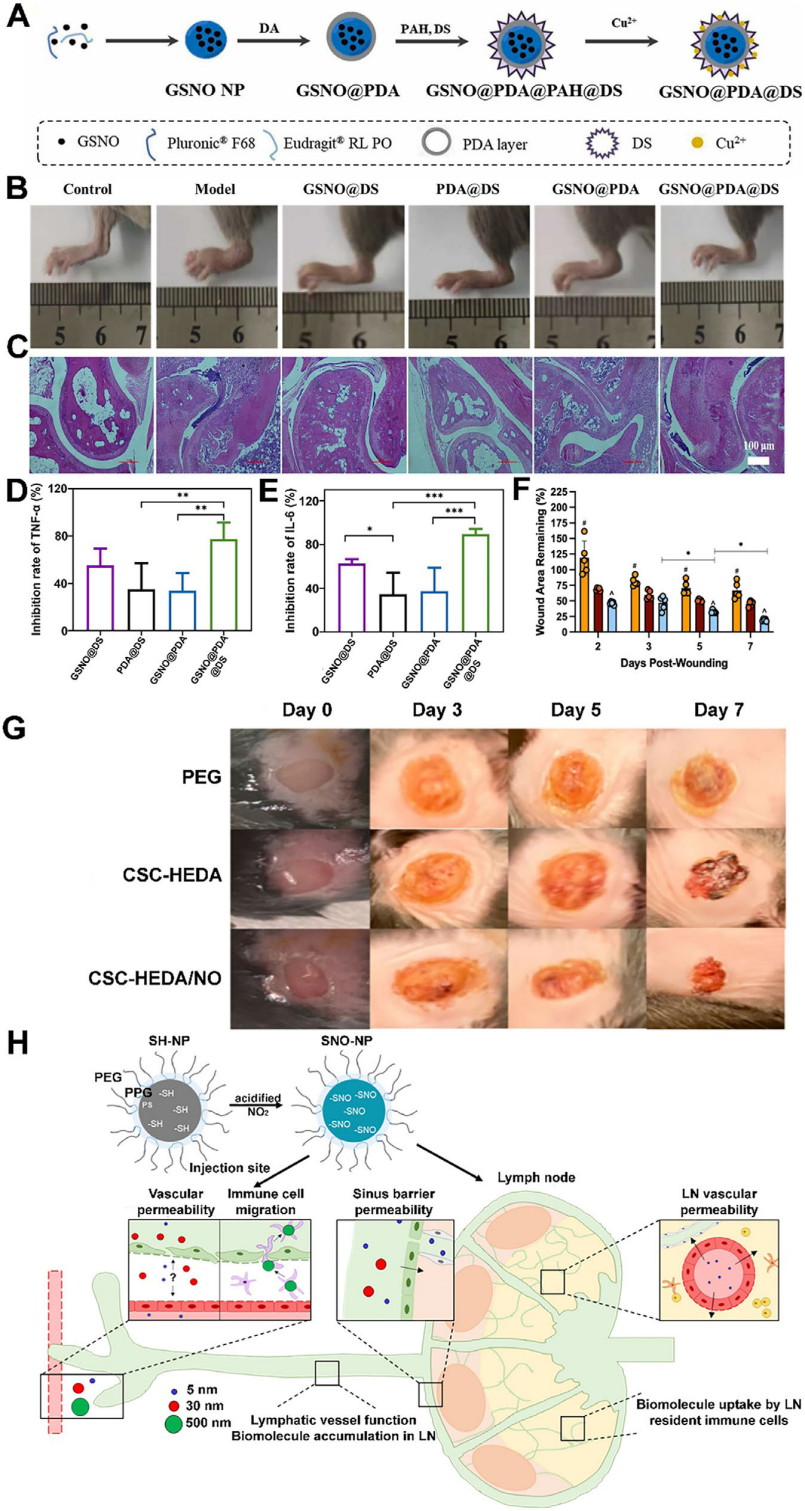
NO role in immunomodulation. (A) Illustration of the synthesis of GSNO@PDA@DS NPs. (B) Images of mice paws after treatment with saline, GSNO@DS, PDA@DS, GSNO@PDA, and GSNO@PDA@DS. (C) H & E staining of the ankle joints. (D, E) Levels of proinflammatory cytokines (D) IL‐6 and (E) TNF‐α in different groups. Adapted under the Creative Commons License.[264] Copyright 2024, Elsevier. (F) Percentage of initial wound area remaining following daily treatment with PEG 400 (orange), CSC‐HEDA (maroon), or CSC‐HEDA/NO (blue)  (G) Representative images of wounds on different days after treatment with PEG 400 (control), 50 mg kg^−1^ CSC‐HEDA in PEG 400, or 50 mg kg^−1^ CSC‐HEDA/NO in PEG 400. Reproduced with permission.[265] Copyright 2024, American Chemical Society. (H)  Schematic illustrating SNO‐NP production and the downstream effects of NO following peripheral administration. Reproduced with permission.[267] Copyright 2021, Elsevier.

NO sits at the crossroads of inflammation and autoimmunity, capable of both exacerbating tissue damage and restraining aberrant immune responses. Its dual nature highlights the importance of precision‐controlled delivery rather than systemic modulation. NO donors and advanced delivery platforms have emerged as promising therapeutic strategies for modulating inflammation and autoimmune diseases [260]. The development of nanoformulations allows spatiotemporal control of NO release, aiming to harness its immunoregulatory properties while minimizing adverse effects [245, 260]. Despite remarkable progress, the application of nanotechnology in NO delivery for immunomodulation with respect to inflammation and autoimmune diseases remains in its early stages of development. Studies utilized scavengers to combat NO overproduction during inflammation in RA [261, 262]. Nanohydrogel with NO‐scavenger (NO‐Scv gel) was prepared by polymerization of acrylamide and NO‐cleavable cross‐linker (NOCCL), which cleaves by consuming NO molecules, thereby reducing inflammation in vitro and suppressing the onset of RA in vivo [262]. However, NO scavenging alone often fails to achieve balanced immunomodulation because of its bidirectional effects. Combining NO scavenging with other critical targets in immunomodulation can provide a more balanced and sustainable effect. In line with this idea, Geng et al. developed a block polymer PEG_10_‐*b*‐PNAPA_30_‐*b*‐PEG_10_  comprising NO‐scavenging N‐(2‐aminophenyl) acrylamide hydrochloride (NAPA) and a cysteine‐triggered H_2_S donor (a trithiocarbonate chain transfer agent) [263]. The synergistic polymer can decrease NO levels and release H_2_S, which can further inhibit NO and pro‐inflammatory cytokine levels via the NF‐kβ pathway, thereby reducing inflammation in vitro. It showed promising therapeutic potential in a rat model of collagen‐induced arthritis. Although it is widely accepted that RA exhibits higher NO levels involved in escalating the progression of the disease. Contrarily, Luo et al. designed a dual‐function nanodrug formulation that couples NO release with ROS scavenging to provide a more balanced and sustainable immunomodulatory effect than NO donors alone, making them especially valuable in inflammation and autoimmune disease therapy [264]. The authors developed an NP system with encapsulated GSNO and surface‐charged with polydopamine (PDA). The GSNO@PDA was simultaneously coated with negatively charged polyallylamine hydrochloride (PAH), followed by positively charged dextran sulfate (DS), followed by surface anchoring of Cu^2+^, yielding GSNO@PDA@DS particles (Figure 7A). GSNO@PDA@DS particles prevented macrophage polarization into the M1 phenotype in vitro. In the collagen‐induced arthritis model, intravenous injection of these particles reduced the paw swelling in RA mice compared to the control (Figure 7B). Further, the degree of inflammation, characterized by the infiltration of inflammatory cells, was decreased histologically, along with a reduction in the levels of pro‐inflammatory cytokines, IL‐6, and TNF‐α, in GSNO@PDA@DS‐treated mice (Figure 7C–E).

In contrast to autoimmune diseases, chronic wounds have reduced NO bioavailability owing to endothelial dysfunction, oxidative stress, or impaired NOS functions. To treat chronic wounds, an amine‐modified chondroitin sulfate (CSC) conjugated with a NO donor, NONOate (CSC‐HEDA/NO), was generated [265]. Although this complex did not show a significant change in fibroblast migration compared to CSC and CSC‐HEDA in cell studies, it promoted maximum wound closure in mice (Figure 7F,G). In recent years, targeted drug delivery has gained considerable interest in immunotherapy. However, nanoscale drug delivery systems have limited access to parenchymal immune cells, thereby limiting their efficiency. Lymph nodes (LN) play a crucial role in the immune response, and to reach the lymphatics, the delivery systems must overcome biological barriers that inhibit drug accumulation, limiting access to LN‐localized immune cells and achieving therapeutic efficacy [266]. NO is a potent regulator of lymphatic transport via lymphatic vessel contractility and permeability, making it a great tool in nanocarrier delivery to LNs. However, its potential is limited in LN due to high reactivity and low molecular weight [267]. Hence, Sestito et al. developed S‐nitrosated NPs (SNO‐NP) that can deliver NO to LN [267]. They tested the effect of NO released by SNO‐NP on the lymphatic transport and distribution of co‐delivered molecules in LN. While the SNO‐NP application did not interfere with the large molecules’ transport, passive lymph drainage, and accumulation of small and medium‐sized molecules in LN, it significantly increased the delivery of molecules about the size of 30 nm into the LN and their uptake by immune cells (Figure 7H).

Since NO functions as a double‐edged mediator of disease, both therapeutic delivery and accurate NO detection are critical. The field of NO detection is still evolving. Accurate detection of NO is crucial to precisely measure NO to guide therapeutic strategies and improve patient outcomes in diseases such as RA, chronic wounds, cardiovascular disorders, and cancer. In this context, a group developed a mitochondrial‐targeted NIR ratiometric fluorescent probe to monitor NO levels in early stages of RA [268]. In vitro and in vivo studies showed that the probe detected NO with high sensitivity and selectivity and may have great potential for application in the diagnosis of early RA.

**FIGURE 8:**
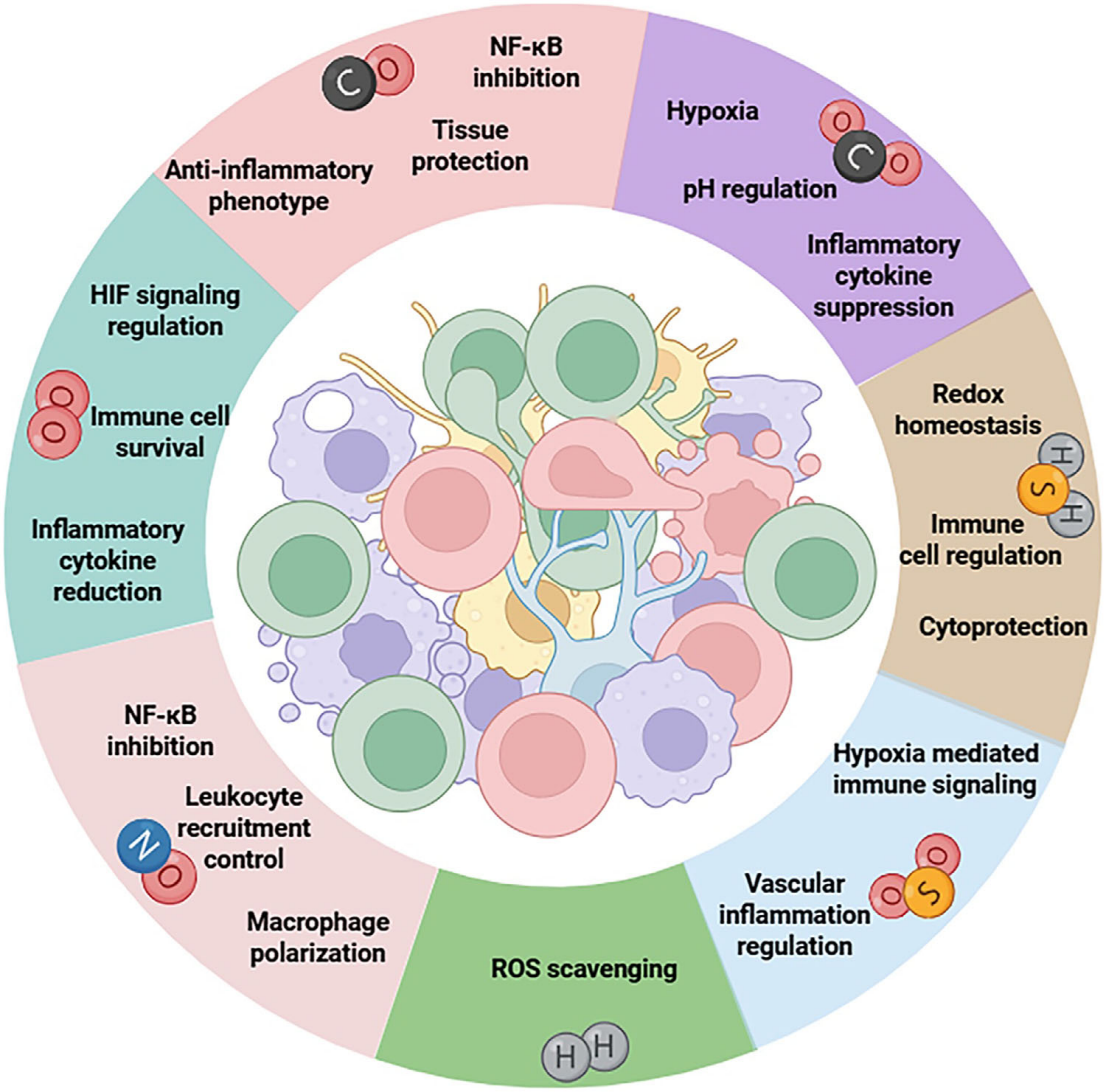
Gas‐mediated immunomodulatory network. Schematic depicting nitric oxide (NO) as a central, concentration‐dependent immune regulator with cell‐specific effects. Crosstalk between NO and other gaseous mediators (CO, H_2_S, H_2_, CO_2_, SO_2_, and Xe) integrates redox and immune signaling to control inflammation, autoimmunity, and tissue repair, highlighting the need for precise, material‐enabled delivery strategies. Created in BioRender.com.

Figure 8 presents a schematic overview of gasotransmitter‐mediated immunomodulation, with NO consolidated as a single central element. NO acts as a concentration‐ and context‐dependent immune regulator: low, tightly controlled levels support immune homeostasis, endothelial function, and inflammation resolution, whereas elevated or sustained NO amplifies antimicrobial activity and inflammatory signaling but may promote tissue damage if unchecked [240]. Immune cells interpret NO signals in a cell‐specific manner, shaping macrophage polarization, leukocyte recruitment, T‐cell activation, and cytokine balance [11, 245, 246, 247, 248, 249].

The therapeutic and diagnostic landscape for NO is rapidly evolving, driven by the dual recognition that NO is both a critical regulator of physiology and a pathological mediator when dysregulated. The future of NO‐based biomedical strategies lies in achieving precision delivery of NO at the right place, at the right time, and at the right dose, while simultaneously developing technologies capable of real‐time monitoring of NO dynamics in tissues. Futuristic approaches may explore real‐time biosensing, molecular imaging, multifaceted theranostics, and precision medicine to revolutionize treatments for inflammation, autoimmune diseases, neurodegeneration, cardiovascular disorders, and chronic wounds.

### 3.5 Diagnostics

NO plays a pivotal role in diagnostics due to its involvement in numerous physiological and pathological processes, including vasodilation, immune response, neurotransmission, and inflammation. Abnormal NO levels are linked to diseases such as cardiovascular disorders, neurodegenerative diseases, cancer, and infections. Therefore, monitoring NO can provide valuable diagnostic insights and enable early detection of disease states. Precise, in situ quantification of NO is critical for unraveling its spatiotemporally complex biology and for guiding targeted therapies. Novel strategies in the design and fabrication of NO sensors/probes are focused on achieving real‐time, highly sensitive, and selective detection directly within complex biological environments, moving far beyond traditional lab‐based assays [269, 270, 271]. Key innovations leverage advanced nanomaterials, sophisticated fluorescent/optical probes (NIR, ratiometric, two‐photon, and photoacoustic (PA) probes), chemi‐resistive gas sensors, electrochemical nano‐sensors, and flexible electronics to enable in vivo and wearable sensing [269, 270, 271, 272, 273, 274, 275, 276, 277, 278, 279, 280, 281, 282, 283, 284, 285].

**FIGURE 9:**
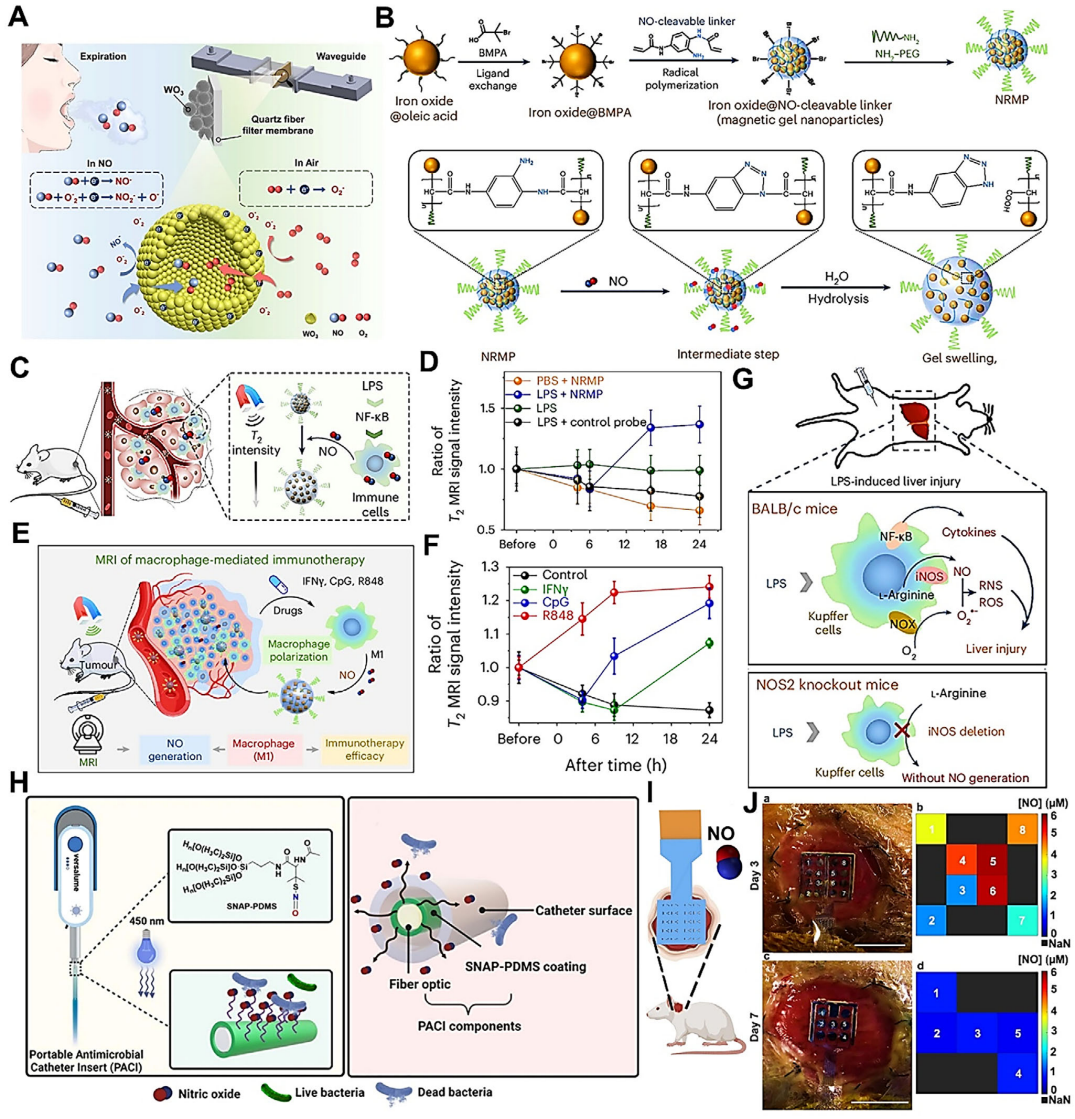
NO probes and wearable sensors for diagnostics. (A) NO‐sensing mechanism of the waveguide microwave gas sensor (MGS). Reproduced with permission.[291] Copyright 2024, American Chemical Society. (B) Schematic illustration for the synthetic procedure of the NRMP and the reaction of the NRMP with NO. (C) Schematic illustration showing MRI of LPS‐induced NO generation in the tumor. (D) The ratio of the *T*_2_ MRI signal intensity for a tumor from each group, at different time points post‐injection of NRMP. (E) Schematic illustration of employing MRI to image NO produced by M1 macrophages and to monitor macrophage‐mediated immunotherapy. (F) The ratio of *T*_2_ MRI signal intensity for the tumor from each group. (G) Schematic illustration showing the mechanism for LPS‐induced generation of NO in BALB/c mice, and no generation of NO in NOS2^−/−^ mice (inducible NO synthase gene knockout). Reproduced with permission [294]. Copyright 2024, Springer Nature. (H) Development of a battery‐powered Portable Antimicrobial Catheter Insert (PACI) and application scenario of the PACI. Reproduced with permission [299]. Copyright 2024, Elsevier. (I) Schematic of multiplexed, electrochemical, real‐time, localized, inflammation‐tracking NO sensor (MERLIN) array. (J) Representative day 3 MERLIN NO measurement on rat skin wound in vivo (a). Scale bar = 1 cm. NO concentration mapping readout (b). Representative day 7 MERLIN NO measurement on rat skin wound in vivo (c). Scale bar = 1 cm. NO concentration mapping readout (d). Adapted under the Creative Commons Attribution.[300] Copyright 2025, Science AAAS.

Biosensors based on electrochemical sensors, especially amperometric NO sensors, allow real‐time detection of NO in biological fluids [281]. For instance, NO‐releasing amperometric probes have been applied in vivo to monitor NO levels in arteries, providing insights into vascular function and the efficacy of therapeutic interventions. These sensors often employ NO‐permeable membranes or catalytic coatings to enhance selectivity and stability [270, 286, 287]. One of the most successful and widely adopted diagnostic uses of NO is the measurement of fractional exhaled NO (FeNO). This is a simple, non‐invasive breath test that provides a direct indicator of a specific type of airway inflammation [288, 289, 290]. Zhang et al. developed a highly sensitive, portable Ni, Co‐MOF‐74‐carbon nanotube‐poly(acrylonitrile)‐based sensor for detecting airway inflammation by measuring NO in breath [289]. The resulting sensor demonstrated excellent performance for detecting NO at room temperature, with a detection range of 30–1000 ppb (parts per billion) and a limit of detection of approximately 18.6 ppb. The performance of the sensor was comparable to that of standardized medical devices when validated using breath samples from patients with airway inflammation [289]. In another recent work using highly sensitive waveguide circuits, Wang et al. designed a microwave gas sensor integrated with hollow multishelled structured WO_3_ (HoMSs‐WO3) for NO detection in the screening of COVID‐19 patients [291]. The WO_3_ sensor operates by changes in resistance upon NO adsorption, which modulates the waveguide signal, allowing real‐time, sensitive detection (Figure 9A). The developed device demonstrated linear response for NO concentrations from 10 to 100 ppb, with a high sensitivity of 39.27 dB/ppm at room temperature, aligning with the targeted levels of 15–100 ppb as observed in COVID‐19 patients.

Fluorescent NO probes have been developed for imaging at both cellular and tissue levels. Among these, ratiometric probes allow quantitative detection with high spatial resolution, enabling visualization of NO in living tissues [274, 278, 292]. For example, recent probes have achieved micron‐resolution imaging of NO distribution throughout the whole brain of mice with PD using 3D PA imaging, capturing depth‐specific information from 0 to 8 mm [278]. Lucero developed APNO‐1080, the first NIR‐II acoustogenic probe, designed to detect endogenous, cancer‐derived NO in deep tissue [292]. Created via a two‐phase tuning approach, this probe offers the potential for noninvasive detection of tumors in hard‐to‐reach areas of the body. Similarly, a recent study introduced a “turn‐on” PA probe for in vivo detection of NO in encephalitis [293]. The probe reacts with NO, shifting to a planar ground state that generates a new NIR absorption band suited for deep‐tissue imaging. Upon excitation, it reverts to a twisted state, amplifying the PA signal. This dual “turn‐on/turn‐off” mechanism provides high selectivity, sensitivity, and quantitative detection, allowing noninvasive imaging and severity assessment of encephalitis [293].

Another interesting study demonstrated the design and fabrication of a promising noninvasive NO‐responsive magnetic probe (NRMP) to determine the role of NO in physiological and pathological processes [294]. The NRMP probe was composed of PEG‐coated superparamagnetic iron oxide NPs and an NO‐sensitive cleavable linker to allow crosslinking and surface modification. When the probe encounters NO, the sensitive linker is cleaved and breaks cross‐links, causing the NPs to disperse and separate, which subsequently changes magnetic properties (Figure 9B). This change in magnetic properties led to a significant increase in the T2 relaxation time, which is Vis as a strong “turn‐on” signal on a magnetic resonance imaging (MRI) scan. The efficacy of NRMP has been validated across various biomedical applications. For instance, in vivo MRI revealed enhanced contrast of the NO level increased in the tumor microenvironment of a 4T1‐tumour‐bearing mouse model induced by injecting lipopolysaccharide (LPS) followed by NRMP treatment (Figure 9C). The quantified data also confirmed the NO dose‐dependent effect on MRI signaling in comparison to the control groups (Figure 9D). Similarly, to image NO via MRI, the NRMP‐treated groups demonstrated brighter signals and distinct dynamics in the T2 MRI images in screening immunotherapeutic drugs and LPS‐induced liver (Figure 9E–G) [294].

Medical device‐associated infections remain a major global healthcare challenge, frequently resulting in serious complications, prolonged hospital stays, and increased healthcare costs. Wearable inserts that can release NO offer a promising way to prevent device‐associated infections. Commercial catheters were impregnated with SNAP or coated with GSNO via electrochemical generation, liquid infusion, or dip coating to control NO release and showed broad‐spectrum antibiofilm activity [287, 295, 296, 297]. The smartphone‐compatible NO‐releasing Disposable Catheter Disinfection Insert uses a similar light‐activation approach with a smartphone app to control NO release and showed reduced microbial attachment by > 99% and eradicated ∼97% of pre‐colonized bacteria on catheters [298]. Later, the same group demonstrated that the Portable Antimicrobial Catheter Insert (PACI) connects to a wearable light module and can be activated with a single click, providing controlled NO release for at least 24 h (Figure 9H) [299]. Research has shown that NO released by the PACI significantly reduces bacterial viability (> 90%) against common strains like *S. aureus, Staphylococcus epidermidis* (*S. epidermidis*), and *Proteus mirabilis* (*P. mirabilis*) without harming host cells. This adaptable design makes it suitable for various medical devices, including catheters [299].

Accurate and timely assessment of wound healing is crucial, especially for chronic wounds where complications can arise. Traditionally, wound evaluation relies heavily on visual assessment, which is subjective and may not always reflect the underlying biochemical changes. MERLIN (Multiplexed, electrochemical sensor array designed for in vivo, real‐time, and localized measurement of NO concentrations) comprises multiple detection points on a flexible array, allowing for the mapping of NO gradients across a wound (Figure 9I) [300]. This provides a detailed picture of inflammation distribution, potentially revealing localized hot spots or variations in healing progress. The sensor's innovative design incorporates a bilayer of selective materials, including electrochemically polymerized poly‐5‐amino‐1‐naphthol (poly‐5A1N) and a spray‐coated fluorinated xerogel, which work together to ensure high sensitivity to NO while minimizing interference from other electrochemical species, such as nitrites, ascorbic acid, and uric acid, commonly found in wound environments. In fact, authors demonstrated that MERLIN showed a high sensitivity of 883 ± 283 nA µm ^−1^ cm^2^ and impressive selectivity against nitrites (∼27 900‐fold), ascorbic acid (∼3800‐fold), and uric acid (∼6900‐fold). It offers continuous monitoring of NO levels, providing real‐time insights into the inflammatory state of the wound. In vivo testing on rat skin wounds demonstrated MERLIN's ability to accurately track NO levels over seven days (Figure 9J). Overall, wearable NO‐releasing inserts offer a promising approach to prevent device‐associated infections, potentially improving patient outcomes and reducing healthcare burdens. Continued research and development in this area are crucial to realizing the full potential of this technology.

## 4 Other Gas Therapies

In recent decades, the field of gas biology has expanded beyond NO, uncovering a diverse array of endogenous and exogenous gases that serve critical regulatory roles in mammalian physiology. Once dismissed as toxic or inert, many small gaseous molecules are now recognized as *gasotransmitters* or therapeutic gases capable of modulating signaling pathways, cellular redox balance, and immune responses. Among these, CO, H_2_S, H_2_, O_2_, CO_2_, SO_2_, and Xe have emerged as important candidates for clinical translation due to their potent cytoprotective, anti‐inflammatory, and tissue‐regenerative properties. This review focuses on recent advances in biomaterial‐ and nanotechnology‐based delivery platforms that achieve spatially and temporally controlled gas release, effectively mitigating issues of volatility, toxicity, and rapid diffusion.

### 4.1 Carbon Monoxide

CO, historically notorious for its toxicity and association with combustion‐related poisoning, has undergone a remarkable reappraisal over the past few decades. Once recognized solely as an environmental pollutant and a potent inhibitor of oxygen transport, CO is now acknowledged as an endogenous signaling molecule with wide‐ranging physiological and therapeutic roles. Endogenously, CO is produced during the oxidative degradation of heme by the action of enzyme heme oxygenase (HO), a stress‐inducible enzyme that catalyzes the conversion of heme into biliverdin and free iron, thus releasing CO [301, 302]. Two main isoforms of heme oxygenase exist: HO‐1, which is inducible under oxidative, inflammatory, and hypoxic conditions, and HO‐2, which is constitutively expressed in several tissues, including the brain and vasculature [303, 304]. The biological effects of CO largely mirror the cytoprotective functions of HO‐1, establishing CO as a key effector molecule in cellular defense mechanisms [303, 304].

Under physiological conditions, CO is generated at low concentrations and functions as a gasotransmitter modulating cellular homeostasis [305, 306]. The basal levels of CO in tissues and exhaled air reflect HO activity and can increase significantly during cellular stress, inflammation, or hypoxia [307]. CO primarily interacts with heme‐containing proteins, such as hemoglobin, myoglobin, cytochrome c oxidase, and soluble guanylate cyclase (sGC), thereby influencing a wide range of biological processes [308, 309]. Unlike its toxic effects at high concentrations, low endogenous levels of CO act as a fine‐tuned signaling mediator, contributing to vasoregulation, neurotransmission, mitochondrial function, and redox balance [310]. CO exerts its biological effects primarily through the activation of sGC, stimulation of mitogen‐activated protein kinase (MAPK) pathways, and suppression of pro‐inflammatory transcription factors such as NF‐κB [311, 312]. Therapeutically, CO exhibits potent anti‐inflammatory, anti‐apoptotic, and cytoprotective effects [310]. Despite its therapeutic promise, the clinical use of CO gas remains challenging due to its high affinity for hemoglobin and the risk of systemic hypoxia at elevated concentrations [313]. To circumvent these limitations, researchers have developed advanced delivery systems, including CO‐releasing molecule (CORM)‐loaded NPs, polymeric carriers, and photoresponsive CO donors, that enable localized and temporally controlled CO release [314, 315]. Preclinical studies demonstrate that low‐dose CO exposure or administration of CORMs can mitigate I/R injury, prevent transplant rejection, and reduce pulmonary fibrosis [314].

**FIGURE 10:**
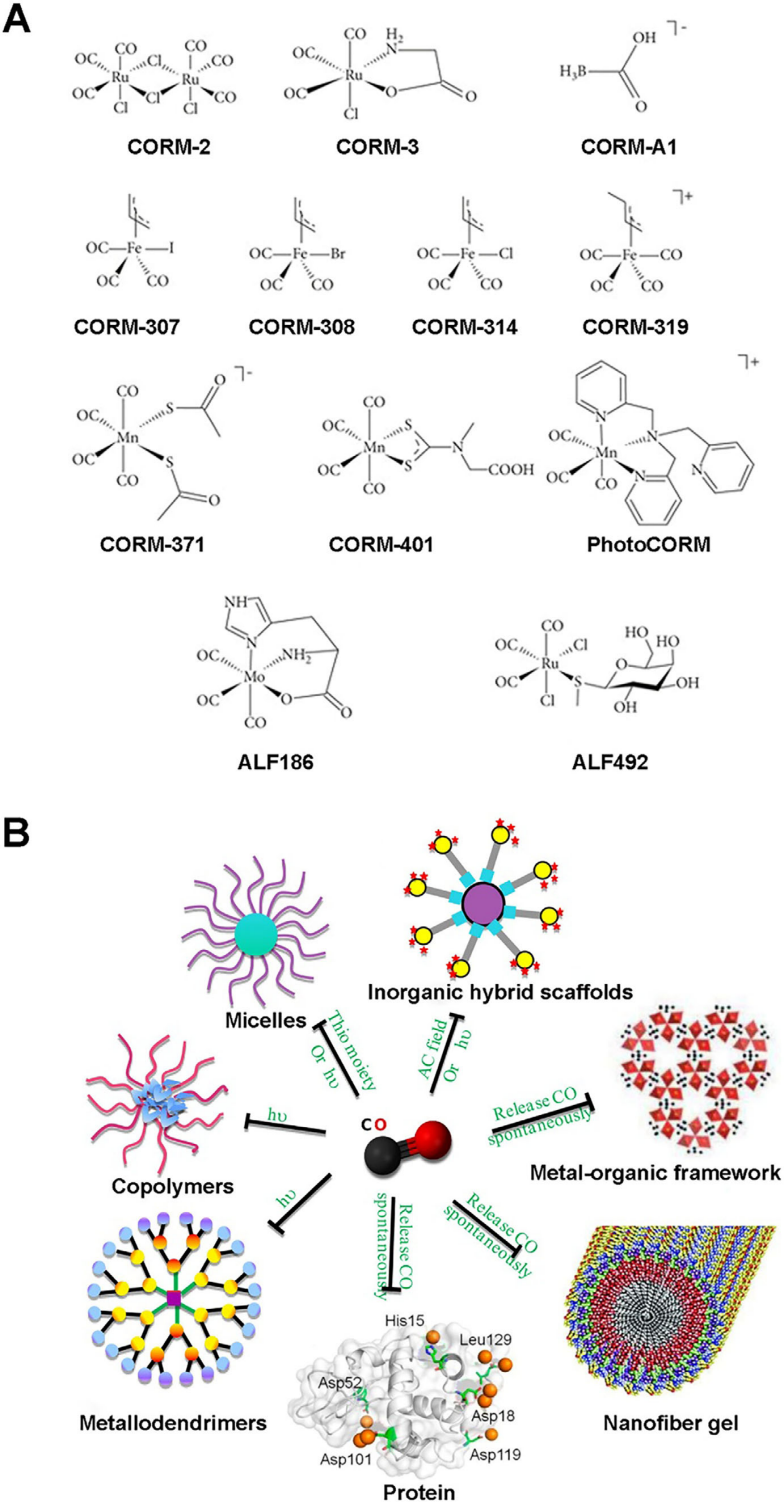
CO donor and delivery systems. (A) Overview of CO‐releasing molecules (CORM) classes. Adapted under Creative Commons Attribution.[316] Copyright 2018, John Wiley and Sons. (B) Nanocarrier encapsulation strategies: CO donors embedded in liposomes, micelles, and polymer matrices for sustained and localized release. Reproduced with permission.[318] Copyright 2015, American Chemical Society.

CORMs function as *prodrugs* that liberate CO upon activation by specific physicochemical stimuli, such as aqueous dissolution, light, temperature, enzymatic action, or changes in redox potential or pH [314, 315]. Beyond serving as CO donors, many CORMs also exhibit unique pharmacological profiles related to their structural composition, influencing their biological compatibility, tissue penetration, and therapeutic potential. CORMs are broadly categorized based on their metal center, ligand environment, and the mechanism by which they trigger CO release [314]. CORMs include metal‐based CORMs, photo‐CORMs, and enzyme‐activated CORMs (Figure 10A) [316]. Nanocarrier encapsulation strategies for CORMs involve embedding CO donors in liposomes, micelles, and polymer matrices for sustained and localized release. CORMs can be covalently conjugated to biomaterials such as peptides, proteins, polymers, dendrimers, and vitamins or to metal and inorganic nanomaterials such as gold and iron oxide NPs [315, 317]. Copolymers functionalized with CORM‐2 display better water solubility and a slow and sustained CO release from the polymer‐based CORM (Figure 10B) [314, 318]. The key physicochemical characteristics and biological activities of reported CORMs are summarized in Table 2.

**TABLE 2:** Key physicochemical characteristics and biological activities of reported CORMs.

CORM	Chemical Composition / Core Structure	Trigger / Activation Mechanism	CO Release Profile	Key Biological Effects	Refs.
CORM‐1	[Mn_2_(CO)_10_]	Thermally activated (spontaneous under physiological conditions)	Rapid CO release; half‐life <10 min in buffer	Anti‐inflammatory, vasodilatory	[419]
CORM‐2	[RuCl_2_(CO)_3_]_2_	Hydrolysis‐triggered CO liberation in aqueous media	Fast release (<10 min)	Cytoprotection, inhibition of macrophage activation, NF‐κB suppression	[419, 420]
CORM‐3	[Ru(CO)_3_Cl(glycinate)]	Water‐soluble; spontaneous CO release in physiological buffer	Controlled release (t½ ≈ 20–30 min)	Anti‐inflammatory, anti‐apoptotic, antimalarial	[421, 422, 423, 424]
CORM‐A1	Sodium boranocarbonate (Na_2_H_3_BCO_2_) – metal‐free	Hydrolytic activation under physiological pH	Slow, sustained release (t½ ≈ 21 min–1 h)	Metal‐free donor, antioxidant, anti‐inflammatory, renoprotective, neurogenesis	[425, 426, 427]
CORM‐401	[Mn_2_(CO)_10_]‐based; solid‐phase complex	Thermally and redox‐activated	Sustained release over hours	Anti‐oxidative, vasoprotection	[428, 429, 430]
CORM‐L1	[Mn(CO)_3_(tris(pyrazolyl) methane)]	Light triggered	Wavelength dependent	Tumor targeting	[431]
Photo‐CORMs	Ru, Mn, or Re carbonyl complexes with photo‐labile ligands (e.g., bipyridine, phenanthroline)	Light‐triggered (UV–vis or NIR)	On‐demand, rapid release upon irradiation	Spatiotemporal CO control, tissue‐targeted therapy	[432]
Enzyme‐responsive CORMs	Peptide‐, ester‐, or phosphonate‐functionalized CO donors	Enzymatic activation (esterases, phosphatases)	Controlled, tissue‐specific release	Targeted CO delivery to inflamed or ischemic tissues	[433]
Polymer‐anchored CORMs	CORMs covalently or physically bound to polymeric scaffolds or NPs	Hydrolytic or photochemical	Sustained, localized release	Localized cytoprotection, enhanced biocompatibility	[434]
Hybrid Gas Donors (CO + NO/H_2_S)	Dual gas‐releasing platforms combining CORMs with other gasotransmitter donors	Redox or enzymatic	Sequential or simultaneous release	Synergistic anti‐inflammatory, angiogenic, and cytoprotective effects	[435]

**FIGURE 11:**
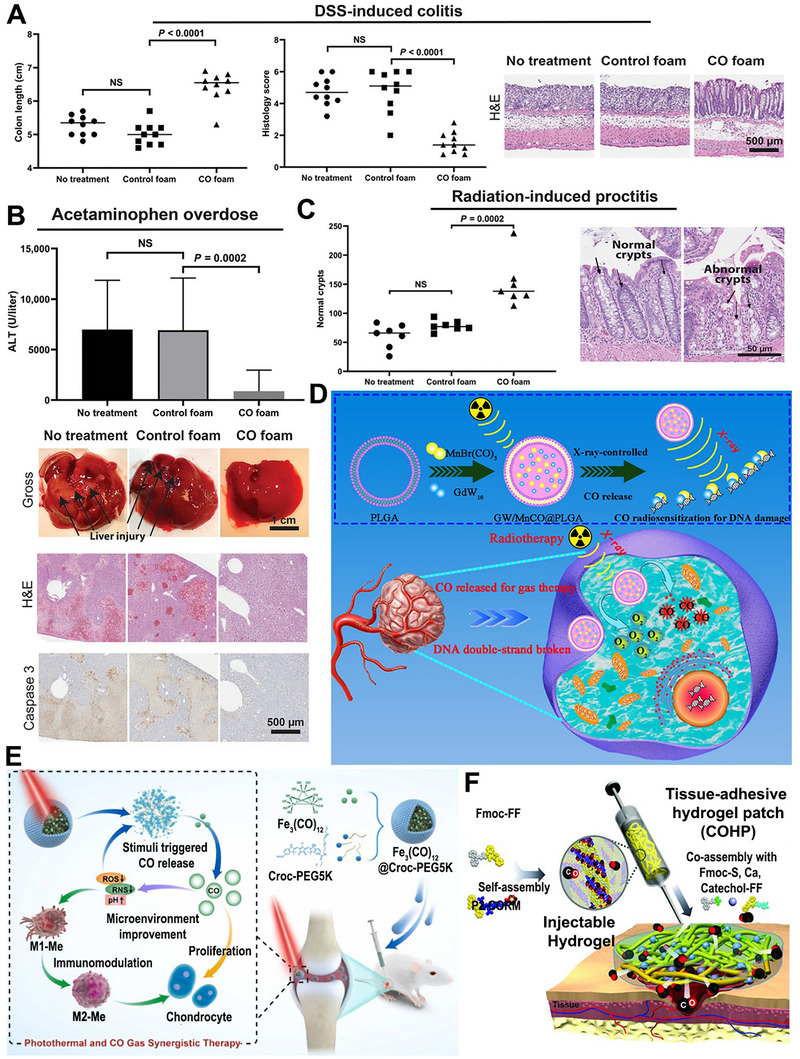
CO‐based therapies and their biomedical targets. (A–C) The therapeutic application of CO‐GEMs (5 g/kg) across three preclinical rat models. (A) DSS‐induced colitis model, Colon length, histology scores, and H&E staining of liver tissue of DSS‐treated animals are shown. (B) The acute liver failure model, where ALT was assessed 24 h after acetaminophen overdose, and the respective histopathology of liver tissue showing H&E staining and activated Caspase‐3 of treated groups. (C) In a radiation‐induced proctitis model, rats were treated with CO‐GEMs rectally 1 day prior (5 g/kg), within 1 h before irradiation (5 g/kg), and once daily for 8 days (5 g/kg) after exposure to 18 Gy of radiation directed to the rectum. H&E staining of rectal tissue depicts the quantity of normal intestinal crypts in animals treated with CO‐GEMs. Reproduced with permission [320]. Copyright 2022, Science AAAS. (D) Diagrammatic illustration of the synthesis of X‐ray‐triggered CO‐releasing micelles and their antitumor action. Reproduced with permission [329]. Copyright 2022, American Chemical Society. (E) Conceptual diagram of Fe_3_(CO)_12_@Croc‐PEG_5K_ for NIR fluorescence imaging‐guided photothermal and CO gas synergistic therapy of OA. Reproduced with permission[330] Copyright 2023, Elsevier. (F)  Schematic of the production of injectable supramolecular CO‐releasing hydrogel, COH, and the bioadhesive hydrogel patch, COHP. Reproduced with permission[331]. Copyright 2018, John Wiley and Sons.

CORMs are being investigated for various applications, including cancer therapy, cardiovascular therapy, and inflammation modulation. Administration of CO‐releasing molecule 2 (CO‐RM2) before and after transplantation resulted in ∼60% of mice surviving, compared with 0% survival in mice treated with inactive CORM [319]. Byrne et al. utilized preclinical disease models to demonstrate the efficacy of CO gas‐entrapping materials (CO‐GEMs) (Figure 11A–C) [320]. In a dextran sulfate sodium (DSS)‐induced colitis model, CO‐GEMs significantly increased colon length (P < 0.0001) and improved histological scores compared to air‐GEM and untreated groups (Figure 11A). CO‐GEMs (5 g/kg) promoted therapeutic effects in an acute liver failure model induced by acetaminophen (APAP) (250 mg/kg, i.p.), by significantly reducing alanine aminotransferase (ALT) levels (P < 0.0002) and liver injury compared to control foam (Figure 11B). In a radiation‐induced proctitis model, CO‐GEMs preserved intestinal crypts (P < 0.0002) compared to controls (Figure 11C) [320]. Another study reported that mice treated with static magnetic articulation (SMA)/CORM2 exhibited significantly less tissue damage and a histological appearance similar to that of normal mice in a DSS‐induced colitis model, indicating suppression of inflammatory cytokines such as TNF‐α (P < 0.001) and IL‐6 (P < 0.001) [321].

Further, nanocarrier‐based formulations have been developed to enhance stability, prolong circulation, and enable targeted, stimuli‐responsive CO delivery. A detailed review of the CO‐releasing nanomaterials (CORNMs) was summarized by Ning et al. [315]. CORNMs represent a novel and versatile platform for therapeutic gas delivery. By combining nanotechnology with CO's biological activity, researchers can target disease tissues, control dosing, and reduce systemic toxicity [315]. Current applications span cancer therapy, cardiovascular protection, neuroprotection, wound healing, and anti‐inflammatory treatments [317]. In cancer treatment, CO can suppress tumor progression by inducing apoptosis, regulating mitochondrial function, and modulating the hypoxic tumor microenvironment. Its ability to reduce oxidative stress and inflammation makes it an adjunct or sensitizer to chemotherapy and radiotherapy [322]. Most hybrid nanomaterial‐based CORNMs have been developed using MOFs, crystalline porous structures composed of metal ions or clusters coordinated to organic ligands, which offer high tunability, large surface area, and controlled gas‐release properties [323]. Zhong et al. developed a ZIF‐8‐based nanocarrier (ZCM) co‐loaded with camptothecin and manganese carbonyl, enabling combined chemotherapy and CO gas therapy [324]. In acidic tumor environments, ZCM released both agents, where camptothecin induced H_2_O_2_ and MnCO generated CO in situ, enhancing oxidative stress and tumor killing. The system showed strong antitumor efficacy and low toxicity in CT26 tumor‐bearing mice. Wang et al. designed an H_2_O_2_‐responsive CORNM (MnCO@Ti‐MOF) by incorporating MnBr(CO)_5_ into a Ti‐based MOF [323]. The system released CO through H_2_O_2_‐triggered reactions in tumor environments, inducing selective apoptosis in high‐H_2_O_2_ cancer cells while sparing healthy cells, highlighting its potential for targeted cancer therapy. Cai et al. developed a cascaded nanocatalyst (RG‐Mn@H) by loading MnCO and glucose oxidase (GOD) into RGD‐modified hollow mesoporous organosilica NPs for targeted cancer therapy [325]. The mesoporous inorganic NP system selectively targets αvβ3‐positive tumor cells, where acidic tumor conditions trigger GOD dissociation, MnCO release, and CO generation, leading to enhanced ROS production, tumor starvation, and apoptosis both in vitro and in vivo. Another study developed a multifunctional CO delivery system for MRI‐guided photothermal and CO synergistic cancer therapy using MnCO@CuS NPs [326]. The CuS core enabled NIR‐induced heating (up to 45°C) for photothermal ablation, while MnCO released CO in response to H_2_O_2_ and laser stimulation within the tumor microenvironment. This dual action promoted apoptosis, enhanced ROS generation, and provided MRI contrast via Mn^2+^ release, demonstrating superior in vitro and in vivo anticancer efficacy compared to either therapy alone. Yeh et al. developed mesoporous Prussian blue (m‐PB)‐CO/PEG NPs, a NIR‐responsive CO delivery system [327]. Upon 808 nm irradiation, the NPs released CO and generated heat, enabling synergistic photothermal and CO therapy, which induced significant tumor apoptosis and suppression in vitro and in vivo. A separate work reported PEG‐modified, bicarbonate‐functionalized defective WO_3_ nanosheets (P@DW/BC) that respond to NIR light [328]. Under 808 nm irradiation, they enable photothermal tumor ablation and in situ CO generation via CO_2_ conversion, achieving synergistic photothermal therapy (PTT) and CO therapy with enhanced tumor suppression. Besides NPs, micelles have also attracted considerable attention as platforms for CO delivery. Liu et al. developed gadolinium tungsten/manganese tricarbonyl @poly(lactic‐co‐glycolic acid) (GW/MnCO@PLGA) micelles, which release CO upon X‐ray irradiation (Figure 11D) [329]. Activation of the GW NPs radiosensitizer generates superoxide anions that trigger CO release from MnCO, leading to synergistic CO and ROS‐induced DNA damage, apoptosis, and enhanced cancer cell killing, demonstrating potential for combined radiation and gas therapy.

Other than anti‐tumor applications, CO delivery platforms are developed for tissue regeneration and treating diseases such as OA. Wang et al. developed triiron dodecacarbonyl@croconaine‐derived dye, (Fe_3_(CO)_12_@Croc)‐PEG_5_K, a NIR‐responsive CO delivery system for OA (Figure 10E) [330]. Upon 808 nm irradiation, it released CO to reduce inflammation, regulate pH, and enable photothermal therapy, effectively suppressing inflammatory factors in cells and preserving cartilage integrity in OA rat models. In another instance, Kim et al. developed CO‐releasing peptide hydrogels (COH) and adhesive patches (COHP) (Figure 10F) [331]. These systems allow controlled CO release to suppress LPS‐induced inflammation, protect cardiomyocytes, and offer robust mechanical properties and tissue adhesion. COH is injectable, while COHP can be applied as a patch, representing an innovative approach for gasotransmitter‐based regenerative medicine. Several NP‐ and micelle‐based CO delivery systems have been developed, including mesoporous silica NPs, polymeric micelles, and hydrogel‐encapsulated CO donors. These platforms enable controlled site‐specific CO release, which modulates macrophage polarization, suppresses pro‐inflammatory cytokines (e.g., TNF‐α, IL‐6), and reduces oxidative stress, providing targeted anti‐inflammatory and immunomodulatory effects with minimal systemic toxicity [332, 333, 334]. CO‐releasing nanomaterials represent a new frontier in gasotransmitter‐based therapeutics. Their ability to deliver CO in a controlled, localized, and biocompatible manner has demonstrated preclinical success across cancer, regenerative, and inflammatory disorders. Future efforts should focus on developing clinically translatable CORNMs with predictable pharmacokinetics, safety validation, and smart responsiveness to disease‐specific stimuli. Beyond its established anti‐inflammatory and anti‐tumor properties, recent studies demonstrate that CO exhibits significant antibacterial activity, particularly when delivered via CORMs. CORMs induce rapid death of pathogenic bacteria such as *E. coli* and *S. aureus*, with increased efficacy under the hypoxic conditions typical of infection sites [335]. The integration of CORMs into biomaterial platforms represents a promising antibacterial approach by facilitating localized, sustained, and stimuli‐responsive CO delivery, thereby addressing the limitations associated with free CO gas. For instance, methionine‐based block copolymers synthesized via RAFT polymerization and conjugated to ruthenium‐based CORMs exhibit enhanced aqueous solubility, prolonged CO release, and effective inhibition of *P. aeruginosa* biofilm formation [336]. Additionally, multifunctional polymer coatings incorporating ultrasonic‐responsive CORM units have been engineered for medical devices, enabling on‐demand CO release that eradicates bacteria, suppresses inflammation, and maintains biosafety [337]. These biomaterial‐based CO delivery systems provide several advantages over conventional antibiotics, including controlled release kinetics, improved stability, site‐specific targeting, and a reduced risk of antimicrobial resistance. Consequently, CO‐loaded biomaterials are emerging as a promising alternative for the prevention and treatment of bacterial infections, particularly in implant‐associated and biofilm‐mediated contexts. The transformation of CO from a recognized toxin to a sophisticated gasotransmitter underscores its significant therapeutic potential for treating inflammatory, malignant, and infectious diseases. The use of advanced nanocarriers and stimuli‐responsive CORMs enables precise and localized delivery, facilitating the exploitation of CO's cytoprotective and antibacterial properties while maintaining systemic safety.

### 4.2 Hydrogen Sulfide

H_2_S, long considered a toxic gas due to its characteristic odor and ability to inhibit cellular respiration at high concentrations, has emerged over the past two decades as an important endogenous gasotransmitter alongside NO and CO, with broad regulatory roles in physiology and pathophysiology. Endogenously, H_2_S is produced through enzymatic pathways primarily involving cystathionine β‐synthase (CBS), cystathionine γ‐lyase (CSE), and 3‐mercaptopyruvate sulfur transferase (3‐MST), each with tissue‐specific distribution and function [338, 339]. CBS is predominantly expressed in the brain and contributes to neuroprotective signaling, CSE is largely found in the cardiovascular system, regulating vascular tone and blood pressure, and 3‐MST, located in mitochondria, participates in cellular redox balance and energy homeostasis [338, 340]. Non‐enzymatic H_2_S production also occurs under certain conditions through the reduction of sulfur‐containing compounds, providing an additional layer of endogenous regulation [339]. Mechanistically, H_2_S exerts its effects via multiple interconnected pathways: it induces vasodilation primarily through activation of adenosine triphosphate (ATP)‐sensitive potassium channels in vascular smooth muscle, contributing to blood pressure regulation and cardioprotection [339]. It demonstrates anti‐inflammatory effects by inhibiting NF‐κB signaling, suppressing pro‐inflammatory cytokines such as TNF‐α, IL‐1β, and IL‐6, and modulating macrophage polarization toward an anti‐inflammatory M2 phenotype [341]. It also possesses potent antioxidant and cytoprotective properties, enhancing endogenous glutathione and thioredoxin systems, activating Nrf2‐mediated transcription of antioxidant enzymes, and maintaining mitochondrial function under oxidative stress [342, 343]. In the nervous system, H_2_S modulates synaptic transmission, promotes long‐term potentiation, and protects neurons from oxidative damage, thereby mitigating neurodegenerative processes observed in models of AD and PD, as well as ischemic stroke [343, 344, 345]. Beyond these effects, H_2_S promotes angiogenesis and tissue repair by stimulating vascular endothelial growth factor (VEGF) expression and endothelial cell migration, and it participates in metabolic regulation by influencing glucose and lipid metabolism in the liver, muscle, and adipose tissue [346, 347].

These diverse biological roles have prompted extensive exploration of H_2_S in therapeutic contexts. In cardiovascular disease models, H_2_S reduces vascular inflammation, limits atherosclerotic progression, and protects myocardial tissue during I/R injury [339, 346, 347]. In neurodegenerative disorders, it alleviates oxidative stress, preserves neuronal viability, and improves cognitive outcomes [343]. H_2_S suppresses systemic and localized inflammation, as evidenced in sepsis, colitis, and arthritis models in inflammatory conditions [348]. In regenerative medicine and wound healing, H_2_S facilitates angiogenesis, fibroblast proliferation, collagen deposition, and repair of chronic or diabetic wounds [349].

**FIGURE 12:**
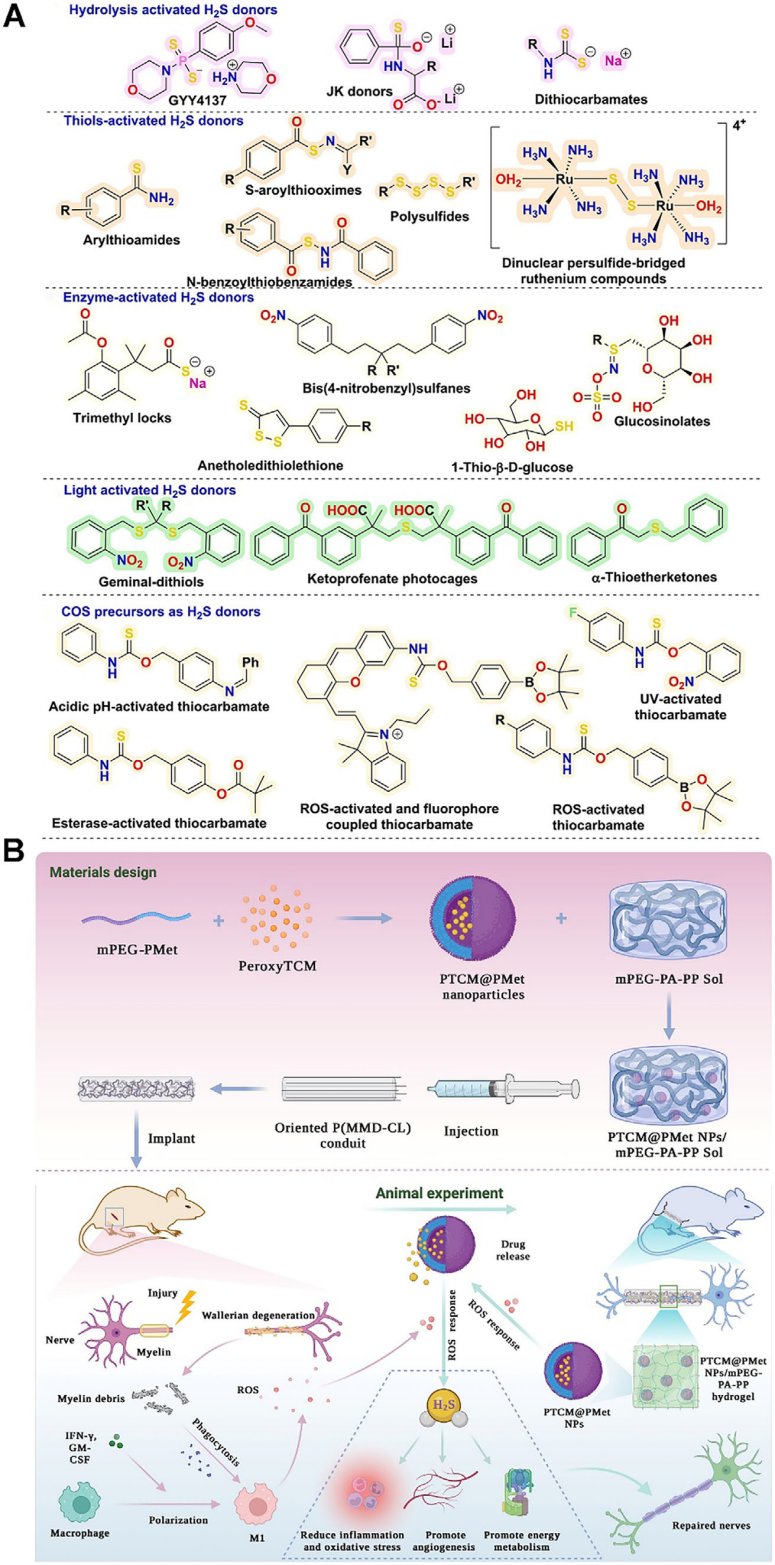
Strategies and applications of H_2_S‐releasing therapeutics. (A) Types of donors and their mechanisms for releasing H_2_S. Adapted under the Creative Commons License [351]. Copyright 2025, Elsevier. (B) Schematic illustration of constructing an intelligently responsive multi‐effect messenger biomimetic nerve scaffold for PNI repair. Adapted under the Creative Commons Attribution CC BY‐NC[355]. Copyright 2023, Science AAAS.

Despite its potential, direct administration of H_2_S gas is impractical due to volatility, rapid systemic clearance, and toxicity, necessitating the development of controlled delivery systems [350]. Various small‐molecule and polymeric H_2_S donors have been developed, with release often triggered by environmental cues such as pH, redox potential, or enzymatic activity (Figure 12A) [351]. Examples include sulfide salts (e.g., NaHS, Na_2_S), garlic‐derived sulfur compounds (Lawesson's reagent, GYY4137), phosphorodithioates, 1,2‐dithiole‐3‐thiones (DTTs), N‐(acylthio)‐benzamides, S‐SH compounds, dithioperooxyanhydrides, arylthioamides, gem‐dithiol compounds, ketoprofenate‐caged compounds, and thioamino acids [352]. The integration of H_2_S donors such as N‐(benzoylthio)benzamide (NSHD‐1) into polymeric fibers offers a promising approach for controlled, localized delivery of H_2_S [350]. The fibers maintain structural uniformity while enabling sustained, stimulus‐responsive H_2_S release in biological environments. This dual functionality is particularly valuable in cardiovascular and neural repair, where precise temporal and spatial H_2_S release can reduce oxidative stress, mitigate inflammation, and support tissue regeneration [350]. Ex vivo studies using isolated rabbit arteries and veins indicate that H_2_S‐releasing coatings can regulate coagulation, decrease thrombus formation, and improve blood flow, highlighting their therapeutic potential [350].

Nanotechnology‐based carriers have expanded the possibilities for H_2_S delivery, including polymeric NPs (e.g., PLGA or PEGylated systems), mesoporous silica NPs, liposomes, hydrogels, and hybrid nanomaterials that provide spatiotemporal control, tissue targeting, and stimuli‐responsive release [350]. Recent advances have focused on smart delivery platforms that respond to pH, ROS, enzymes, or light to selectively release H_2_S in pathological microenvironments such as inflamed tissues, tumors, or ischemic sites, thereby maximizing therapeutic efficacy while minimizing off‐target toxicity [353, 354]. Integration with nanomaterials also enables combinatorial therapies, where H_2_S release is coupled with chemotherapy, PTT, or immunotherapy to achieve synergistic effects, as well as imaging‐guided interventions using H_2_S‐responsive probes for real‐time monitoring of gas release and treatment outcomes [350]. In PNI models, a ROS‐responsive H_2_S delivery system was fabricated in combination with an electrospun P(MMD‐CL) fiber conduit for PNI repair [355]. An H_2_S donor (peroxythiocarbanate (peroxyTCM)) encapsulated in an ROS‐sensitive polymer (mPEG‐PMet) synthesized by ring‐opening polymerization of l‐methionine N‐carboxy anhydride (Met NCA) initiated by mPEG‐NH_2_ and loaded into a thermosensitive hydrogel (methoxy poly(ethylene glycol)‐polyalanine‐polyphenylalanine (mPEG‐PA‐PP)) enables on‐demand H_2_S release at the injury site. The released H_2_S reduces inflammation and oxidative stress, protects neurons, promotes angiogenesis, and restores mitochondrial function, collectively enhancing nerve regeneration and repair through a pleiotropic gasotransmitter strategy (Figure 12B) [355]. Another study prepared H_2_S‐releasing scaffolds by electrospinning PCL with the H_2_S donor NSHD‐1 [356]. These H_2_S‐fibers showed sustained release (2–4 h peak, up to 20× longer than donor alone), reduced ROS, and protected cells from oxidative damage, highlighting their potential as bioactive wound dressings.

**FIGURE 13:**
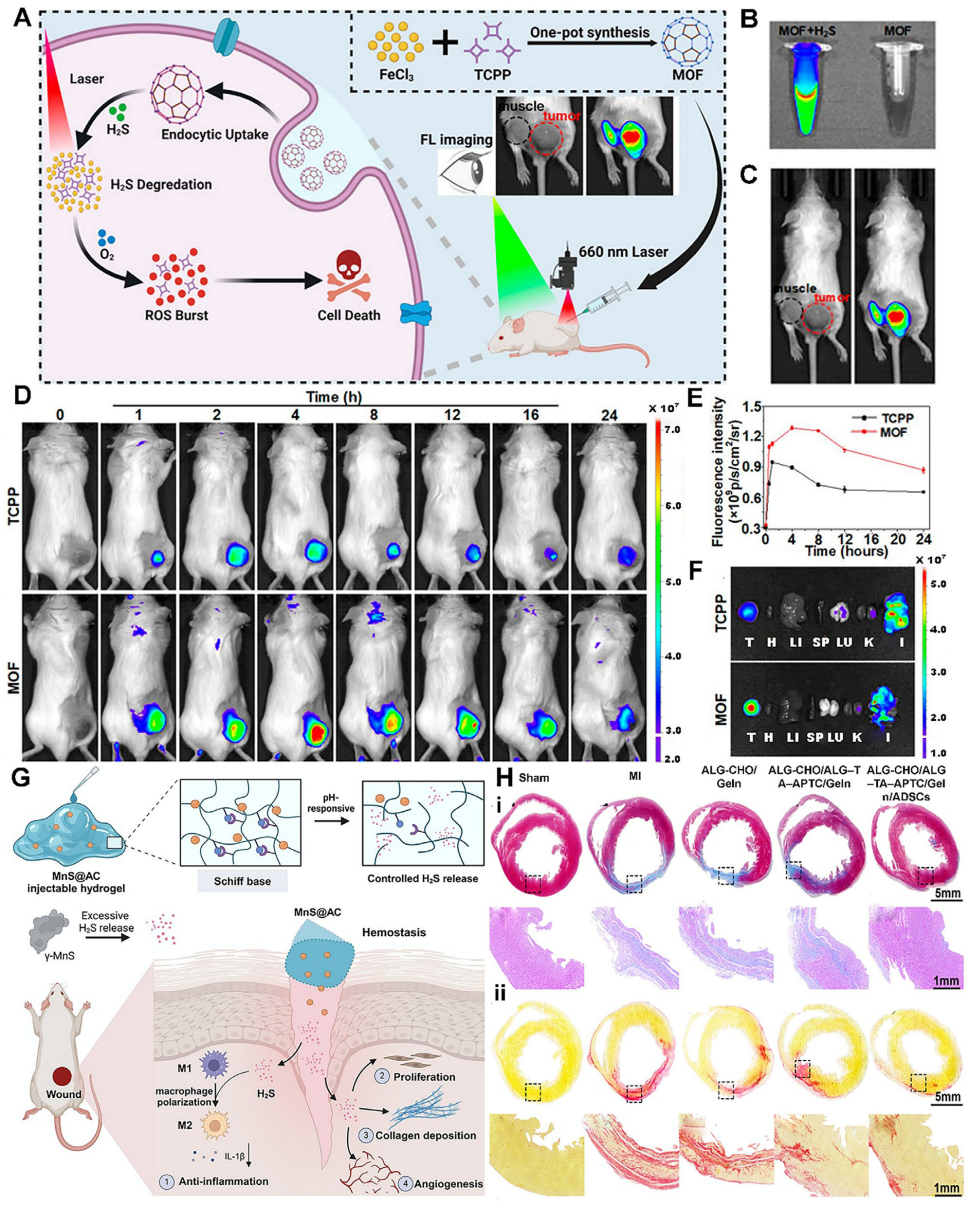
H_2_S‐based therapeutic applications. (A) Illustration of synthesis and mechanism of MOF‐mediated fluorescence imaging‐guided PDT. (B) Fluorescence imaging of MOF and MOF with donor‐NaHS. (C) Fluorescent imaging of the tumor and muscle after 30 min of intratumoral injection. (D) Fluorescence imaging of the tumor‐bearing mice after intravenous injection at different time intervals. (E) Fluorescent signal quantification of intravenous injections. (F) Fluorescent images of major organs and tumors 4 h post‐injection. (I: Intestine, H: Heart, LU: Lung, LI: Liver, K: Kidney, SP: Spleen, and T: Tumor). Adapted under the Creative Commons Attribution CC‐BY[357]. Copyright 2022, Froniters. (G) Illustration of preparation and mechanism of a pH‐responsive injectable hydrogel for wound treatment. (H) Histology images of cardiac structures after hydrogel injection: (i) Masson's trichrome staining for collagen and muscle; and (ii) Sirius red staining for collagen. Reproduced with permission[359]. Copyright 2015, American Heart Association, Inc. The bottom panels are the corresponding dashed square regions of the top panels.

As a diagnostic tool, nano‐photosensitizers activated by H_2_S have been prepared [357]. A H_2_S‐activatable MOF for targeted PDT of colorectal cancer (CRC) composed of meso‐tetra(4‐carboxyphenyl) porphine (TCPP) and Fe^3+^ was synthesized via a simple one‐pot method. In the H_2_S‐rich CRC microenvironment, the MOF degraded to release TCPP, switching its fluorescence and photosensitivity from “off” to “on” (Figure 13A). This enables selective imaging and PDT activation in tumor sites injected subcutaneously and intratumoral, offering a highly specific and low‐toxicity approach for CRC treatment (Figure 13B–D). Furthermore, the MOF showed stronger and longer‐lasting fluorescence than free TCPP, demonstrating enhanced stability and H_2_S‐triggered activation (Figure 13E). At 4 h post‐injection, ex vivo imaging showed brighter tumor fluorescence in the MOF group compared to free TCPP, indicating enhanced tumor targeting and selectivity (Figure 13F).

To regulate the uncontrolled and excessive release of H_2_S from its donors, a pH‐responsive injectable hydrogel (MnS@AC) was developed to deliver H_2_S in a controlled manner and promote wound healing (Figure 13G) [358]. Formed by Schiff base crosslinking of aldehyde hyaluronic acid and carboxymethyl chitosan, it released α‐phase MnS NPs under acidic conditions, ensuring stable, on‐demand H_2_S delivery. MnS@AC showed excellent biocompatibility and hemostasis, achieving 94.2% wound closure by day 13 and enhancing angiogenesis and collagen deposition while reducing inflammation without activating unwanted immune pathways. To treat myocardial infarction, a macromolecular H_2_S prodrug hydrogel was developed by grafting the H_2_S donor 2‐aminopyridine‐5‐thiocarboxamide onto oxidized alginate (ALG‐CHO) for sustained H_2_S release [359]. Incorporating tetraaniline and adipose‐derived stem cells (ADSCs) via Schiff base crosslinking with gelatin produced a conductive, adhesive hydrogel suitable for cardiac application. After myocardial injection, it showed enhanced ADSC retention, increased sulfide levels, and upregulated cardiac and angiogenic genes. The hydrogel treatment reduced inflammation, leading to improved heart function and smaller infarct size (Figure 13H). It decreased the fibrotic area and increased left ventricular wall thickness in the myocardial infarcted heart. This system offers promising therapy for myocardial infarction.

Preclinical studies have demonstrated that H_2_S‐releasing nanomaterials can protect cardiovascular tissues, reduce neuroinflammation, promote wound healing, and modulate immune responses, underscoring their translational potential. Nonetheless, challenges remain for clinical application, including precise dosing control, long‐term biocompatibility, biodegradability of nanocarriers, reproducibility of synthesis, and regulatory considerations. Future research directions include the development of personalized H_2_S delivery systems, combination therapies for chronic diseases, and multifunctional platforms that integrate therapeutic and diagnostic capabilities (theranostics). Taken together, the growing body of evidence underscores H_2_S as a versatile and potent gasotransmitter with therapeutic promise across cardiovascular, neurological, inflammatory, and regenerative medicine applications. Its successful integration into nanotechnology‐enabled delivery systems represents a paradigm shift, enabling targeted, controllable, and safe approaches to harness the biological functions of H_2_S, thereby advancing clinical translation and expanding the potential of gasotransmitter‐based therapeutics.

### 4.3 Oxygen

**FIGURE 14:**
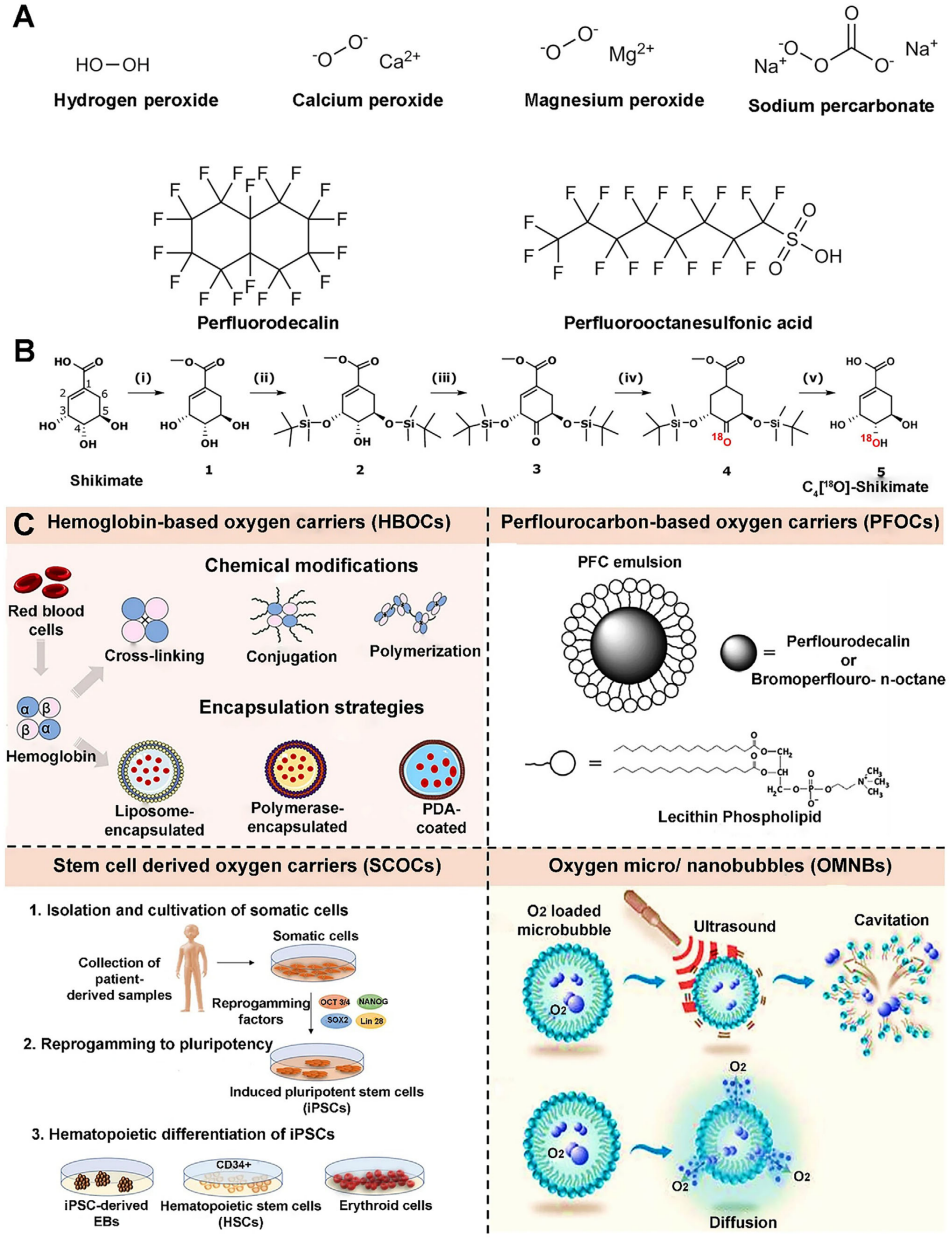
Diverse classes of O_2_‐releasing agents and synthetic carriers. (A) Prominent oxygen‐releasing compounds. Adapted under the Creative Common Attribution CC BY 4.0 [366]. Copyright 2024, Springer Nature. (B) Oxygen‐labeled shikimate derivatives: Structures of isotopically labeled (^18O) C_6_H_7_O_5_–shikimate compounds with various siloxane linkages (1–5). (C) Potential artificial oxygen carriers. Adapted under the Creative Commons CC BY‐NC‐ND [368] Copyright 2025, Springer Nature.

O_2_ is a vital molecule essential for cellular respiration, energy production, and overall tissue homeostasis. Beyond its classical role as a metabolic substrate, oxygen has increasingly been recognized as a key therapeutic agent, particularly in the context of hypoxia‐related pathologies, which include ischemic diseases, cancer, wound healing, and inflammatory disorders [360, 361, 362, 363]. Hypoxia, characterized by insufficient oxygen supply relative to tissue demand, can trigger maladaptive responses, such as angiogenesis, inflammation, metabolic reprogramming, and immune suppression, making localized oxygen delivery a promising strategy for disease management [364]. Traditionally, oxygen therapy has relied on inhalation methods, hyperbaric oxygen chambers, or systemic administration. However, these approaches are often limited by poor tissue penetration, rapid diffusion, systemic side effects, and the inability to target hypoxic microenvironments precisely [365]. Over the past decade, significant attention has focused on oxygen‐releasing agents and carriers capable of controlled, sustained, and localized oxygen release. These include peroxide‐ and percarbonate‐based compounds such as calcium peroxide (CaO_2_), magnesium peroxide (MgO_2_), hydrogen peroxide (H_2_O_2_), and sodium percarbonate, which release oxygen through hydrolytic or catalytic decomposition (Figure 14A) [366, 367]. Synthetic oxygen carriers such as perfluorocarbons (PFCs) like perfluorodecalin are highly effective due to their chemical inertness and high gas solubility [366]. Perfluorooctanesulfonic acid (PFOS) is another fluorinated surfactant known for its oxygen‐carrying capacity and stability, making it suitable for formulations requiring prolonged oxygen retention [368]. Oxygen‐labeled shikimate derivatives (^18^O) with siloxane linkages are being explored as traceable molecules for imaging and tracking oxygen delivery (Figure 14B) [369].

Nanoformulation plays a crucial role in oxygen‐releasing systems by enabling controlled, localized, and sustained oxygen delivery to hypoxic tissues [368]. Integration with nanotechnology has facilitated the development of stimuli‐responsive oxygen delivery platforms, where oxygen release is triggered by environmental cues such as pH, ROS, enzymes, or light, enabling precise spatiotemporal control. Encapsulated hemoglobin‐based oxygen carriers (HBOCs) are designed to mimic red blood cells and enhance oxygen transport. These systems include hemoglobin encapsulated within polymer shells, liposomes, or polymersomes (Figure 14C) [368]. However, during I/R, HBOCs lack protection against oxidative stress, causing tissue injury by generating superoxide [370, 371]. To overcome this, HBOCs combine oxygen delivery with antioxidant enzymes such as superoxide dismutase (SOD) and catalase, offering protection against I/R damage [371].

Oxygen delivery systems are crucial in regenerative medicine and cancer therapy. Early attempts fabricated hemoglobin‐loaded microparticles (Hb‐MPs) through co‐precipitation, cross‐linking, and calcium carbonate dissolution for controlled oxygen release [372, 373]. Enzymatic systems, such as catalase‐loaded NPs, can convert endogenous H_2_O_2_ into O_2_, providing site‐specific oxygen generation in tissues with elevated ROS levels, such as tumors or inflamed regions [374]. In addition, oxygen‐carrying nanomaterials have been developed, including PFC‐based emulsions, hemoglobin‐ or myoglobin‐loaded NPs, MOFs, and liposomes, which physically dissolve or chemically bind oxygen and release it in a controlled manner [375, 376]. Laser‐triggered PFC nanoplatforms (PFPLNPs) and PFC NPs (PFPCNPs) are being developed for targeted oxygen release in hypoxic tumors, aiding in the delivery of nucleic acids and therapeutic proteins in cancer models [377]. PFC‐based oxygen carriers (PFOCs), such as perfluorodecalin, are stabilized in emulsions by lecithin or block copolymer shells for efficient oxygen transport and release in tissues (Figure 14C) [368]. Scientists have effectively developed stem cell‐based oxygen carriers (SCOCs) and demonstrated their therapeutic potential, particularly in areas such as blood disorders and tissue engineering. Stem cell technology has enabled the production of red blood cell (RBC)‐like cells from various cell sources, including mesenchymal stem cells (MSCs) and induced pluripotent stem cells (iPSCs). iPSCs can be generated from somatic cells and offer a potential alternative source for blood transfusions and a tool for modeling and treating blood disorders (Figure 14C). For example, HEMOXCell, a RBC substitute derived from MSCs and grown in a medium containing human platelet lysate, shows promise for use in tissue engineering. Its potential lies in acting as a natural oxygen carrier to promote cell growth and viability in hypoxic (low‐oxygen) environments [358]. Oxygen micro/nanobubbles (OMNBs) are minute gas‐filled spheres that can travel through blood vessels and are being developed for therapeutic purposes. This nanotechnology enables the delivery of oxygen and other therapeutic agents to treat various medical conditions, particularly those involving low‐oxygen environments (hypoxia). OMNBs release oxygen upon ultrasound‐triggered cavitation and diffusion (Figure 14C) [378, 379].

**FIGURE 15:**
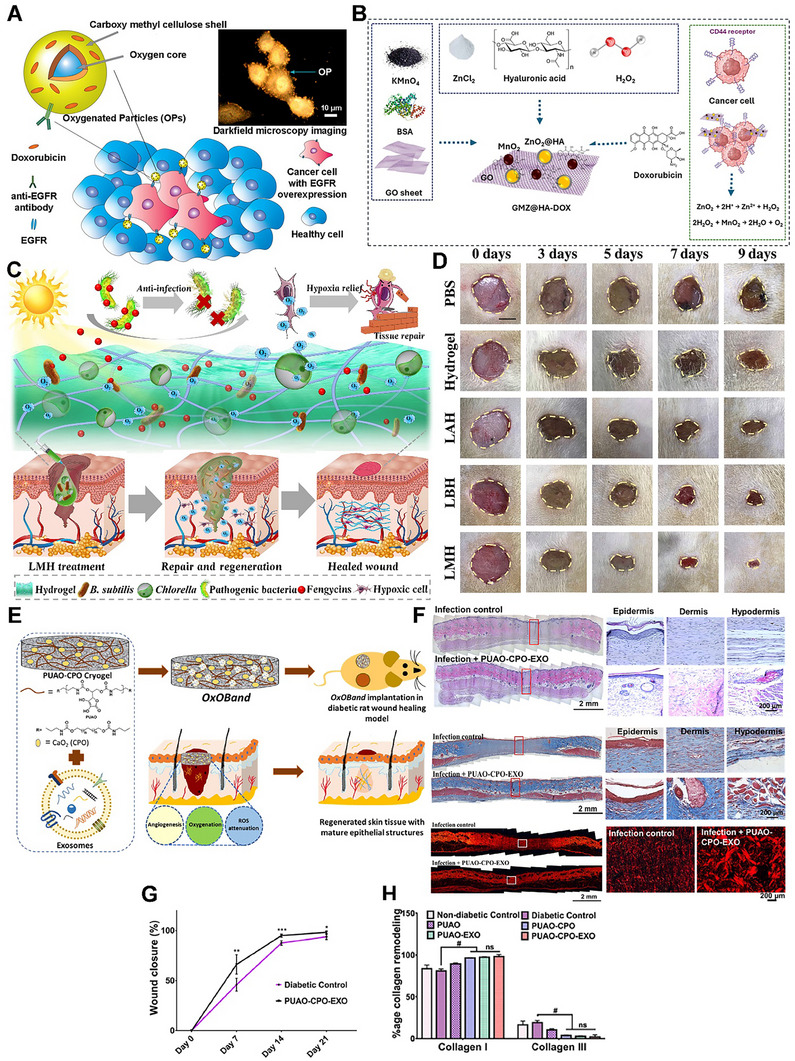
Oxygen delivery systems and their potential applications. (A) Illustration of oxygenated particles targeting tumor cells. Adapted under the Creative Commons CC BY‐NC‐ND 4.0 [380]. Copyright 2022, American Chemical Society. (B) A diagram illustrating the preparation of GMZ@HA‐DOX and its O_2_ generation in the tumor microenvironment. Reproduced with permission [381]. Copyright 2025, Elsevier. (C) Schematic illustrations of the application of the LMH for hard‐to‐heal wounds. (D) Representative images of full‐thick skin wounds treated with PBS, hydrogel, LAH, LBH, and LMH Adapted under the Creative Commons Attribution CC BY‐NC [37]. Copyright 2023, Science AAAS. (E) Diagram illustrating the formation of OxOBand from oxygen‐releasing antioxidant PUAO‐CPO cryogels loaded with adipose‐derived stem cell exosomes. (F) Histological images of wound healing after OxOBand administration: H&E (top), Masson trichrome (middle), and Picrosirius red (bottom). (G) Graph showing % wound closure. (H) Quantification of collagen I and collagen III remodeling. Reproduced with permission [382]. Copyright 2020, Elsevier.

Targeted oxygen delivery to cancer cells can be achieved using oxygen‐cored NPs functionalized with anti‐epidermal growth factor receptor (EGFR) antibodies (Figure 15A) [380]. A recent study in cancer theranostics developed a multifunctional nanocomposite (GMZ@HA‐DOX) to relieve tumor hypoxia and boost doxorubicin (DOX) efficacy (Figure 15B) [381]. Graphene oxide loaded with MnO_2_ and hyaluronic acid (HA)‐coated ZnO_2_ generated oxygen in acidic tumor environments, improving drug release and oxygenation. The system enhanced DOX uptake, ROS production, and apoptosis in breast cancer cells while showing low toxicity and effective tumor penetration. Light‐mediated hydrogels (LMH) generate oxygen upon light exposure, promoting pathogen clearance, relieving hypoxia, and facilitating tissue regeneration through Chlorella, hydrogel networks, and photosensitive components. Studies have shown superior wound healing with LMH‐treated wounds compared to control groups (Figure 15C,D) [41]. In an interesting study, exosome‐laden OxOBand, an innovative cryogel wound dressing, was developed and studied to address the complex healing challenges of diabetic and infectious wounds (Figure 15E) [382]. The OxOBand is a porous cryogel composed of antioxidant polyurethane, infused with calcium peroxide, for sustained oxygen release. OxOBand accelerated wound closure, collagen deposition, re‐epithelialization, and neovascularization while reducing oxidative stress within two weeks in diabetic wounds (Figure 15F–H). Other oxygen‐generating scaffolds, such as OGM3 scaffolds (PCL‐CaO_2_ microparticles suspended within GelMA, 60 mg/mL CaO_2_ in PCL and 8.1 mg/mL CaO_2_ in GelMA), showed the highest metabolic activity over 35 days across all culture conditions [383]. These scaffolds can also improve the efficacy of islet transplantation by reducing the hypoxic period between implantation and the development of functional vasculature [384].

Despite these advances, several challenges remain, including optimizing oxygen loading capacity, preventing premature release, ensuring biocompatibility and biodegradability, and achieving targeted delivery to dynamic hypoxic tissues. Future directions involve the development of multifunctional, stimuli‐responsive, and biodegradable carriers that integrate oxygen release with other gasotransmitters or therapeutic modalities, coupled with imaging‐guided strategies for personalized oxygen therapy. Overall, oxygen‐releasing agents and carriers represent a versatile and powerful class of therapeutic tools that address hypoxia‐related pathologies, enhance tissue repair, and improve the efficacy of conventional therapies. By leveraging nanotechnology and controlled release strategies, oxygen‐based therapeutics offer a precise, localized, and safe approach to restoring tissue oxygenation and supporting clinical interventions across a wide range of diseases.

### 4.4 Other Gas Therapeutics

Beyond well‐studied gasotransmitters such as NO, CO, and H_2_S, several traditionally overlooked or inert gases have recently been recognized for their unique therapeutic potential. Among these, CO_2_, H_2_, SO_2_, and Xe have emerged as promising candidates for biomedical applications due to their distinctive physiological effects and safety profiles.

**FIGURE 16:**
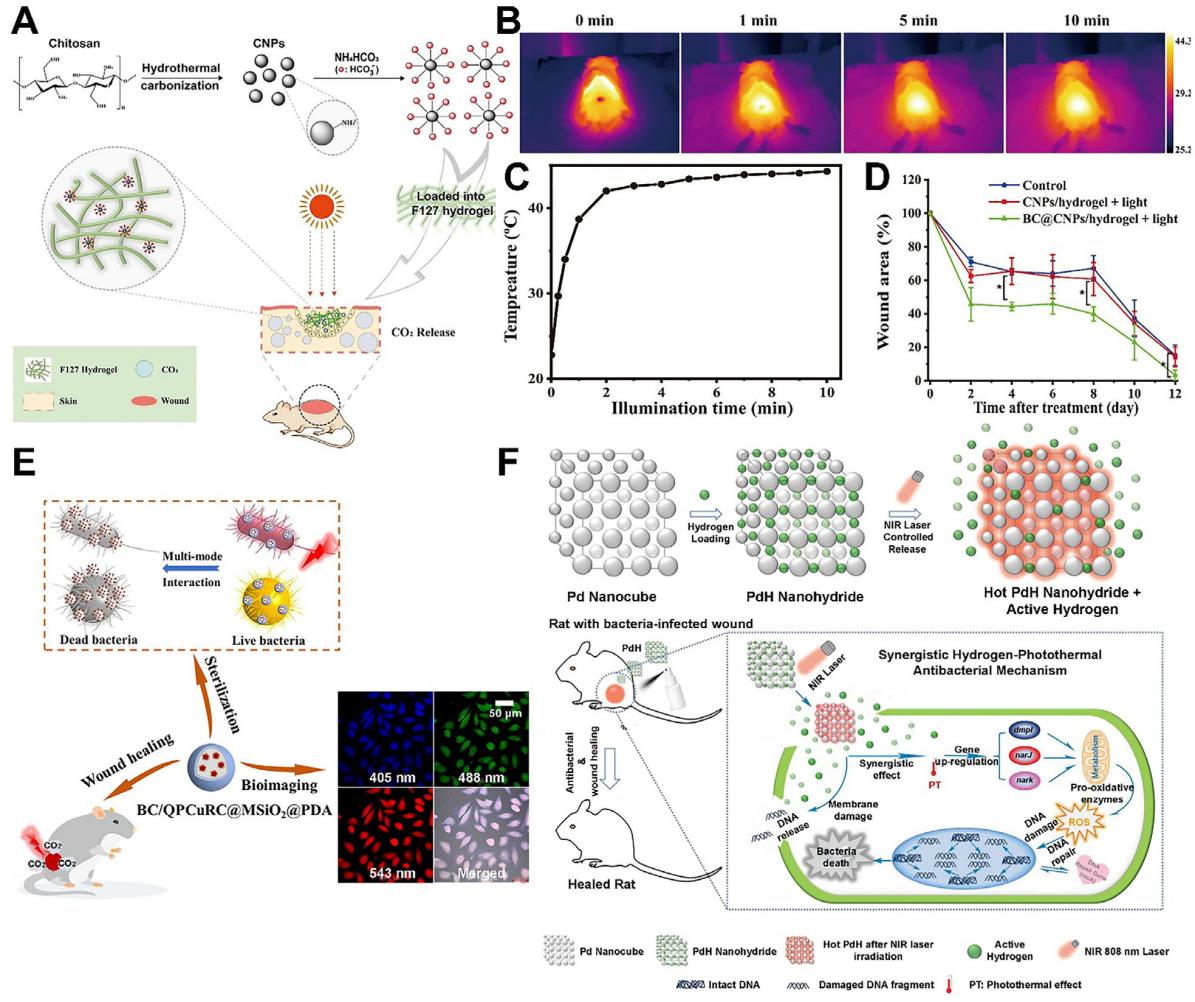
Carbon dioxide‐ and hydrogen‐releasing nanoplatforms. (A) Schematic showing BC@CNPs preparation and light‐triggered CO_2_ release from the photothermal hydrogel at wound sites. (B) Thermal images showing temperature changes in mice treated with BC@CNPs/hydrogel under simulated solar light over time. (C) Photothermal response of BC@CNPs/hydrogel at the wound site over 10 min of solar light irradiation. (D) Measurement of wound area over time following treatment. Reproduced with permission [392]. Copyright 2021, Elsevier. (E) Schematic depicting the synthesis of BC/QPCuRC@MSiO2@PDA and its associated biological applications. Reproduced with permission [393]. Copyright 2022, Elsevier. (F) Diagram showing the wound‐healing and antibacterial effects of PdH nanohydride in bacteria‐infected rat wounds. Reproduced with permission [401]. Copyright 2019, John Wiley and Sons.

CO_2_, long regarded as a metabolic waste product, is now recognized as a potential gasotransmitter with critical roles in cellular signaling and physiological regulation. Endogenously produced during respiration, CO_2_ freely diffuses across biological membranes and modulates diverse processes through both direct carbamylation of proteins and indirect effects via its hydrated forms (carbonic acid and bicarbonate), which influence pH‐sensitive enzymes, ion channels, and signaling cascades [385]. It activates soluble adenylyl cyclase (sAC) via bicarbonate, promoting cyclic adenosine monophosphate (cAMP) generation that regulates mitochondrial metabolism, neuronal activity, and sperm motility [386]. In the nervous system, CO_2_ acts as a key chemoregulatory signal maintaining respiratory drive, while in the vasculature, it induces vasodilation and enhances tissue oxygenation [387]. Additionally, elevated CO_2_ levels have anti‐inflammatory properties, attenuating NF‐κB activation and cytokine release in immune cells [388]. Recent advances in nanotechnology have enabled the development of CO_2_‐releasing nanomaterials, such as carbonate‐based NPs, enzyme‐loaded hydrogels, and polymeric carriers that release CO_2_ in a controlled and localized manner. These smart materials can regulate tissue microenvironments by promoting angiogenesis, reducing oxidative stress, and restoring pH balance, offering novel therapeutic strategies for wound healing, ischemic injury, and inflammatory diseases [389]. Li et al. developed a metal ion‐ligand coordination‐based nanoarchitecture, enabling NIR light‐triggered CO_2_ generation for therapeutic purposes [390]. The treatment involved simply applying the colloidal solution topically to the incisional wound, followed by NIR lamp exposure to induce CO_2_ release, which consequently accelerated the wound healing process. In another study, rattle‐type Fe_3_O_4_@CuS NPs with broad NIR absorption (700–1300 nm) were developed for PTT [391]. The in vitro photothermal experiments demonstrated that achieving the same level of cellular damage with 808 nm irradiation required doubling the laser intensity compared to 1064 nm irradiation. Guided by the magnetic Fe_3_O_4_ core, in vivo tumor ablation showed that 1064 nm irradiation eliminated tumors with no relapse, whereas 808 nm irradiation only suppressed growth for up to 30 days without full removal. In a different study, PEGylated hollow copper sulfide/bicarbonate (h‐CuS/BC) colloids were applied topically to the incisional wound and exposed to NIR light, resulting in 2‐cm wounds healing 5 days faster than the control group [391]. This improvement was attributed to NIR‐triggered CO_2_ release via the Bohr effect (a decrease in blood pH enhances oxygen release, thereby meeting tissue oxygen requirements), thereby accelerating tissue repair. Owing to the success of these NP formulations, hydrogels combined with CO_2_ therapy have also been investigated. Xie et al. developed a photothermal BC@CNPs hydrogel by combining BC‐loaded thermoresponsive Pluronic F127 with amino‐functionalized carbon NPs (Figure 16A) [392]. When applied to full‐thickness wounds and exposed to light, the hydrogel generated heat, inducing BC decomposition and localized CO_2_ release at the wound site (Figure 16B,C). Furthermore, BC@CNPs/hydrogel showed promising wound closure attributed to enhanced microcirculatory flow and elevated oxygen concentration in tissues (Figure 16D). Inspired by this study, Chu et al. developed the first silica‐supported nanoplatform (BC/QPCuRC@MSiO_2_@PDA) that integrates NIR carbon dots, BC, and phototherapy to promote wound healing (Figure 16E) [393]. Applied to S. aureus‐infected wounds, this system enabled controlled CO_2_ release under 808 nm irradiation while providing both PTT and PDT. As a result, complete wound closure was achieved within 14 days. Collectively, these findings position CO_2_ not merely as a metabolic byproduct but as a biologically active gasotransmitter with promising implications in nanomedicine and regenerative therapy.

H_2_, once considered biologically inert, is now recognized as a potential gasotransmitter due to its ability to: (i) safely scavenge intracellular hydroxyl radicals and peroxynitrite, reducing inflammation‐related ROS levels without toxicity, even at high concentrations; (ii) cross both the blood‐brain and blood‐eye barriers, efficiently diffusing through biological membranes; and (iii) exert anti‐inflammatory, antiallergic, and antiapoptotic effects, while enhancing energy metabolism [394]. Early studies reported that H_2_ can be delivered via H_2_ gas, H_2_‐rich water (HRW), or H_2_‐rich saline (HRS) [395]. In burns, alkali eye injuries, and radiation‐induced skin damage, H_2_ therapy decreases oxidative stress, modulates cytokine expression, and inhibits apoptosis, promoting tissue repair [394]. Its effects involve pathways such as nuclear factor erythroid 2‐related factor 2 (Nrf2)/heme oxygenase‐1 (HO‐1) and protein kinase B (Akt)/NF‐κB, highlighting H_2_’s antioxidant, anti‐inflammatory, and antiapoptotic roles in accelerating healing [396, 397]. Due to the volatile nature of H_2_ and the low tissue penetration of HRW and HRS therapies, H_2_‐releasing materials gained focus. A hydrogen‐producing hydrogel patch, composed of living Chlorella and bacteria within a cell‐impermeable casing, continuously generates H_2_ for 60 h [398]. This microbe‐hydrogel system selectively scavenges toxic •OH and ONOO^−^, reduces inflammation, and promotes cell proliferation. In diabetic wounds, it accelerated healing by ∼50% by day 3. With excellent biocompatibility and ROS‐scavenging capacity, the symbiotic hydrogel shows strong potential for clinical applications. However, the hydrogel patch has a limited lifespan, and using *Chlorella* raises biosecurity concerns. Addressing this, hydrogen‐doped titanium oxide (TiO_2_) nanorods (HTON) with a rutile single‐crystal structure were synthesized via a full‐solution incorporation method [399]. Hydrogen incorporation tuned its band structure, enabling Vis‐light absorption and photocatalytic activity. HTON used glucose in high‐glucose diabetic environments as a sacrificial agent, achieving controlled glucose depletion and hydrogen generation in vitro and in vivo. This reduced high‐glucose‐induced apoptosis and promoted cell proliferation and migration, supporting diabetic wound healing. HTON exhibited both strong therapeutic efficacy and high biosafety under Vis‐light irradiation. In another study, a multicomponent liposomal nanoreactor (Lip NR) containing chlorophyll a, l‐ascorbic acid, and gold NPs was developed to produce H_2_ gas in situ upon light exposure, similar to photosynthesis [400]. This localized H_2_ generation reduces ROS and pro‐inflammatory cytokines, mitigating inflammation in RAW264.7 cells and LPS‐induced paw inflammation in mice. Histological analysis confirmed reduced tissue inflammation, demonstrating the Lip NR system's potential to alleviate oxidative stress and promote anti‐inflammatory effects. To explore the ability of regulated release of H_2_, a biocompatible palladium hydride (PdH) nanohydride was developed by incorporating hydrogen into Pd nanocubes, enabling on‐demand H_2_ release under NIR irradiation (Figure 16F) [401]. By combining the therapeutic effects of bioactive H_2_ with the photothermal properties of Pd, the nano‐hydride demonstrates strong antibacterial activity both in vitro and in vivo. In rat models with severe bacterial infections, the synergistic H_2_ photothermal treatment effectively accelerates wound healing. Mechanistic studies indicate that its antibacterial action involves two pathways: upregulation of bacterial oxidative metabolism genes, generating ROS that induce DNA damage, and direct disruption of bacterial membranes, leading to the release of intracellular components. Despite extensive research, H_2_ therapy faces challenges in delivery, efficacy, and understanding its cellular mechanisms, while ensuring biocompatibility and stability. Combining it with existing treatments may enhance therapeutic outcomes.

SO_2_, an environmentally hazardous gas, is now recognized as an endogenous gasotransmitter alongside NO, CO, and H_2_S, playing key roles in regulating cellular functions, particularly in the cardiovascular system. SO_2_ acts synergistically with NO and exhibits similar vasodilatory effects [402]. It is widely used as an antibiotic, preservative, and antioxidant, often in hydrated forms such as bisulfite (HSO_3_^−^) and sulfite (SO_3_^2−^) [403]. Endogenously, SO_2_ can be generated through the oxidation of _L_‐cysteine by cysteine dioxygenase (CDO), producing cysteine sulfite, which further decomposes to SO_2_ and pyruvate. Additionally, SO_2_ arises from H_2_S metabolism: enzymatically oxidized H_2_S forms thiosulfate (S_2_O_3_^2−^), which reacts with glutathione (GSH) via thiosulfate reductase to produce SO_2_ and glutathione persulfide (GSSG), while GSSH can also be oxidized to SO_2_ by sulfur dioxygenase [403, 404]. Macrophage‐derived SO_2_ has anti‐inflammatory and mast cell‐stabilizing effects, but high levels generate reactive sulfite radicals that damage macromolecules and disrupt redox balance. This oxidative stress makes SO_2_ a promising antimicrobial agent [394]. SO_2_‐releasing materials have been investigated for treating bacterial infections, including Mycobacterium tuberculosis (Mtb), MRSA, and Enterobacter cloacae (MTCC 509) [405]. The thiol‐activated SO_2_ donor 2,4‐dinitrobenzenesulfonamide combats Mtb and MRSA by depleting intracellular thiols and inducing SO_2_‐mediated oxidative damage to lipids, proteins, and DNA. Thiol‐triggered activation complicates mechanistic studies, as SO_2_ can target multiple biologically relevant sulfides [394]. SO_2_ donors based on the 4,5‐dimethoxy‐2‐nitrobenzyl (DMNB) phototrigger release SO_2_ and hydroxyl compounds via C‐S bond photocleavage under single‐ or two‐photon activation [405]. Ferulic acid ethyl ester (FAEE), a hydroxy‐based drug with broad antibacterial activity and intrinsic fluorescence, enables self‐monitoring of release, with fluorescence intensity correlating to enhanced antibacterial effects against MTCC 509. Future studies may investigate other SO_2_ donors, including cell‐permeable esterase‐sensitive sulfonates and benzothiazolyl sulfite, for their potential in promoting trauma repair.

Xe is an inert noble gas recognized for its biological activity and potential as a gasotransmitter. Unlike classical gasotransmitters, Xe does not participate in chemical reactions but exerts effects through physical interactions with cellular targets, including ion channels and receptors. Xenon has been shown to provide neuroprotection, cardioprotection, and anti‐inflammatory benefits by modulating signaling pathways, reducing oxidative stress, and inhibiting excitotoxicity [406]. Its low toxicity and rapid diffusion make Xe a promising therapeutic gas in anesthesia, I/R injury, and tissue protection.

Surrounding NO, Figure 8 highlights its functional crosstalk with other gaseous mediators. Mechanistically, CO and H_2_S largely cooperate with NO to suppress excessive inflammation and promote cytoprotection and tissue repair [407, 408, 409]. H_2_ indirectly modulates NO signaling by limiting oxidative stress [410]. CO_2_ influences immune and vascular responses through pH‐dependent metabolic regulation [411]. SO_2_ and Xe fine‐tune inflammatory and stress‐response pathways in a context‐dependent manner [412, 413]. Together, these interacting gases regulate inflammation, autoimmunity, and tissue regeneration. The schematic emphasizes that harnessing these synergistic immunomodulatory effects requires precisely controlled, material‐enabled delivery systems to achieve localized, safe, and effective gas dosing.

## 5 Challenges and Future Perspectives

Building upon the therapeutic potential of gasotransmitters such as NO, CO, H_2_S, and emerging candidates like CO_2_, SO_2_, H_2_, and Xe, it is increasingly evident that their clinical translation relies not only on understanding their biological roles but also on mastering their controlled delivery. Despite remarkable innovations in gas‐releasing biomaterials and donor chemistry, several pressing challenges must be addressed to harness their therapeutic efficacy fully. One of the most critical issues is the precise regulation of spatiotemporal gas release within complex physiological environments [414]. Current platforms, including prodrugs, photocaged molecules, MOFs, and stimuli‐responsive nanocarriers, have greatly expanded the therapeutic toolkit. However, delivering gases selectively, on demand, and at clinically relevant doses remains difficult, particularly in sensitive tissues such as the brain, retina, myocardium, or inflamed pulmonary epithelium [414, 415]. A major barrier to the clinical translation of gas‐based therapeutics is ensuring long‐term safety, biocompatibility, and efficient metabolic clearance of delivery platforms (Table 3). Although nanostructured systems, including NPs, MOFs, and polymeric scaffolds, have demonstrated considerable promise in preclinical studies, their chronic biodistribution, degradation pathways, and potential accumulation in off‐target organs remain poorly understood. From our perspectives, addressing these issues will require rigorous toxicological assessments, such as long‐term dose‐response evaluations and fate‐mapping studies, alongside the development of biodegradable or self‐immolative carriers that decompose into non‐toxic metabolites. Adding further complexity, therapeutic gases rarely act in isolation. Gasotransmitters, such as NO, CO, H_2_S, H_2_, CO_2_, and O_2_, modulate interconnected pathways that include oxidative stress, mitochondrial homeostasis, angiogenesis, and immune responses. Co‐delivery strategies such as NO/H_2_S or O_2_/CO combinations may yield synergistic benefits or unintended antagonistic effects, depending on dose, timing, and the local microenvironment. Decoding these interactions demands deeper insights into gas‐cellular biology, platforms capable of independently modulating the release kinetics of multiple gases, and precise characterization of their pharmacokinetic and pharmacodynamic behaviors.

**TABLE 3:** Safety, toxicology, and translational constraints of NO delivery systems.

Risk category	Mechanistic basis	Most affected platforms	Key design constraints	Relative translational risk	Refs,.
Excess NO exposure	Mitochondrial inhibition, apoptosis	Small‐molecule donors, burst‐release systems Catalytic surfaces Enzyme‐activated prodrugs	Flux control Sustained release by using stimulus‐responsive, catalytic systems, immobilized or localized NO generation	High (if uncontrolled)	[11, 67, 438]
Reactive nitrogen species	Formation of ONOO^−^, NO_2_, and N_2_O_3_	Catalytic, photoactivated, high‐flux platforms	Physiological flux matching Control catalytic turnover rates Avoid excessive local NO accumulation Use biomimetic catalytic rates comparable to endogenous enzymes	Moderate–high	[11, 21, 43]
Systemic hypotension	Off‐target vasodilation	Systemic donors Circulating nanocarriers Enzyme‐activated prodrugs	Localized or immobilized delivery Tissue‐specific activation triggers	Moderate	[67, 437]
Material toxicity	Metal ions leaching Polymer degradation Nanoparticle accumulation and inflammatory responses Surface‐induced thrombogenicity or immune activation	MOFs, nanoparticles, implants	Biocompatible materials Stable immobilization Controlled degradation rates Minimization of toxic metal ion release	Platform‐dependent	[11, 439, 442, 443]
Chronic exposure effects	Tolerance, inflammation	Long‐term implants, continuous donors	Triggered catalytic generation Long‐term stability validation Chronic implantation studies	Low–moderate (if controlled)	[11, 444]

Bridging the gap between preclinical success and human application remains one of the most critical challenges in advancing gas‐based therapeutics. Although rodent models have been indispensable for early discovery, fundamental differences in anatomy, immune responses, and metabolic pathways often result in therapeutic effects that fail to translate to humans. We believe that overcoming this limitation will require the development and adoption of more human‐relevant preclinical platforms, such as patient‐derived organoids, organ/tissue‐on‐a‐chip systems, and large animal models, are essential to more accurately mimic human physiology and disease progression. Beyond biological translation, the path to clinical implementation is further complicated by manufacturing and regulatory challenges. Scaling up sophisticated gas‐delivery platforms, particularly MOFs, photodynamic nanocarriers, and hybrid scaffolds, requires Good Manufacturing Practice (GMP)‐compliant protocols to ensure consistency and reproducibility across batches [416]. At the regulatory level, combination products such as implantable gas‐releasing devices or wearable NO monitoring systems must meet rigorous standards applicable to both drug and medical device categories, making approval pathways highly complex.

Another key challenge in advancing gas‐based therapeutics is ensuring the stability and reliable performance of delivery systems within the complex and ever‐changing physiological environment [417]. Platforms that rely on light, magnetic fields, or biochemical triggers must remain functional despite variations in pH, enzyme levels, oxygen availability, and tissue penetration depth. To address these limitations, recent innovations include materials with NIR responsiveness, built‐in self‐reporting mechanisms such as fluorescence signals during gas release, and adaptive systems capable of dynamically responding to their microenvironment [415]. These technologies hold significant promise for achieving precise gas release, real‐time monitoring, and improved in vivo reliability. As gas delivery technologies advance toward clinical translation and commercial use, ethical, environmental, and economic considerations become increasingly important. The fabrication of some gas‐based platforms relies on rare‐earth elements, perfluorinated compounds, or energy‐intensive manufacturing steps, raising concerns about sustainability, costs, and long‐term accessibility. Therefore, future research must focus on eco‐conscious design, the use of abundant and biodegradable materials, and scalable, cost‐effective production methods. These efforts will be critical in ensuring that gasotransmitter‐based therapies are not only effective and safe but also sustainable and globally accessible.

Future progress will depend on integrating multifunctional strategies that combine active targeting ligands with externally controllable release cues (e.g., light, magnetic fields, ultrasound, pH, or enzymatic activity). Emerging smart delivery systems are also expected to incorporate real‐time sensing modules capable of detecting local gas concentrations or tissue‐specific biomarkers, enabling closed‐loop, self‐regulating theranostic platforms [418]. Additionally, scalability, long‐term stability, immune compatibility, and compliance with clinical manufacturing standards remain major barriers that must be addressed before gas‐based therapies can transition from experimental models to routine clinical applications. Together, these advances will shape the next generation of gasotransmitter‐based therapeutics, moving them closer to safe, personalized, and precision medicine. Looking ahead, the future of therapeutic gas systems lies in the development of multifunctional platforms capable of precise dosing, real‐time monitoring, and adaptive feedback within a single integrated design. Realizing this goal will demand highly interdisciplinary collaboration spanning materials science, synthetic chemistry, systems biology, biomedical engineering, and clinical medicine. By combining smart delivery technologies with predictive artificial intelligence (AI) and computational modeling, human‐relevant preclinical models, and environmentally sustainable manufacturing approaches, gas‐based therapies can evolve from experimental concepts into clinically transformative tools. Such advancements hold promises for broad applications in regenerative medicine, oncology, infectious diseases, and other emerging medical fields.

Taken together, these challenges underscore that the successful clinical translation of gas‐based therapeutics will depend not on isolated material innovations, but on integrated systems capable of precise dosing, adaptive control, long‐term safety, and regulatory scalability. From our perspective, the convergence of smart delivery materials, real‐time sensing, human‐relevant preclinical models, and sustainable manufacturing represents the most viable pathway for transforming therapeutic gases from experimental tools into routine clinical modalities.

## Conflicts of Interest

The authors declare no conflicts of interest.
